# Toward high-performance and stable blue perovskite light-emitting diodes via optimized fluorinated polymer blend hole transport layers

**DOI:** 10.1126/sciadv.aec7056

**Published:** 2026-08-28

**Authors:** Qichun Gu, Tianjun Liu, Yucheng Xu, Xinjuan Li, Yunzhou Deng, Yang Lu, Youcheng Zhang, Zimu Wei, Xinyu Bai, Capucine Mamak, Yutong Han, Alessandro J. Mirabelli, Weidong Xu, Jian Mao, Caterina Ducati, Henning Sirringhaus, Samuel D. Stranks, Miguel Anaya

**Affiliations:** ^1^ Department of Chemical Engineering and Biotechnology, University of Cambridge, Philippa Fawcett Drive, Cambridge CB3 0AS, UK.; ^2^ Cavendish Laboratory, University of Cambridge, JJ Thomson Avenue, Cambridge CB3 0HE, UK.; ^3^ Department of Materials Science and Metallurgy, University of Cambridge, Cambridge, UK.; ^4^ State Key Laboratory of Photovoltaic Science and Technology, Shanghai Frontiers Science Research Base of Intelligent Optoelectronics and Perception, College of Future Information Technology, Institute of Optoelectronics, Fudan University, Shanghai, China.; ^5^ Instituto de Ciencia de Materiales de Sevilla, Department of Condensed Matter Physics, Universidad de Sevilla-CSIC, Calle Americo Vespucio 49, Sevilla 41092, Spain.

## Abstract

Perovskite light-emitting diodes (PeLEDs) are promising low-cost, solution-processable, and color-pure optoelectronic devices for display and lighting applications. However, blue PeLEDs continue to lag their green and red counterparts in terms of luminance and operational lifetime, limiting their practical implementation. The performance disparity primarily arises from charge injection imbalance, which accelerates degradation at the perovskite/hole transport layer (HTL) interface under electrical bias. Here, we design a polymer blend HTL comprising poly(*N*-vinyl-2,7-difluoro-carbazole) (PVK-F) and poly(bis(4-phenyl)(2,4,6-trimethylphenyl)amine) (PTAA), in which strengthened van der Waals interactions promote denser molecular packing. This optimized microstructure simultaneously enhances hole mobility and wettability, enabling high-quality perovskite film formation. Consequently, PeLEDs emitting at 485 nanometers achieve an external quantum efficiency of 24.1% and luminance exceeding 26,000 candela per square meter. Moreover, the improved hole injection mitigates interfacial degradation, yielding an operational half-lifetime exceeding 700 minutes at an initial luminance of 100 candela per square meter. This work establishes polymer blending as an effective strategy for advancing the performance and stability of blue PeLEDs.

## INTRODUCTION

Organic-inorganic halide perovskite materials (OIHPs) are promising candidates for next-generation display and lighting technologies owing to their exceptional optoelectronic properties, such as high photoluminescence quantum efficiency (PLQE), narrow emission linewidth, and tunable bandgap covering the visible to near-infrared spectrum range (*1*, *2*). Light-emitting diodes (LEDs) based on OIHPs have demonstrated remarkable performance in the green, red, and near-infrared spectral regions, achieving external quantum efficiency (EQE) exceeding 25% and operational stability beyond 300 hours (*3*–*5*), thereby narrowing the performance gap with the state-of-the-art organic and quantum-dot LEDs (*6*–*9*). Despite the recent advances in sky-blue PeLEDs, which have reached EQEs of 23% using quantum dots (QDs), nanocrystals (NCs), or bulk three-dimensional (3D) and quasi-2D perovskites composed of mixed-2D/3D components as the emissive layer (*10*–*13*), these PeLEDs remain limited by the low luminance [typically below 5000 cd m^−2^ (*10*, *11*, *13*)] and poor operational lifetime [less than 2 hours (*11*, *13*)].

These performance bottlenecks primarily arise from the intrinsic structural instability associated with the ionic nature of halide perovskites, low PLQE resulting from increased chloride incorporation, inefficient carrier injection into the emissive layer, and bias-induced degradation at the perovskite/transport layer interface (*14*–*17*). Efforts to overcome these challenges have focused on fabricating highly luminescent NCs, QDs, and mixed-2D/3D perovskite emissive layers with PLQE above 90% (*18*, *19*). However, insulating bulky organic ligands used in these materials to either stabilize colloids or confine the excitons impede charge injection, thereby limiting the achievable luminance in devices. In addition, ligand detachment in NCs and QDs and phase rearrangement in mixed-2D/3D perovskites under electrical bias accelerate both spectral and operational degradation (*13*, *20*, *21*). Alternatively, 3D bulk perovskite emitters, CsPbBr*_x_*Cl_3-*x*_, have shown great promise for achieving higher luminance and improved spectral stability in blue PeLEDs compared with other emitter types discussed above. The absence of insulating organic ligands in 3D perovskites facilitates charge injection into the emissive layer, contributing to a higher luminance. In addition, vapor treatment strategies can suppress the formation of Cl-rich regions in pristine 3D emissive films, contributing to stabilized electroluminescence (EL) spectra under an applied bias of 6.5 V (*15*, *22*). Despite these advances, such devices still suffer from severe nonradiative recombination losses, especially at the interface between the perovskite emitter and the underlying hole transport layers (HTLs). This interface critically influences perovskite crystallization, film morphology, and defect formation, ultimately limiting device efficiency and operational lifetime (*23*, *24*).

Beyond the limitations of the perovskite emitters, inefficient hole injection remains a major bottleneck in blue PeLEDs, restricting both device luminance and operational lifetime. This challenge primarily arises from the higher energy barriers for hole injection compared to electron injection, coupled with substantially lower hole mobilities of typical HTL materials relative to electron mobilities in electron transport layer (ETL) materials (*25*, *26*). Such a pronounced imbalance in charge injection leads to excessive electron accumulation within the emissive layer, which accelerates interfacial degradation at the perovskite/HTL interface and shortens device operational lifetime (*27*, *28*). These issues highlight the critical need for optimized HTLs and refined interfacial interactions with the perovskite emitters.

Common HTLs applied in blue PeLEDs include both inorganic nanoparticle-based and polymeric materials. Inorganic nanoparticle-based HTLs, such as nickel oxide (NiO*_x_*), exhibit high hole mobility, which effectively facilitates hole injection into the perovskite layer for enhanced emission (*29*, *30*). However, their performance remains suboptimal compared to polymer-based analogs due to the large energy mismatches with blue-emitting perovskites and dopant-induced carrier quenching (*15*, *31*, *32*). Polymer-based HTLs, such as poly(3,4-ethylenedioxythiophene) polystyrene sulfonate, also provide high hole mobility and have enabled blue PeLEDs with EQE above 20%. Nevertheless, their acidic and hygroscopic nature compromise perovskite stability, limiting the operational lifetimes to under 10 min (*11*). Other commonly used polymeric HTLs, including poly(9-vinylcarbazole) (PVK), poly(9,9-dioctylfluorene-alt-*N*-(4-sec-butylphenyl)-diphenylamine) (TFB), and poly(bis(4-phenyl)(2,4,6-trimethylphenyl)amine) (PTAA), typically exhibit hole mobilities ranging from 10^−6^ to 10^−4^ cm^2^ V^−1^ s^−1^, one or two orders of magnitude lower than electron mobilities in commonly used ETLs (*26*). Moreover, their hydrophobic surfaces hinder uniform deposition of bulk 3D perovskite layers, further exacerbating nonradiative recombination losses. Although polymer-blend strategies involving PVK and TFB have been explored to suppress interfacial nonradiative recombination, the associated changes in surface wettability, interfacial energetics, and charge transport properties remain poorly understood (*33*).

Here, we report blue PeLEDs that simultaneously deliver high EQE, high luminance, and improved operational lifetime by incorporating a polymer blend HTL comprising PTAA and poly(*N*-vinyl-2,7-difluoro-carbazole) (PVK-F). Owing to the compact molecular packing arising from favorable interactions between the conjugated carbazole and methylphenyl units, the polymer blend exhibits enhanced hole mobility compared with the individual constituents. Benefiting from these synergistic properties, the resulting devices demonstrate performance that surpasses previously reported blue PeLEDs.

## RESULTS

### Effects of HTLs on device performance and stability

**Fig. 1.**
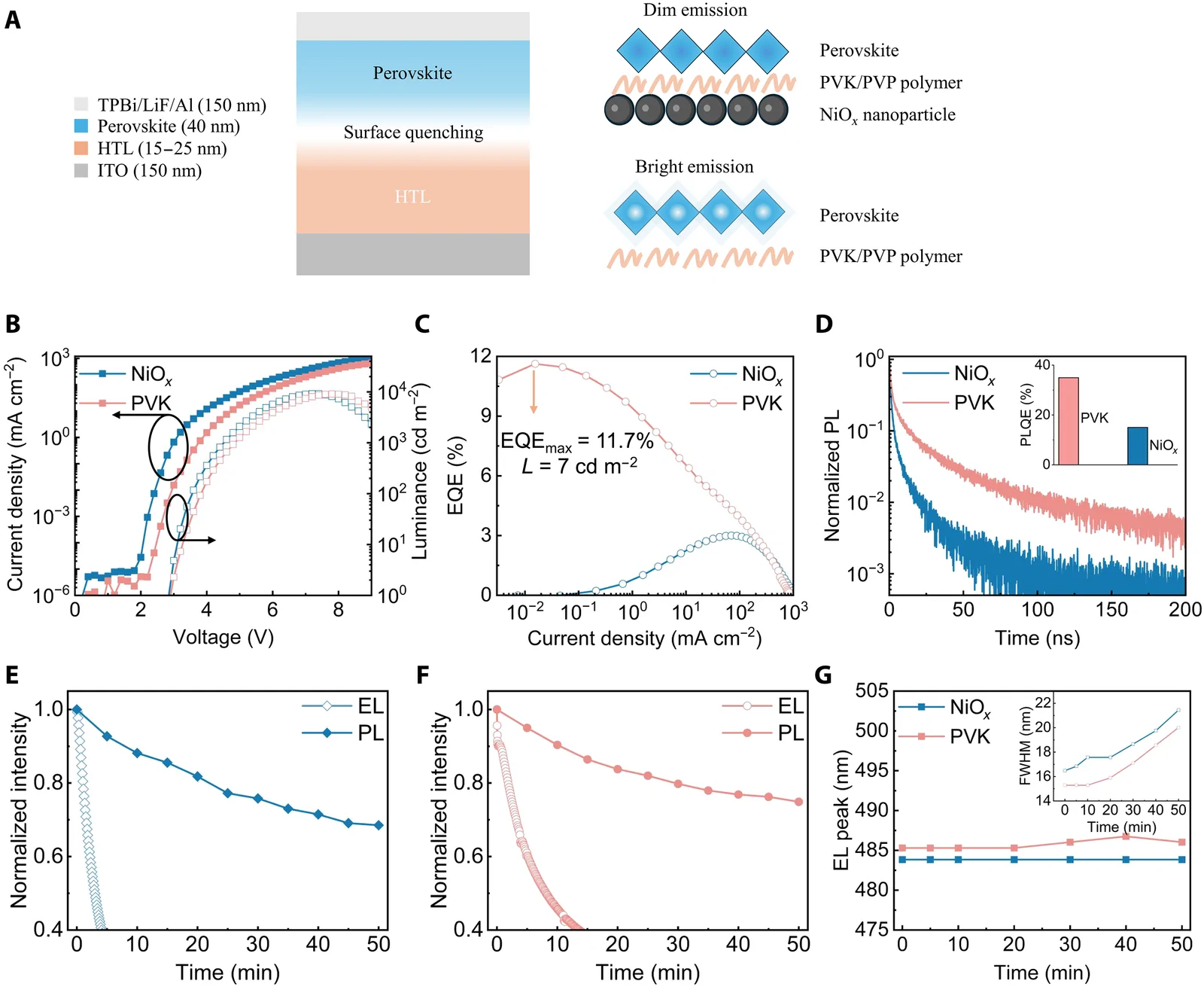
HTLs induce surface quenching in blue PeLEDs. (**A**) Device structure and the two corresponding different HTL configurations. (**B**) Current density-voltage-luminance characteristics for PeLEDs with NiO*_x_*/PVK/PVP (NiO*_x_*-based) and PVK/PVP (PVK-based) as HTL configurations. (**C**) EQE–current density curves of NiO*_x_*-based and PVK-based PeLEDs. (**D**) TRPL for NiO*_x_*-based and PVK-based perovskite films excited by a 405-nm pulsed laser at a fluence of 0.12 nJ cm^−2^ per pulse. Inset displays the corresponding PLQEs. (**E** and **F**) PL and EL stability tests for NiO*_x_*-based and PVK-based PeLEDs. For EL stability, devices were biased at constant current density to achieve an initial luminance of 100 cd m^−2^. For the PL stability, devices were measured under 405-nm continuous laser excitation at 16 and 2.9 W cm^−2^ for NiO*_x_*- and PVK-based HTL configurations, respectively, to generate photogenerated current densities similar to those in EL stability measurements. (**G**) EL peak position and FWHM (inset) evolution for NiO*_x_*-based and PVK-based PeLEDs under constant current density bias.

We first examine the influence of HTL configuration on the performance and operational lifetime of blue PeLEDs. As depicted schematically in Fig. 1A, the device architecture is composed of indium tin oxide (ITO; 150 nm)/NiO*_x_* (with or without, ∼10 nm)/PVK (∼10 nm)/poly(4-vinylpyridine) (PVP; ∼1 nm)/perovskite (∼40 nm)/2,2′,2″-(1,3,5-benzinetriyl)-tris(1-phenyl-1-*H*-benzimidazole) (TPBi; 50 nm)/lithium fluoride (LiF; 1.5 nm)/Al (100 nm). Here, NiO*_x_* is introduced to provide favorable highest occupied molecular orbital (HOMO) energy alignment between ITO and PVK, creating a cascade energy profile that facilitates efficient hole injection (*15*, *32*). A thin PVP interlayer is used to improve the wettability of the perovskite precursor solution and promote uniform film coverage after spin coating (*15*). The perovskite emissive layer is deposited by spin-coating the precursor solution composed of cesium bromide (CsBr):formamidinium bromide (FABr):lead bromide (PbBr_2_):lead chloride (PbCl_2_):4,7,10-trioxa-1,13-tridecanediamine (TTDDA) in a molar ratio of 1.2:0.3:0.3875:0.6125:0.1 (abbreviated as FACsPbBr*_x_*Cl_3-*x*_) dissolved in dimethyl sulfoxide (DMSO), followed by vapor treatment with *N*,*N*-dimethylformamide (DMF) in an enclosed petri dish for 9 min (*15*).

The resulting perovskite films deposited on NiO*_x_*/PVK/PVP (denoted as NiO*_x_*-based) and PVK/PVP (denoted as PVK-based) HTL configurations exhibit island-shaped morphology (fig. S1) and photoluminescence (PL) emission peaks at 483 and 484 nm, respectively (fig. S2). NiO*_x_*-based films display a broader PL full width at half maximum (FWHM) of 16 nm compared with 14 nm for the PVK-based films. Grazing-incidence wide-angle x-ray scattering (GIWAXS) analysis confirms identical perovskite crystal structures on both HTL configurations, with no additional phases or peak shifts observed in either case (fig. S3). Perovskite films exhibit characteristic scattering peaks at *q*-values of 1.09 and 1.51 Å^−1^, corresponding to the (100) and (110) planes of the orthorhombic perovskite structure (*15*). Despite similar perovskite morphologies and crystal structures, pronounced differences are observed in device performance. Both NiO*_x_*- and PVK-based PeLEDs achieve comparable maximum luminance of around 9000 cd m^−2^ (Fig. 1B), with EL peaks at 484 and 485 nm, respectively (fig. S4), where the slight EL emission peak shift could be ascribed to the HTL-induced variations in perovskite crystallization (*34*). However, NiO*_x_*-based devices exhibit higher current densities across the entire voltage range, consistent with the superior hole mobility of NiO*_x_* compared to polymer-based HTLs (*30*). These electrical advantages, however, do not translate into improved efficiency, as NiO*_x_*-based PeLEDs show a substantially lower EQE of around 3.4% compared with 11.7% for PVK-based PeLEDs (Fig. 1C).

To understand the origin of these drastic differences in performance, time-resolved photoluminescence (TRPL) measurements are conducted at an excitation fluence of 0.12 nJ cm^−2^ per pulse, corresponding to the nonradiative recombination-dominant regime. As shown in Fig. 1D, perovskite films deposited on NiO*_x_*-based HTLs exhibit markedly faster TRPL decay, which indicates more nonradiative recombination channels via either trap states or charge transfer processes compared to perovskite films on PVK-based HTLs (see fluence-dependent TRPL in fig. S5) (*35*). Notably, this fast TRPL decay in NiO*_x_*-based samples can be partially alleviated by introducing a thicker PVK layer on top of NiO*_x_* (fig. S6A), where TRPL decay dynamics of perovskites deposited on NiO*_x_*/PVK (25 nm)/PVP HTL configuration fall between those of NiO*_x_*/PVK (10 nm)/PVP and PVK/PVP HTL configurations. This TRPL trend is correlated with the PLQE measurements for these HTL stacks (fig. S6B). The PLQE increases upon introducing a thicker PVK on top of NiO*_x_* and reaches its highest value when the NiO*_x_* layer is completely removed. At the same time, PVK can be washed away by DMSO during perovskite deposition (*36*), and it is important to distinguish intrinsic interfacial luminescence quenching from possible solvent-induced damage to the PVK layer. We therefore examine the solvent stability of PVK in DMSO and find that PVK is almost fully dissolved at 8 mg ml^−1^ (fig. S7). Thickness measurements on NiO*_x_*/PVK/PVP films after DMSO washing show only a slight reduction, indicating partial rather than complete washing of PVK (fig. S8). In addition, the EQE-current-density behavior of PVK-based devices remains clearly different from that of direct ITO/perovskite devices, confirming that the PVK remains functionally present after perovskite deposition. These PVK solubility tests, TRPL, and PLQE results indicate that DMSO-induced PVK loss may contribute to the poorer performance of NiO*_x_*-based devices; however, NiO*_x_* intrinsically induces additional carrier quenching, which cannot be fully suppressed even with increased interlayer thickness.

To further evaluate the effects of HTLs on the operational lifetime of blue PeLEDs, we perform EL and PL stability tests (Fig. 1, E and F) on full device structures using the two HTL configurations. For EL operational lifetime tests, devices are operated at a constant current density of 4.4 and 0.8 mA cm^−2^ for NiO*_x_*- and PVK-based PeLEDs, respectively, to achieve the same initial luminance of 100 cd m^−2^. Devices fabricated on both HTL configurations reveal pronounced EL degradation, with NiO*_x_*-based devices degrading more rapidly and exhibiting a T_50_ of only 3 min, compared with 8 min for PVK-based devices. Although the EL peak positions remain consistent after 50 min of continuous biasing for both devices, a notable broadening in FWHM is observed (Fig. 1G), which may arise from phase segregation or interfacial degradation between the HTL and the emissive layer (*15*–*17*, *37*). In contrast, for PL stability tests, device active areas are illuminated under a continuous 405-nm laser at a power density of 16 and 2.9 W cm^−2^ for NiO*_x_*- and PVK-based devices, respectively, to generate comparable photogenerated current densities to those in EL stability measurements (setup illustrated in fig. S9). After the same 50-min laser excitation, the PL intensity remains above 70% of its initial value in both configurations, indicating far longer PL stability compared to the EL stability (see PL decay trends at higher laser excitation in fig. S10). Despite the slight reduction in PL intensity, the emission peak positions and FWHM remain stable throughout the illumination process as shown in fig. S11. A similar difference between PL and EL decay is observed in operando PL-EL measurements, where PL is captured while the devices are under bias (fig. S12). In these measurements, perovskites deposited on both NiO*_x_*-based and PVK-based HTL configurations demonstrate faster EL decay compared to PL decay. These marked discrepancies between PL and EL spectral stability and lifetime further suggest that the operational degradation is not solely governed by the intrinsic perovskite instability but is also driven by charge injection processes and degradation at the perovskite/HTL interface.

The confirmed higher PLQE, longer PL and EL stability, and slower TRPL decay in PVK-based films and devices compared to their NiO*_x_*-based counterparts suggest that although NiO*_x_* enhances hole injection efficiency, it simultaneously exacerbates interfacial nonradiative recombination and accelerates degradation pathways, ultimately limiting device stability and efficiency. Conversely, PVK-based HTLs suppress nonradiative recombination losses but suffer from inefficient hole injection, electron accumulation in the emissive layer, and limited device lifetime due to severe charge imbalance (*27*). Therefore, developing HTL configurations that simultaneously mitigate surface quenching and enhance hole mobility remains critical for advancing toward high-performance and stable blue PeLEDs.

### Molecular packing and properties of polymer blend HTLs

**Fig. 2.**
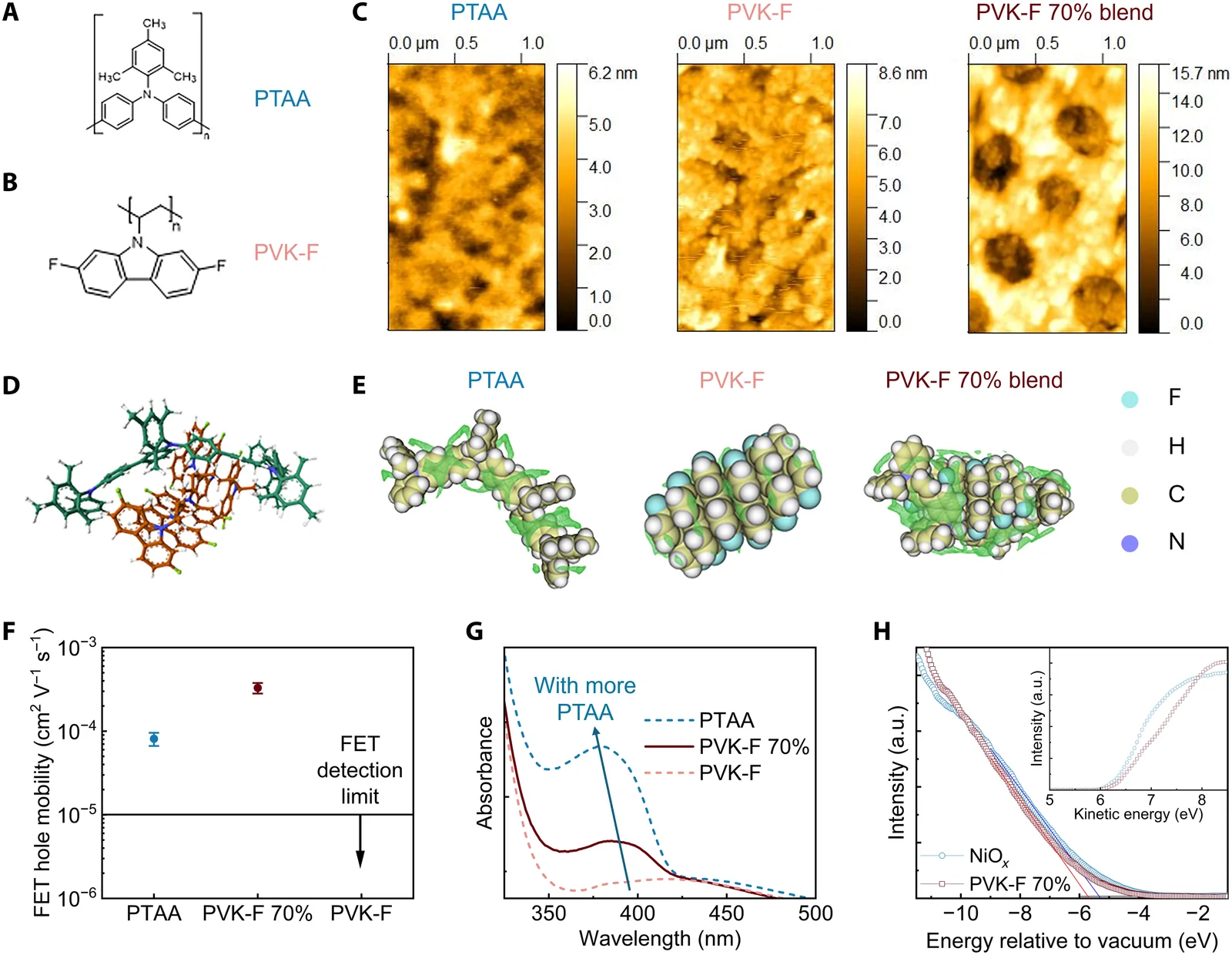
Morphology, molecular packing, and optoelectronic properties of polymer blends. (**A** and **B**) Molecular structures of PTAA and PVK-F, respectively. (**C**) Atomic force microscopy (AFM) images of PTAA, PVK-F, and PVK-F 70% blend (polymer blends with 70% of PVK-F and 30% of PTAA). (**D**) DFT-optimized geometries of PVK-F 70% blend structure. (**E**) Spatial visualization of vdW potentials. All three figures use the same isovalue, shown in green mesh. (**F**) Hole mobility for PTAA, PVK-F, and PVK-F 70% blend, measured by field-effect transistors (FETs) in the saturation regime. (**G**) Absorption spectra for polymer blends with different PTAA and PVK-F ratios. (**H**) UPS spectra of NiO*_x_* and PVK-F 70% blend for HOMO energy level and the corresponding secondary electron edge. a.u., arbitrary units.

PTAA (Fig. 2A) is initially selected as an alternative HTL to NiO*_x_* due to its relatively high hole mobility among polymeric HTLs and a HOMO energy level comparable to that of NiO*_x_* (*38*). Although devices using PTAA/PVK/PVP (denoted as PTAA-based) HTL configuration exhibit improved EQE and peak luminance compared to PVK-based devices (fig. S13), the hydrophobicity of PTAA leads to poor wettability and affects perovskite/HTL interfacial quality, which also reduces reproducibility in device fabrication. These limitations motivate us to modify PTAA to preserve its charge transport advantage while improving the interfacial properties for perovskite deposition. Thus, we blend PTAA with a second polymer because blended organic systems can alter intermolecular packing and surface properties at the interface, thereby promoting favorable film formation (*39*). In this regard, PVK is a possible candidate because it is a widely used carbazole-based HTL and provides a more favorable surface for perovskite film formation than pristine PTAA. To test this idea, we then examine PVK:PTAA blend HTLs. Perovskite films directly spin-coated on pristine PTAA show severe cracking (fig. S14A), whereas films deposited on PVK:PTAA blends exhibit clearly improved surface coverage (fig. S14B), indicating that blending PTAA with PVK improves the wettability of the underlying HTL. Device measurements further support this trend, with PeLEDs based on PVK:PTAA blend/PVK/PVP (denoted as PVK blend-based) HTL configuration exhibit a higher average EQE than devices using PTAA-based HTL configuration (fig. S15), confirming that polymer blending is an effective way to improve the perovskite/HTL interface and device performance.

The success of PVK:PTAA blending motivates us to use PVK-F (Fig. 2B), a fluorinated analog of PVK, to further optimize the blend HTL. Fluorination is a useful molecular design strategy because it can tune the electronic structure, intermolecular packing, and surface properties of polymers (*40*–*42*). These effects make fluorinated carbazole polymer blend HTLs promising candidates for improving both hole transport and perovskite/HTL interfacial qualities. Perovskite precursor solutions are directly spin-coated onto these PVK-F:PTAA polymer blend HTLs without applying intermediate PVK and PVP layers (fig. S16). Notably, perovskite films deposited on these polymer blends containing 50 to 90% PVK-F in weight ratio exhibit markedly improved surface coverage, with optimal film uniformity achieved at 70% of PVK-F and 30% of PTAA (abbreviated as PVK-F 70% blend). In contrast to perovskite films deposited on pure PTAA and PVK-F, which display pronounced pinholes and cracks, the optimized PVK-F 70% blend yields compact and continuous perovskite layers, indicating enhanced wettability of this blended HTL. This improvement in wettability is further supported by contact-angle measurements, which reveal a reduced contact angle of 54.6° for the PVK-F 70% blend, compared with 61.4° and 62.1° for pristine PTAA and PVK-F films, respectively (fig. S17).

Atomic force microscopy (AFM) reveals distinct morphological differences among pure PTAA, pure PVK-F, and PVK-F 70% blend. Specifically, the pure PTAA and PVK-F exhibit typical continuous polymer morphologies, while the optimized PVK-F 70% blend features regular pores ∼400 nm in width and 8 nm in depth (Fig. 2C and fig. S18). Systematic AFM analysis of blends with varying PTAA and PVK-F ratios (figs. S19 and S20) further demonstrates the tunability of blend polymer morphology, highlighting the critical role of intermolecular interactions in governing surface structures (*39*). Collectively, these results identify PVK-F:PTAA polymer blends as promising HTLs for blue PeLEDs, offering improved perovskite film coverage and enhanced device performance and reproducibility.

To elucidate the origin of the improved wettability and unique porous morphology observed in the optimized polymer blend (PVK-F 70% blend), density functional calculations are performed to examine the molecular packing of PTAA and PVK-F individually, as well as their interactions in the PVK-F 70% blend. The calculations indicate strong intermolecular π-π stacking between the fluorocarbazoles in PVK-F molecules, promoting a compact and rigid molecular structure. By contrast, PTAA molecules exhibit higher flexibility and more extended conformations. Figure 2D shows the optimized lowest energy geometry of the interacting PVK-F 70% blend system, illustrating strong π-π stacking interactions within PVK-F and flexible PTAA chains “wrapping” around PVK-F molecules, promoting dense molecular packing. This rigidity mismatch between PVK-F and PTAA is likely responsible for the observed porous morphology. When a rigid polymer is blended with a more flexible counterpart, rapid solvent evaporation during spin coating can trigger spontaneous rigidity-driven microphase separation, producing porous or depression-like surface morphologies, even without a dissolution step (*43*, *44*). Such self-generated porous morphology can enhance wettability via the Wenzel wetting mechanism (*45*), where partial liquid penetration into the pores increases the effective solid-liquid contact area and reduces the measured contact angle (fig. S17). In addition, despite relatively small changes in the contact angle across blend ratios, the pronounced morphological evolution suggests that wettability is strongly influenced by surface chemistry. Polymer blending may induce surface rearrangement or partial phase segregation, thereby modifying surface molecular composition, orientation, and intermolecular packing, which together promote more favorable interactions with water.

To further correlate the molecular packing and wettability changes, we evaluate the molecular interactions in PTAA, PVK-F, and PVK-F 70% blend by calculating the spatial van der Waals (vdW) potential due to their dominant effect over electrostatic interactions for wettability (*46*). As shown in Fig. 2E, individual PTAA and PVK-F polymers exhibit the lowest vdW potentials of −1.057 and −1.055 kcal mol^−1^, respectively, whereas the PVK-F 70% blend system shows a lowered potential of −1.555 kcal mol^−1^. This substantial reduction indicates enhanced intermolecular interactions not only between PVK-F and PTAA molecules but also with other molecules deposited on the blend surface. These calculations thus suggest that the inclusion of PVK-F induces a more compact arrangement of the otherwise loosely packed PTAA chains, thereby leading to the observed improved wettability in the PVK-F:PTAA blends (figs. S16 and S17).

Following the confirmation of the dense molecular packing in the polymer blends, we then investigate their impact on charge-transport properties. We measure the field-effect hole mobility for polymers by constructing bottom-gate, bottom-contact field-effect transistors (FETs) (more details in Materials and Methods). As shown in Fig. 2F, the pure PTAA exhibits a hole mobility of 9.5 × 10^−5^ cm^2^ V^−1^ s^−1^, which is comparable to previously reported values (*38*). However, no measurable field-effect current can be detected for the pure PVK-F (fig. S21E), indicating that its hole mobility is below the detection threshold of ∼10^−5^ cm^2^ V^−1^ s^−1^. Notably, the optimized PVK-F 70% blend exhibits an enhanced hole mobility of 3.3 × 10^−4^ cm^2^ V^−1^ s^−1^, consistent with the improved molecular packing from the calculation (Fig. 2D).

To explore whether new energetic states are formed within the polymer blend due to the enhanced intermolecular interactions between PTAA and PVK-F, we perform ultraviolet-visible absorption (UV-Vis) spectroscopy (Fig. 2G) and PL measurements (fig. S22A) for PVK-F:PTAA blends with varying ratios of PVK-F. As the content of PVK-F in the blend increases, a decrease in absorbance is observed at 350 to 400 nm, attributed to the reduction of PTAA content in the blend, with no additional absorption peaks detected. This result aligns with PL measurements, where no additional emission peaks are observed, suggesting the absence of new energetic states in the blend. Specifically, broad spectra are found for both pure PTAA and PVK-F, with emission peaks at 420 and 390 nm, respectively. In the PVK-F:PTAA polymer blends, both peaks are observed with a more prominent signal at 420 nm upon the addition of PVK-F (fig. S22B), which can be attributed to energy transfer from PVK-F to PTAA. In addition, ultraviolet photoelectron spectroscopy (UPS) analysis (Fig. 2H) reveals a deeper HOMO energy level of −5.7 eV for PVK-F 70% blend, 0.23 eV deeper than that of NiO*_x_* (−5.47 eV), which is favorable for efficient hole injection into PVK and the perovskite emissive layer during device operation. Overall, these results demonstrate the successful design of an optimized polymer blend HTL with enhanced wettability, molecular packing, and hole mobility over individual polymer or inorganic HTLs, substantially improving perovskite film quality and providing a viable approach to achieving high-performance and stable blue PeLEDs.

### Optical properties for perovskites deposited on blend HTLs

**Fig. 3.**
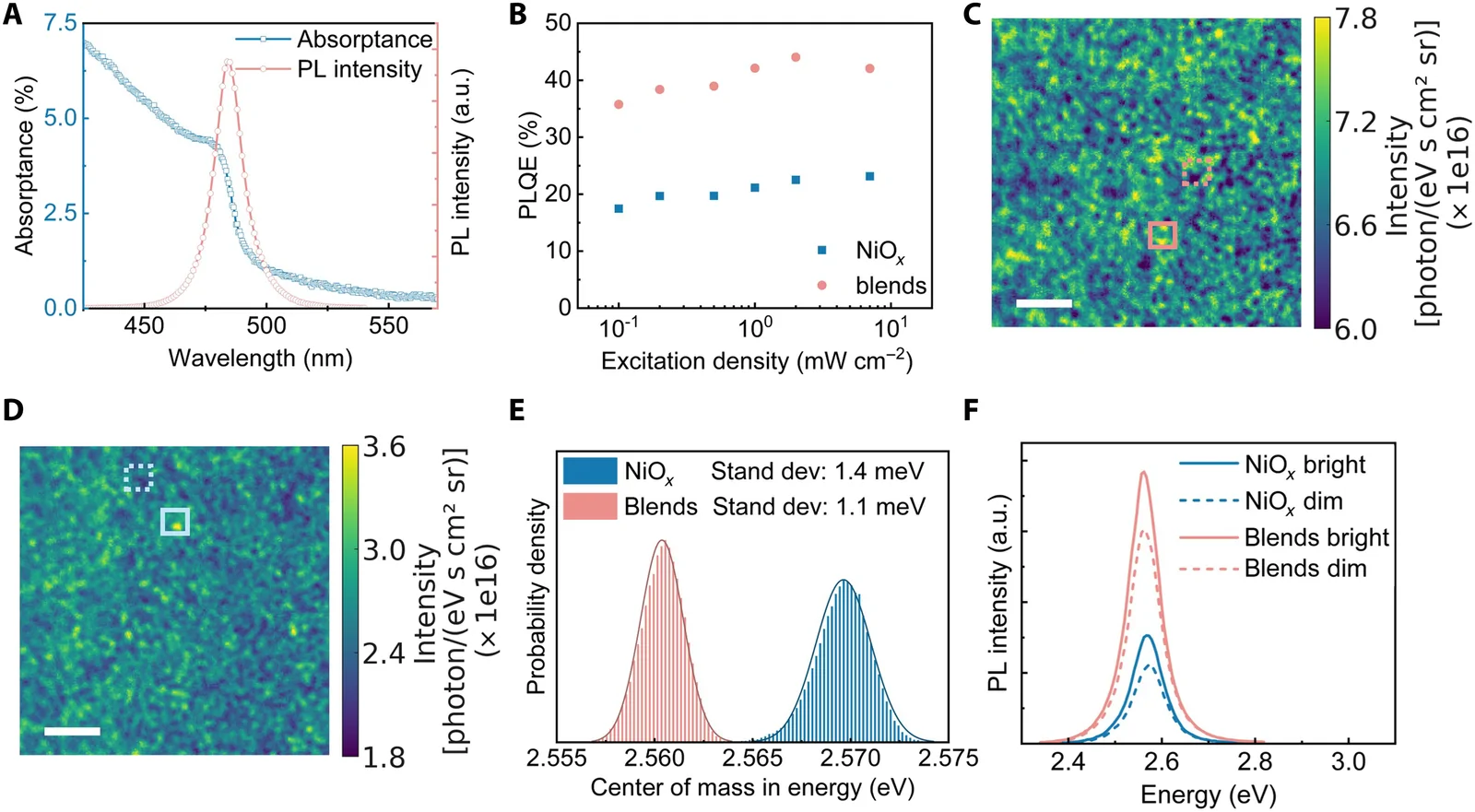
Luminescent properties of perovskite emitters on a blend-based HTL configuration. (**A**) Absorption and PL spectra for perovskites spin-coated on blend (PVK-F 70% blend)/PVK/PVP (denoted as blend-based) HTL configuration. (**B**) Fluence-dependent PLQE for perovskites prepared on blend- and NiO*_x_*-based HTL configurations. (**C** and **D**) Hyperspectral PL intensity maps for perovskites prepared on blend- and NiO*_x_*-based HTL configurations at their respective PL peak wavelength of 484 nm and 483 nm, respectively. Scale bars, 4 μm. (**E**) Local energy distribution extracted from COM maps for perovskites on blend- and NiO*_x_*-based HTL configurations, each histogram bin has a width of 1 meV. (**F**) Corresponding local PL spectra from bright and dim regions from both HTL configurations.

After demonstrating the improvement of wettability and hole mobility for the PVK-F 70% blend HTL, we examine the optical properties of perovskite films deposited onto this optimized HTL configuration. As shown in Fig. 3A, perovskite films deposited on blend (PVK-F 70% blend)/PVK/PVP (denoted as blend-based) HTL relevant for the device configuration exhibit an absorption edge at ∼480 nm and a PL peak at 484 nm, similar to films on NiO*_x_* (fig. S23A). Furthermore, the PLQE of perovskite films deposited on the blend-based HTL configuration is twice as high as that on the NiO*_x_*-based counterpart (Fig. 3B), demonstrating that carrier quenching is better suppressed by replacing NiO*_x_* with the PVK-F 70% blend. To quantitatively assess the emission homogeneity, we perform hyperspectral PL mapping for perovskite films prepared on both blend-based and NiO*_x_*-based HTL configurations. Figure 3 (C and D) displays hyperspectral PL maps extracted at the emission peak wavelengths of 484 and 483 nm for perovskites on blend and NiO*_x_*, respectively. Both maps reveal isolated island morphology, which is typical for PeLEDs (*15*), consistent with the morphology observed from scanning electron microscopy (SEM) in figs. S1B and S24 and AFM in fig. S25. Notably, the photon flux from the perovskite deposited on blend-based HTL configurations is more than twice that from NiO*_x_*-based configuration, aligning with the observed enhancement in PLQE (Fig. 3B). Furthermore, statistical analyses of the emission peak energy distribution from center-of-mass (COM) hyperspectral mapping indicate a more uniform emission profile for blend-based films (fig. S26). Specifically, center wavelengths correspond to dominant energies at 2.56 eV (484 nm) for blend-based films, while slightly blue-shifted peaks (2.57 eV, 482 nm) are observed in NiO*_x_*-based films (Fig. 3E). Statistical fitting of the COM energy distributions yields a standard deviation of 1.1 meV for the blend-based HTL, compared to 1.4 meV for the NiO*_x_*-based HTL, confirming improved emission homogeneity in the blend-based films. We further examine nanoscale heterogeneity by comparing local PL spectra from both bright and dim regions (Fig. 3F) in both HTL configurations [highlighted in Fig. 3 (C and D)]. Perovskite films deposited on blend-based HTL show minimal spectral shifts between bright and dim regions, while a difference of 5 meV is found for the NiO*_x_*-based HTLs. Overall, these results collectively demonstrate that the blend-based HTL not only improves the PLQE of the emitter but also yields more spatially uniform emission, underscoring its potential for enhancing PeLED color purity and performance.

### Device performance and stability enabled by polymer blend HTLs

To evaluate the impact of the optimized blend HTL design on the PeLED performance, we first assess whether the porous morphology of PVK-F 70% blend HTL induces current leakage. Devices are fabricated with the structure ITO/PVK-F 70% blend/PVK/PVP/perovskite (with or without)/TPBi/LiF/Al. As shown in fig. S27, devices fabricated with the porous PVK-F 70% blend/PVK/PVP (blend-based) HTL but without the perovskite layer exhibit lower current densities at low bias compared to the devices with perovskites. No ohmic-like conduction is observed, indicating that the porous HTL does not introduce substantial shunting or leakage pathways under forward bias. Despite its porous morphology, the blend-based HTL maintains complete coverage on the ITO substrate, thereby preventing the formation of continuous pinholes or percolation pathways that could directly connect the bottom and top electrodes, which are the primary causes of current leakage in LEDs (*47*, *48*).

Subsequently, the performance of PeLEDs with architecture ITO/PVK-F 70% blend/PVK/PVP/perovskite/TPBi/LiF/Al is evaluated. These devices exhibit an intense EL emission peak at 485 nm and deliver a champion EQE of 17% (fig. S28), demonstrating an average peak EQE of 15.4% (fig. S29). This performance represents a substantial improvement compared with average EQEs for NiO*_x_*-based, PVK-based, and PTAA-based PeLEDs (figs. S29 and S15). The operational lifetime (*T*_50_) for blend-based PeLEDs reaches a *T*_50_ of 190 min at an initial luminance of 100 cd m^−2^ (fig. S30) compared to 8 min for PVK-based PeLEDs. We see similar trend with improvement in operational lifetime in the deep-blue devices after applying this blend-based HTL configuration (fig. S31). This enhanced operational lifetime can be attributed to improved charge balance at the perovskite/HTL interface. In blue PeLEDs, insufficient hole injection leads to electron accumulation near the interface, which can trigger interfacial redox reactions and defect formation, progressively increasing nonradiative recombination and series resistance (*16*, *21*). By providing improved hole mobility (Fig. 2F) and favorable energy-level alignment (fig. S32), the polymer-blend HTL suppresses electron accumulation, thereby mitigating charge-induced interfacial degradation and slowing voltage rise under operation. This balanced injection is therefore central to the extended device lifetime observed.

To confirm the improvement of hole injection characteristics, we test hole-only devices with a structure of ITO/PVK-F 70% blend (with or without)/PVK/PVP/perovskite/TFB/MoO*_x_*/Au. As shown in fig. S33A, the current density measured for the blend-based hole-only devices exceeds that of PVK-based hole-only devices, confirming an enhanced hole injection with the blended HTL. The facilitated hole injection contributes to a reduction in the current density required to achieve 100 cd m^−2^ of luminance, thus alleviating the overall electrical stress across the PeLEDs. Given that the degradation of p-i-n structured blue LEDs also stems from the interface between HTL and the emissive layer (*16*), which leads to an increase in resistance over time and manifests as a voltage rise under constant current operation, we evaluate the stability of the hole-only devices. In constant current density measurements (50 mA cm^−2^), blend-based hole-only devices exhibit a notably lower initial operating voltage (0.84 V) compared to PVK-based devices (1.20 V), accompanied by a smaller voltage increase ratio after 2 hours of operation (5.2% versus 18%; fig. S34, A and B). Stability measurements conducted on multiple hole-only devices consistently show that blend-based hole-only devices have an average voltage increase of only 7%, compared to 17% for PVK-based counterparts after continuous operation for 2 hours (fig. S34C). The lower initial voltage stress under the same operational bias and a slower increase in series resistance in blend-based devices indicate reduced degradation at the HTL/perovskite interface, thereby substantially enhancing device operational lifetime. To further understand the origin of the improved device stability, we examine electrically stressed PeLEDs by cross-sectional high-angle annular dark-field scanning transmission electron microscopy (HAADF-STEM). In the PVK-based device after 35 min of biasing at a constant current density of 1 mA cm^−2^, distinct bright inclusions are observed near the HTL/perovskite interface, particularly around the isolated perovskite islands (fig. S35A). These features indicate the formation of lead-rich phases, which are assigned to degraded products such as metallic lead or PbX_2_ produced by decomposition of the perovskite structure during operation. In contrast, the blend-based device under the same bias condition shows a marked reduction in the density of these bright inclusions after the same electrical stress (fig. S35B), indicating suppressed formation of lead-rich degraded phases. Together with the improved hole injection and smaller voltage rise observed in hole-only devices, these results support the conclusion that the blend-based HTL configuration mitigates interfacial reactions, thereby improving operational stability.

It is important to note that different perovskite compositions exhibit distinct crystallization behaviors onto the same HTL configuration, which substantially affect device performance (*12*, *22*, *49*). Therefore, it remains essential to evaluate whether the optimized PVK-F 70% blend HTL can be generalized effectively across various perovskite compositions to consistently enhance device performance. For this, we use a different perovskite composition with a previously reported molecular additive, 4-fluorobenzenesulfonamide (FBA; molecular structure shown in fig. S36) (*50*). The sulfonyl group [-S(=O)_2_-], combined with the conjugated aromatic ring in FBA, allows the electrons to be delocalized among the whole molecule, which binds with perovskite surface uncoordinated Pb^2+^ and effectively passivates trap density, contributing to an improved luminescent efficiency and perovskite stability compared to the perovskite without FBA (*50*).

**Fig. 4.**
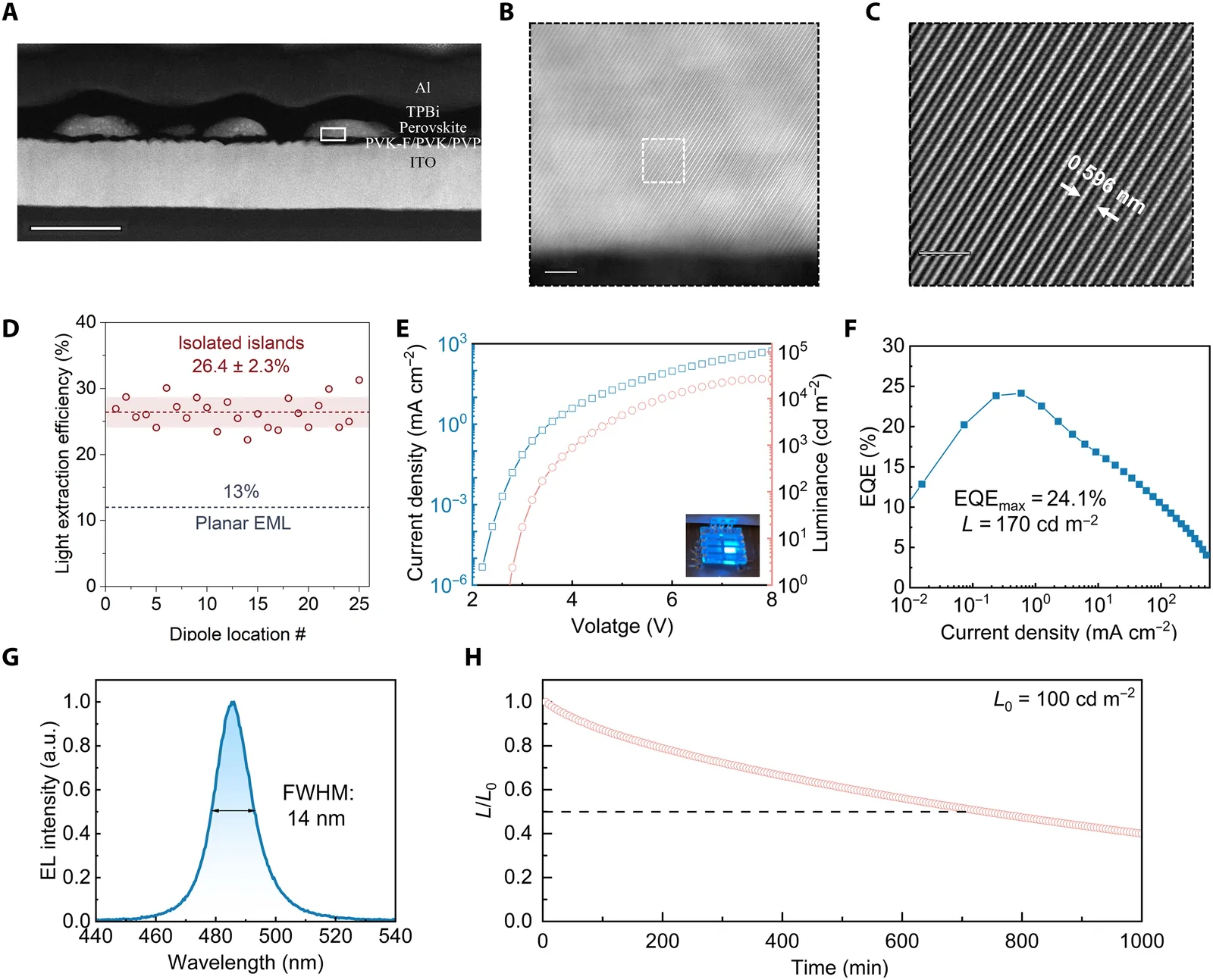
Performance and operational lifetime of blue PeLEDs using FBA-modified perovskite emitters and PVK-F 70% blend HTLs. (**A**) Cross-sectional scanning transmission electron microscopy of the PeLEDs. Scale bar, 200 nm. (**B**) Atomic resolution image at perovskite/HTL interface. Scale bar, 5 nm. (**C**) Atomic resolution image in a perovskite island. Scale bar, 2 nm. (**D**) Simulated light extraction efficiencies for perovskites prepared on PVK-F 70% blend/PVK/PVP as a function of grain length or period (fixed duty cycle: 50%). (**E**) Current density-voltage-luminance characteristics of the champion device based on ITO/PVK-F 70% blend/PVK/PVP HTL configuration. (**F**) EQE of PeLEDs based on ITO/PVK-F 70% blend/PVK/PVP configuration. (**G**) EL spectrum of the device. (**H**) Operational stability of PeLED under an initial luminance of 100 cd m^−2^.

By incorporating FBA into our 3D perovskite, the PLQE of the FBA-incorporated emitters is boosted above 60% (fig. S37). PeLEDs incorporating FBA-modified perovskite combined with our blend-based HTL configuration are then fabricated. The HAADF-STEM image shown in Fig. 4A reveals an elliptical cross section of island-shaped perovskite, with a height of 40 to 50 nm for each perovskite island. A HAADF-STEM image acquired at the HTL/perovskite interface (Fig. 4B) indicates a preferred crystal growth orientation along the [100] direction. In the zoomed HAADF-STEM image of the bulk perovskite island (Fig. 4C), the bright spots are Pb ions while the dark spots are A-site atom (Cs or FA). The d-spacing is 0.596 nm, corresponding to the (110) plane. These perovskite films, exhibiting isolated island morphology on the blend-based HTL configuration (fig. S38A), enabled an EL photon outcoupling efficiency of ∼26% in fabricated LEDs (Fig. 4D). This represents an improvement compared to the 21% EL outcoupling efficiency observed with the NiO*_x_*-based HTL configuration [fig. S38D; see details of the 3D finite-difference time-domain (3D-FDTD) simulation of the light extraction processes in fig. S38]. Owing to its ultrathin thickness and burial beneath the emissive layer, the porous PVK-F 70% blend HTL is not expected to substantially influence the outcoupling efficiency, which is instead dominated by the island perovskite morphology and the overall device architecture.

PeLEDs incorporating FBA-modified perovskite emitters exhibit an EL peak at 485 nm with a narrow FWHM of 14 nm, demonstrating excellent spectral purity (Fig. 4G). Consistent with the improved hole injection observed in FBA-modified perovskite hole-only devices using the PVK-F 70% blend as the underneath HTL (fig. S33B), the champion PeLED presents a peak EQE of 24.1% at a luminance of 170 cd m^−2^ (Fig. 4, E and F). The average peak EQE is 22.1%, which is substantially higher relative to the average EQE of 15.6% with control perovskite (fig. S39). The PLQE of 60% (fig. S37) for FBA-modified perovskite films is consistent with the measured peak device EQE of 24.1% after considering optical outcoupling losses in the PLQE measurement geometry (*51*). The observed EQE roll-off at higher current densities is consistent with high carrier density-induced nonradiative loss processes commonly reported in high-efficiency blue PeLEDs (*10*–*12*), which are distinct from the interfacial charge injection limitations addressed in this work. Benefiting from improved PLQE (fig. S37) and hole injection, the devices achieve a maximum luminance of 26,000 cd m^−2^, and an operational lifetime *T*_50_ of 735 min at an initial luminance of 100 cd m^−2^ (Fig. 4H; fig. S40 for stability measurements at initial luminances of 1000 and 10,000 cd m^−2^), accompanied by a minor EL redshift of 0.8 nm after 700 min of continuous bias (fig. S41). This operational lifetime substantially exceeds that of devices incorporating FBA-modified emissive layers fabricated on PVK-based HTLs, which exhibit a *T*_50_ of 40 min (fig. S42), thereby confirming the critical role of the polymer-blend HTL in enhancing device operational stability.

We further evaluate the generality of the blend-based HTL by using a quasi-2D perovskite emissive layer in devices with the same architecture used in Fig. 4A. As shown in fig. S43 (A and B), the current density-voltage-luminance characteristics reveal that quasi-2D perovskites deposited on the blend-based HTL achieve higher luminance compared with those on the PVK-based HTL, accompanied by enhanced EQEs (fig. S43C). Statistical analysis of nine devices further confirms this trend, with the blend-based devices exhibiting a higher average EQE of 3.8%, compared with 2.0% for the PVK-based devices (fig. S43D). These findings demonstrate the potential of polymer blends with enhanced hole mobility to improve the operational lifetime and performance of solution-processed PeLEDs. Further development of advanced polymer blend designs is essential for advancing the field of high-performance and stable LEDs.

## DISCUSSION

In summary, our work reveals that the primary limitation in the performance of blue PeLEDs based on bulk 3D perovskite films is related to hole transport layers and their interfaces. We design a polymer blend HTL based on PTAA and PVK-F with improved mobility and wettability due to the enhanced intermolecular interactions induced by fluorinated groups. This blend enables an improvement in hole injection and therefore demonstrates high-efficiency, stable blue PeLEDs with a peak EQE of 24.1%, *T*_50_ of 735 min, and a peak luminance of 26,000 cd m^−2^. Our work provides guidelines and stimulates further efforts in designing HTLs with improved hole mobility and wettability, as well as a deeper HOMO energy level, to boost the efficiency and operational lifetime of PeLEDs, particularly for applications requiring deep blue emission.

## MATERIALS AND METHODS

### Materials

CsBr (99.999%), PbBr_2_ (99.999%), PbCl_2_ (99.999%), 4-fluoro-phenethylammonium bromide (p-f-PEABr), lithium bromide (LiBr), zinc chloride (ZnCl_2_), bis(triphenylphosphine)iminium chloride (PPNCl), poly(4-vinylpyridine) (PVP; Weight-average molecular weight, ∼60,000), TTDDA (97%), FBA, PVK (Number-average molecular weight, 25,000 to 50,000), LiF (99.99%), DMSO (99.9%), DMF (99.8%), chlorobenzene (CB; 99.8%), and 2-propanol (IPA; 99.5%) were purchased from Sigma-Aldrich. FABr was purchased from Greatcell Solar Materials. TPBi and PTAA were purchased from Ossila. PVK-F was purchased from Lumitech. The NiO*_x_* nanocrystals were purchased from Avantama AG and were used without additional treatment.

### Preparation of the perovskite precursor solution

Control 3D perovskite precursor solutions (CsBr:FABr:PbBr_2_:PbCl_2_:TTDDA = 1.2:0.3:0.3875:0.6125:0.1) were dissolved in DMSO with the Pb concentration of 0.1 M. For FBA-incorporated perovskite precursor solutions, an extra 10% of FBA in molar ratio to Pb was added into the control 3D perovskite precursor solution. Both control 3D and FBA-incorporated perovskite precursor solutions were stirred at 65°C before spin coating. For the quasi-2D mixed-halide composition, p-f-PEABr:CsBr:FABr:LiBr:ZrCl_2_:PbBr_2_:PbCl_2_:PPNCl = 0.8:1:0.05:0.3:0.05:0.52:0.52:0.056 were dissolved in DMSO at a Pb concentration of 0.1 M for sky-blue EL emission, which is a standard sky-blue composition reported before (*13*).

### PeLED fabrication

ITO substrates were cleaned with deionized water, acetone, and isopropanol for 15 min, followed by ultraviolet ozone treatment for 20 min, and then transferred into a nitrogen-filled glove box. NiO*_x_* was deposited in atmosphere with a spin coating rate of 3000 rpm for 30 s, followed by annealing at 120°C for 10 min. Polymer blend containing PTAA and PVK-F with a ratio of 3:7 (5 mg ml^−1^ in CB) was deposited on the ITO substrates and annealed at 120°C for 10 min. Then, PVK (9 mg ml^−1^ in CB) was spin-coated onto the polymer blend, followed by annealing at 100°C for 10 min. Then, a thin layer of PVP (1.5 mg ml^−1^ in IPA) was deposited and annealed at 80°C for 10 min. All the above-mentioned HTLs, they were spin-coated at a speed of 3000 rpm for 30 s. Last, either control 3D or FBA-incorporated perovskite precursor was spin-coated at 4000 rpm for 40 s. Right after spin coating, the films were transferred into an enclosed petri dish with 20 μl of DMF, following the previously reported procedure (*15*). After DMF treatment for a certain time, the perovskite films were annealed at 80°C for 8 min. The quasi-2D mixed halide composition, it was spin-coated at 4500 rpm for 60 s and annealed at 80°C for 5 min, without any vapor treatment applied after the spin coating. Then, all the substrates were loaded into the evaporator with a pressure of 10^−6^ mbar for evaporation. 50-nm TPBi, 1.5-nm LiF, and 100-nm Al were subsequently deposited with shadow masks via thermal evaporation. The active area of LEDs is 4.5 mm^2^.

### PeLED characterization

PeLED device *J*-*V*-*L* measurements were carried out in atmosphere with encapsulation of active area. The voltage-current density-luminance characteristics were measured using a Keithley 2400 source meter and a calibrated silicon photodetector. The LED metrics were calculated considering the responsivity of the silicon photodetector. The EL spectra were obtained using an Ocean Optics Flame spectrometer.

### FET fabrication and measurement for polymers

SiO_2_ (300-nm SiO_2_ on n++ silicon) substrate was prepatterned with FET channel patterns via photolithography. Metal electrodes were deposited by thermal evaporation in high vacuum (<2 × 10^−6^ mbar). Cr (4 nm) at an evaporation rate of 0.5 Å s^−1^ was deposited to serve as an adhesion layer between SiO_2_ and gold. Polymers were then spin-coated on the FET substrates with the same method as in PeLED fabrication. FET channel length and width are 0.1 mm and 1 mm. FET devices were measured in low vacuum (1 × 10^−3^ mbar). Saturation regime transfer characteristics were measured at *V*_DS_ = −60 V and *V*_G_ sweeping between +20 and −60 V at 1-V step.

### Grazing-incidence wide-angle x-ray scattering

GIWAXS was performed on beamline I07 at Diamond Light Source in Didcot, United Kingdom. The beam energy for x-ray was 10 keV, and a Pilatus 2 M large area detector placed at a sample-detector distance of 0.55 m records the scattering. Films were measured with a pitch angle of 0.20°.

### Steady-state PL

PL was measured by using Edinburgh Instruments FLS1000 with an excitation wavelength of 405 nm. The spectrum was captured by a step of 1 nm and an exposure time of 0.2 s at each wavelength. For photogenerated current density calculations in PL stability tests, the absorptance and EQE of the device are considered, by using the formula of $J=\frac{e\times \text{EQE}\times A\times P\times \lambda }{hc}$, where *J* is the current density, and *e* is the elementary charge, EQE is external quantum effiency, *A* is the absorptance for perovskite at 405 nm, *P* is the power density of the laser, λ is the laser wavelength, *h* is the Planck’s constant, and *c* is the speed of light.

### Hyperspectral PL

Hyperspectral microscopy measurements were conducted using a Photon etc. IMA system. Olympus numerical aperture = 0.9 objective lens, with ×63 magnification was used for all measurements. To correct for aberrations stemming from the optical elements in the system, the apparatus was calibrated using a reference sample to determine the postprocessing parameters that will be used to correct the image distortion. Chromatic aberrations were mitigated by calibrating the *z* position of the sample for every collected wavelength. A 405-nm continuous wave laser served as the excitation source for the PL. The light emitted from the sample was incident on a volume Bragg grating, which spectrally split the light onto a silicon Hamamatsu complementary metal-oxide semiconductor camera with 2048 × 2048 pixel array.

### Ultraviolet-visible spectroscopy

The UV-Vis transmittance measurement for the samples was performed on an Agilent Cary 7000 Universal Measurement Spectrometer. A polytetrafluoroethylene (PTFE) plate placed at the reflectance port was used as the baseline reference (100% transmittance and reflectance) for all measurements. The samples were measured across a wavelength range of 300 to 800 nm with an integration time of 0.2 s, slit bandwidth of 2 nm, double beam mode, reduced slit height, source changeover of 320 nm, detector changeover of 800 nm, and grating changeover of 800 nm, respectively.

### Atomic force microscopy

AFM images were obtained on an Asylum Research MFP-3D in air, using alternating current mode and a Bruker silicon cantilever (spring constant, 48 N m^−1^).

### Scanning electron microscopy

SEM images were collected on a Zeiss Gemini using the Inlens detector, using an acceleration voltage of 3.0 kV and an aperture size of 30 μm for morphological information.

### Ultraviolet photoelectron spectroscopy

UPS was measured on Thermo Fisher Scientific Escalab 250Xi with a Helium gas discharge lamp with an energy of 21.22 eV. The setup has been calibrated by Ag. Measurement was conducted under ultrahigh vacuum conditions at 10^−9^ torr. For HOMO energy analysis, linear extrapolation method is applied. The first derivative of the intensity with respect to the binding energy is calculated to quantify the rate of change in intensity. The steepest slope corresponds to the maximum value of the derivative (for the first derivative reaches its maximum). Data points were selected around the steepest slope as the linear region, and the line was extrapolated to find out the intersection with the extrapolation from the baseline. The energy at intersection point corresponds to HOMO energy.

### Scanning transmission electron microscopy–energy dispersive x-ray spectroscopy

The TEM lamella cross section was prepared using an FEI Helios Nanolab Dualbeam focused ion beam (FIB)/SEM following the standard protocols. The lamella was transferred with minimal air exposure into a Spectra 300 TEM operating at 300 kV and ∼130-pA beam current. HAADF images were captured using a Fischione detector with a camera length of 115 mm, a dwell time of 1 μs, and a spatial sampling of 1 nm per pixel for high-resolution data acquisition. Scanning transmission electron microscope-energy dispersive x-ray spectroscopy (STEM-EDX) maps were acquired using four Super-X detectors with a dwell time of 0.5 ms per scanning frame, spatial sampling of 2 nm per pixel, and a spectral resolution of 10 eV per channel. In addition, STEM-EDX spectrum images were obtained with a beam current of 50 pA, using 40 frames, a convergence angle of 24 mrad, and a camera length of 91 mm. The STEM-EDX data were denoised using principal components analysis and processed using HyperSpy. Atomic resolution images were obtained with a beam current of 5 pA, a convergence angle of 17.1 mrad, and 10 frames to average the periodic information to minimize the electron beam damage.

### Spatial vdW potential calculation

The vdW potential was computed using the method developed by Lu and Chen (*52*), in which the vdW interaction is represented by a Lennard-Jones–type potential consisting of an exchange-repulsive term and a dispersion-attractive term, with parameters taken from the Universal Force Field (UFF). A He atom was used as a scanning probe to evaluate the potential on the DFT optimized structure at the CAM-B3LYP/6-31G(d,p) level with Grimme’s D3 dispersion correction (Becke-Johnson damping) (*53*).

## Supplementary Materials

### The PDF file includes:

Figs. S1 to S43 Table S1 Legends for movies S1 to S4 References
