# Supplementary material for: Toward high-performance and stable blue perovskite light-emitting diodes via optimized fluorinated polymer blend hole transport layers
Source: Sci Adv. 2026 Aug 28;12(35):eaec7056. doi: 10.1126/sciadv.aec7056 (PMC13524041; doi:10.1126/sciadv.aec7056)
Supplement: Supplementary file 1 [file sciadv.aec7056_sm.pdf]

Science Advances
AAAS

Supplementary Materials for

Toward high-performance and stable blue perovskite light-emitting diodes via optimized fluorinated polymer blend hole transport layers

Qichun Gu *et al.*

Corresponding author: Tianjun Liu, tl586@cam.ac.uk; Samuel D. Stranks, sds65@cam.ac.uk; Miguel Anaya, anaya@us.es

Sci. Adv. **12**, eaec7056 (2026)
DOI: 10.1126/sciadv.aec7056

The PDF file includes:

Figs. S1 to S43
Table S1
Legends for movies S1 to S4
References

Other Supplementary Material for this manuscript includes the following:

Movies S1 to S4

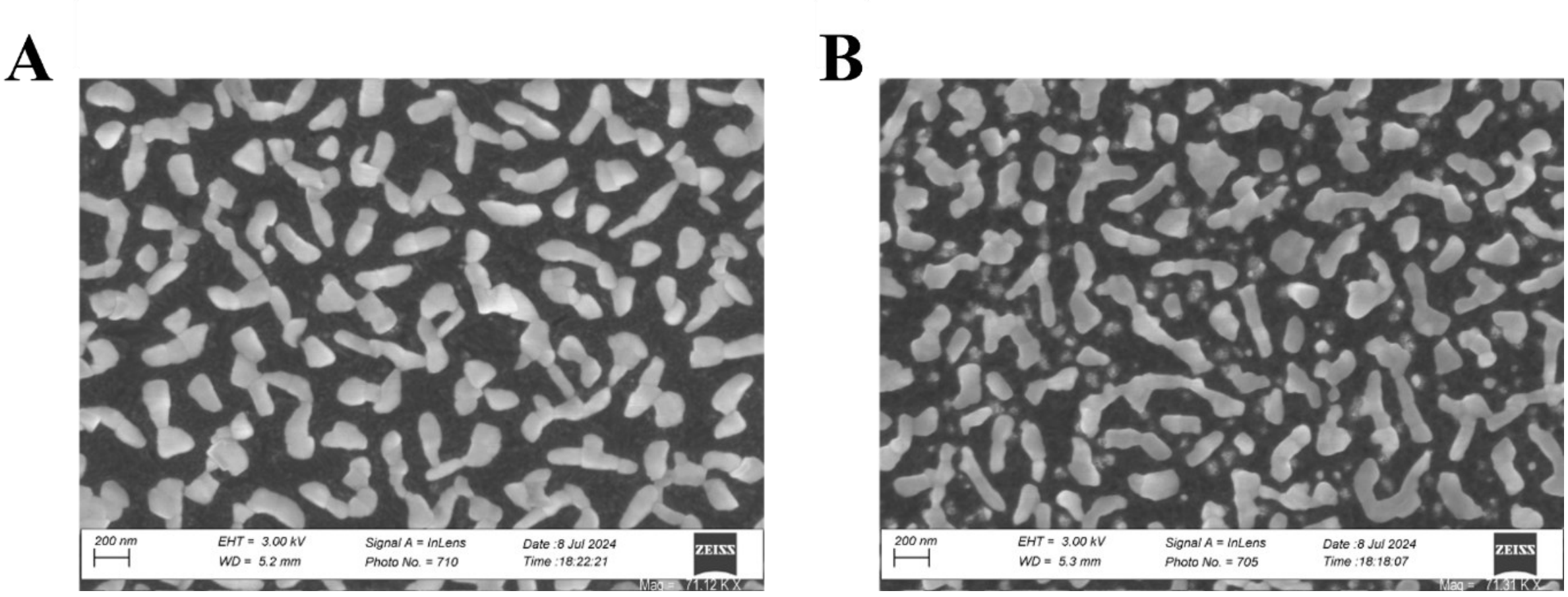

Fig. S1: SEM images of perovskites spin-coated on different HTL configurations. (A), PVK/PVP, (B), NiO_x/PVK/PVP HTL configurations.

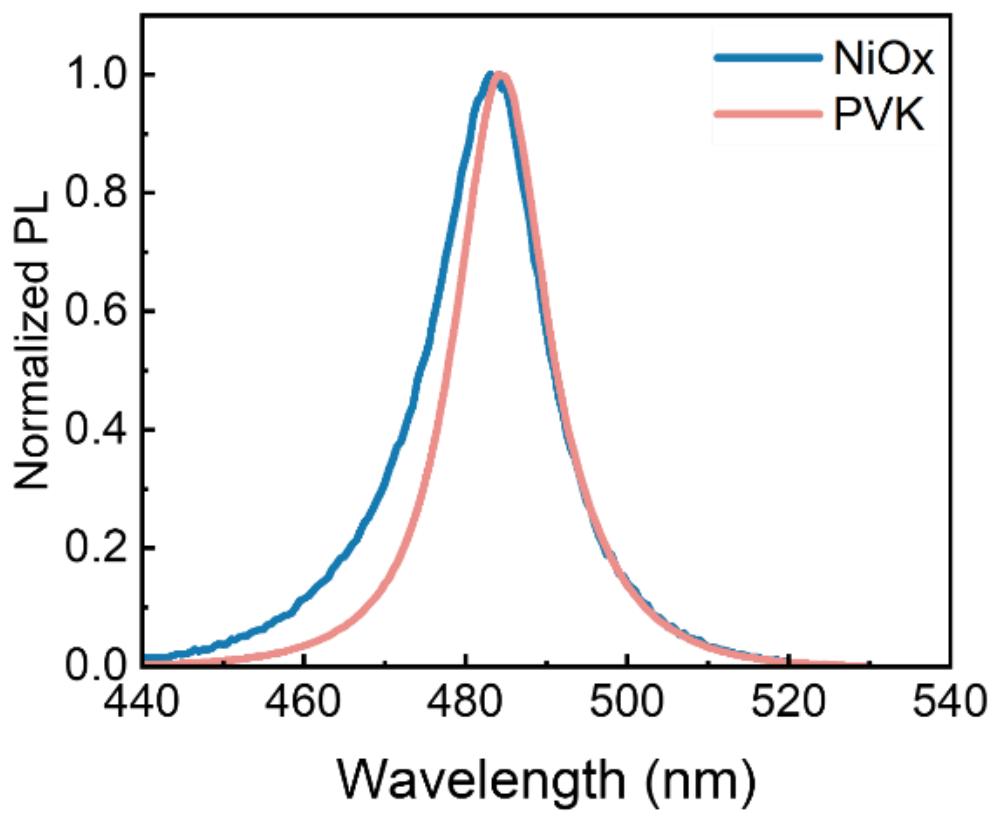

Fig. S2: PL spectra for perovskites deposited on the NiO_x/PVK/PVP and PVK/PVP HTL configurations.

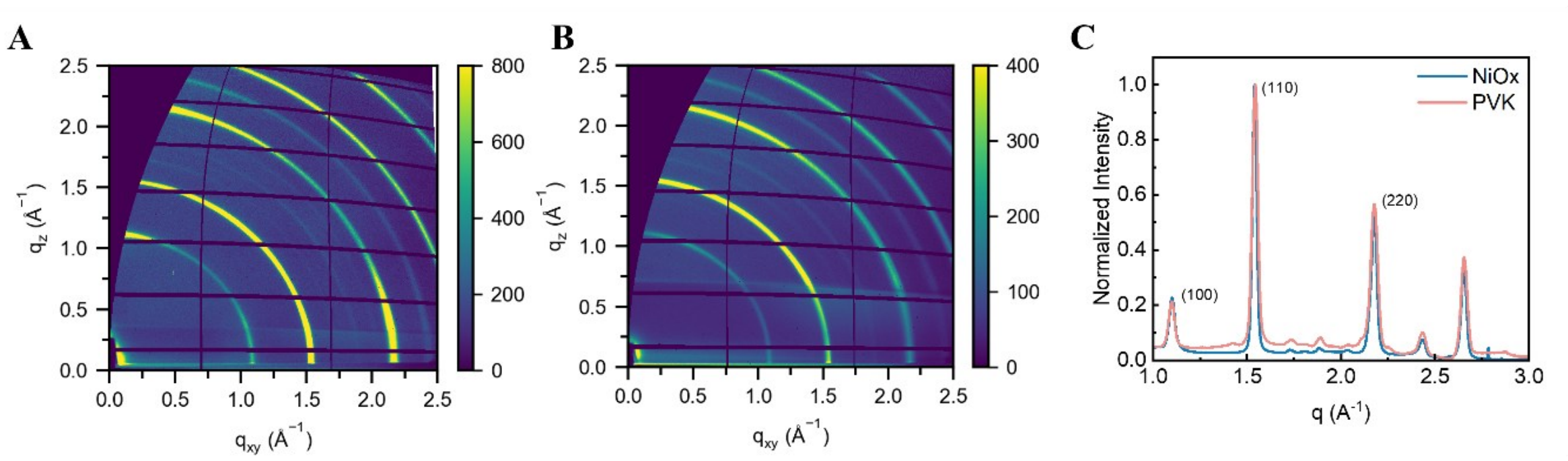

Fig. S3: GIWAXS patterns for perovskites deposited on different HTL configurations. (A), NiO_x/PVK/PVP and (B), PVK/PVP, (C), The corresponding integrated 1D XRD for perovskites on both HTL configurations.

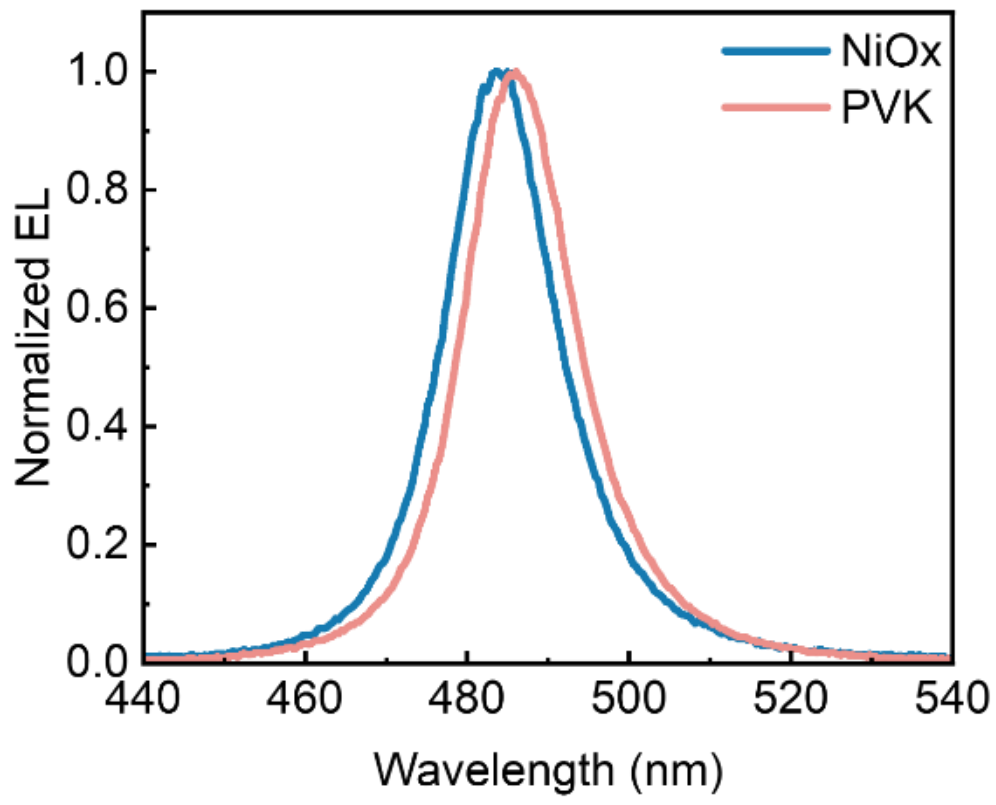

Fig. S4: EL spectra for perovskites deposited on the NiO_x/PVK/PVP and PVK/PVP HTL configurations.

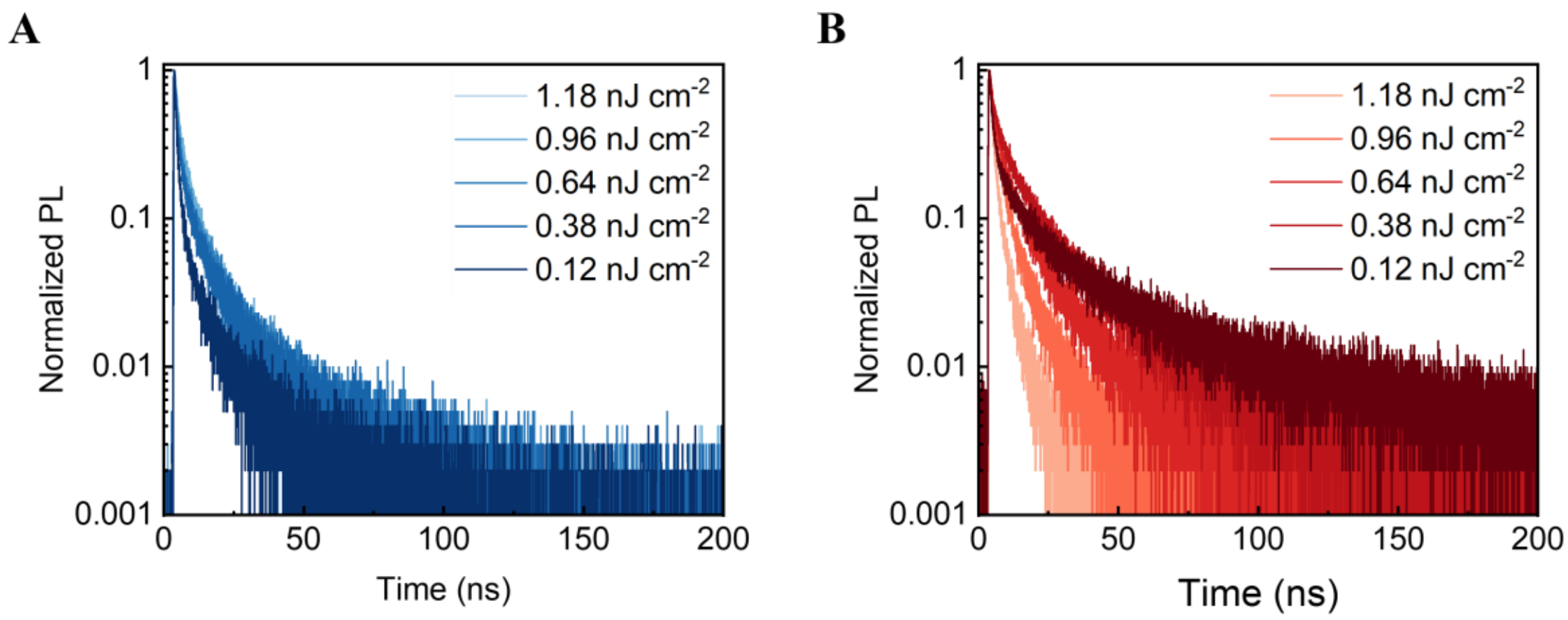

Fig. S5: Fluence-dependent TRPL decays of perovskites deposited on different HTL configurations and excited with a 405 nm pulsed laser. (A), NiO_x/PVK/PVP HTL configuration. (B), PVK/PVP HTL configuration.

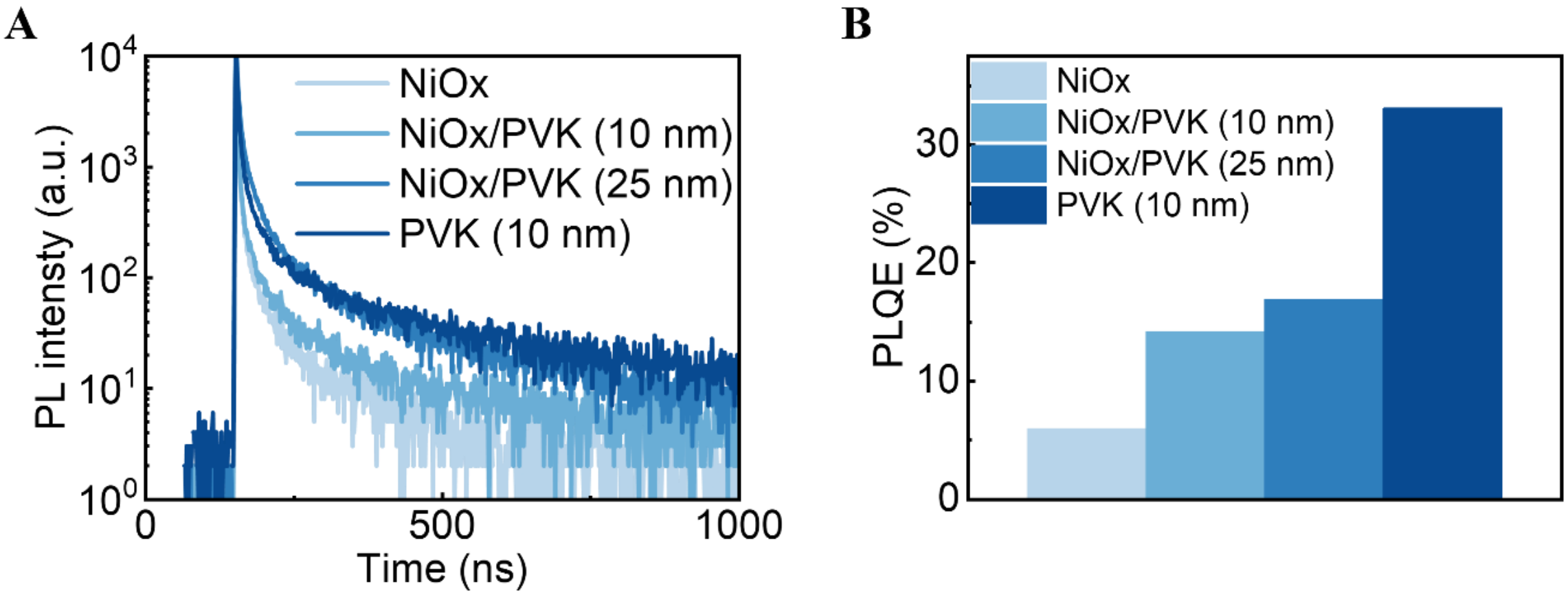

Fig. S6: Effect of PVK thickness on interfacial quenching. (A), TRPL for perovskites deposited on four different HTL stacks: NiO_x/PVP, NiO_x/PVK (10 nm)/PVP, NiO_x/PVK (25 nm)/PVP, and PVK (10 nm)/PVP. All the samples are excited by a 405 nm pulsed laser at a fluence of 0.12 nJ cm^{-2} per pulse. (B), PLQE values of perovskites deposited on those four different HTL stacks.

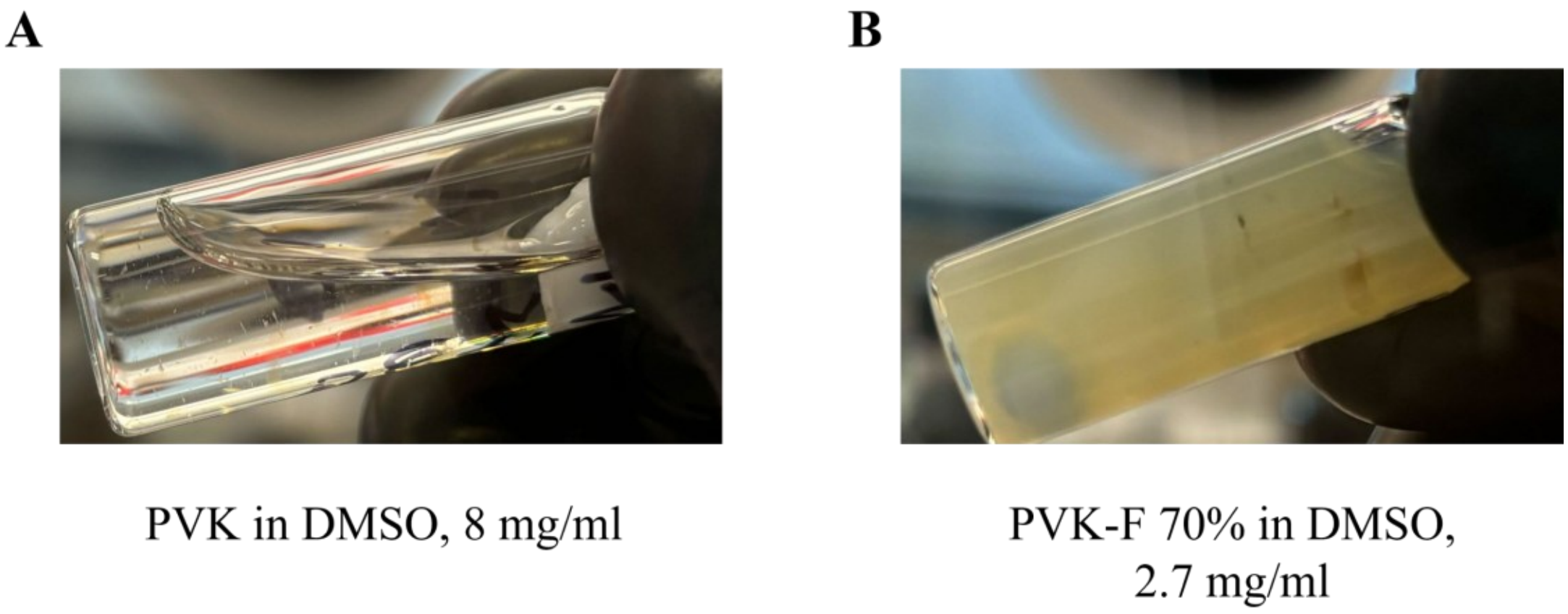

Fig. S7: Solubility tests for PVK and PVK-F 70% blend in DMSO. (A), PVK is almost fully dissolved at a concentration of 8 mg mL^{-1}, (B), PVK-F 70% blend is not dissolved even at a concentration of 2.7 mg mL^{-1}.

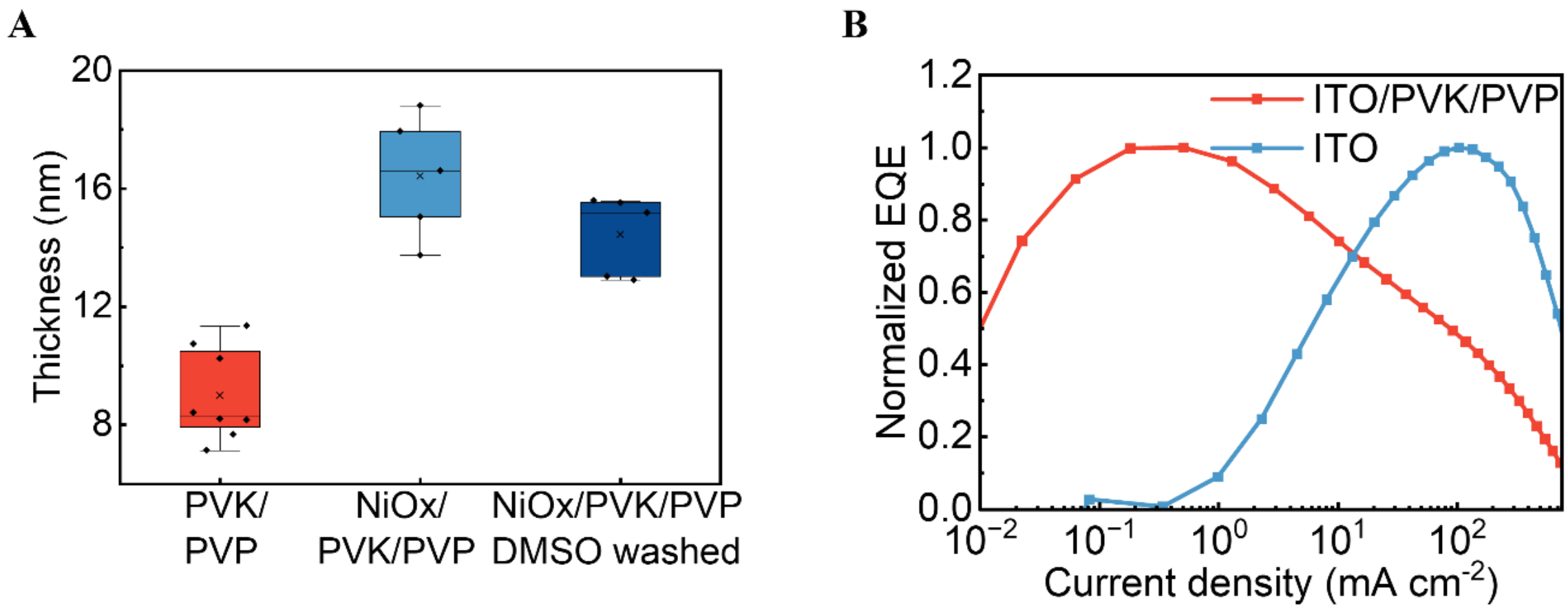

Fig. S8: Effect of DMSO washing on PVK and device behavior. (A), HTL thickness before and after DMSO washing, where only a slight reduction of NiO_x/PVK/PVP HTL thickness was observed. (B) Normalized EQE curves for perovskites deposited on PVK/PVP HTL and bare ITO substrates.

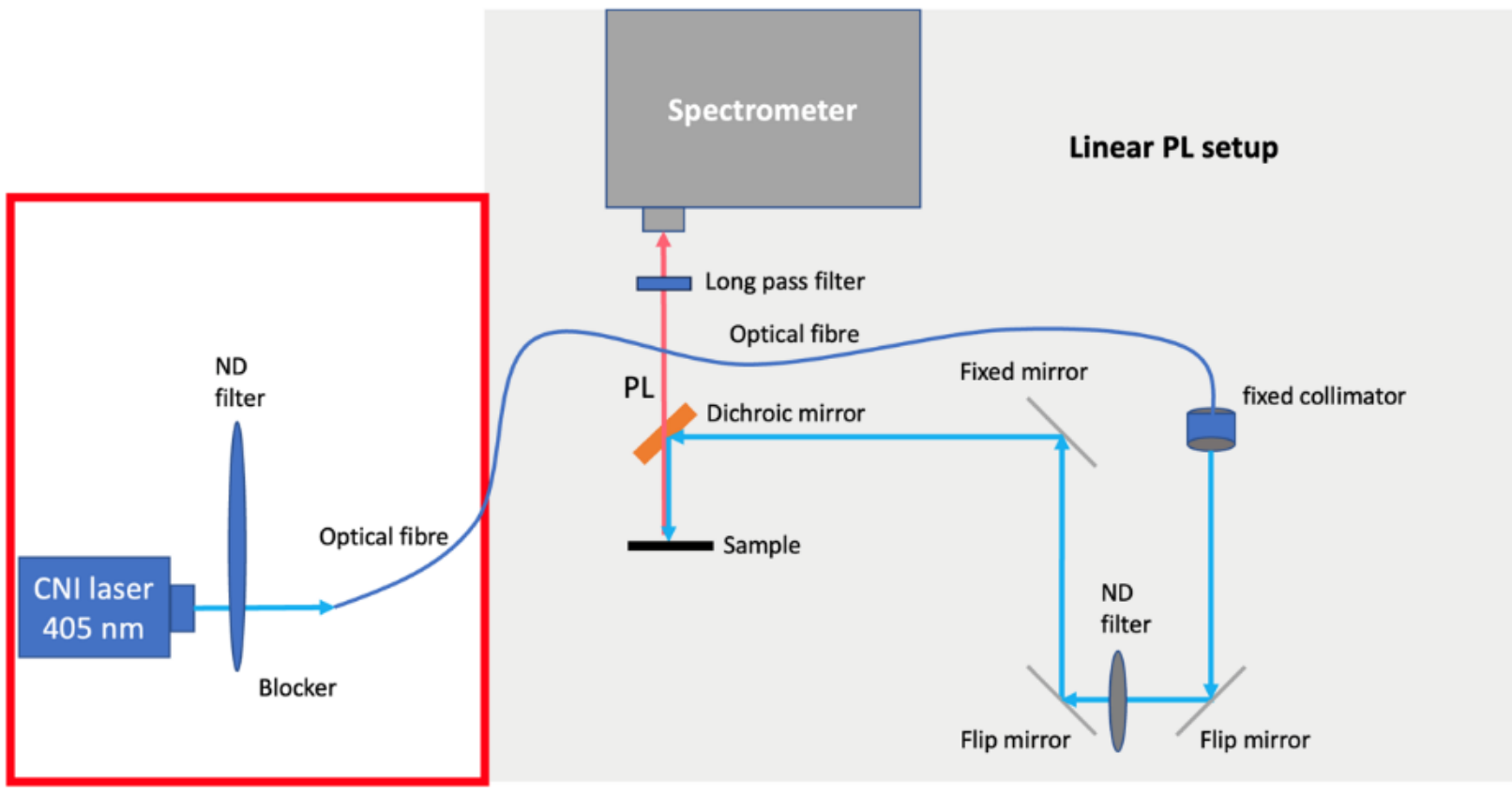

Fig. S9: Schematic of the experimental setup used for the PL stability measurements.

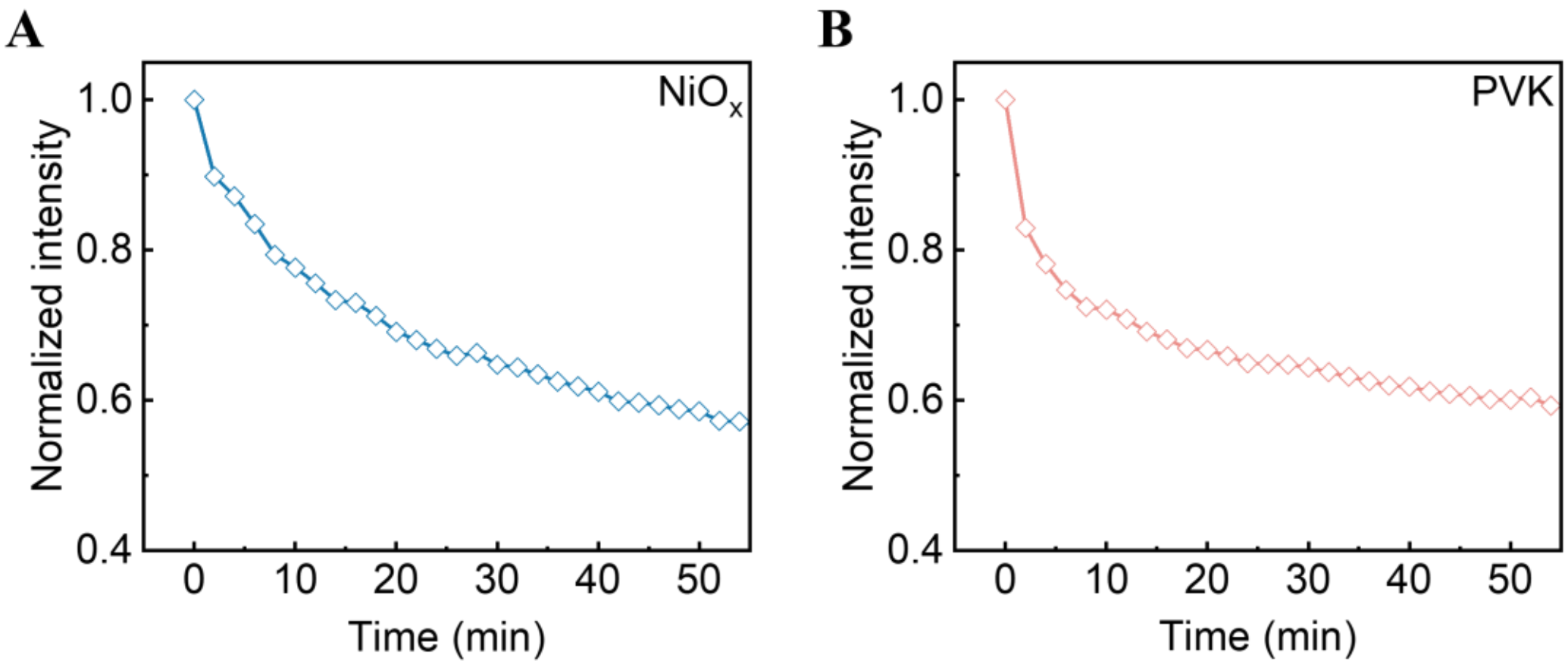

Fig. S10: PL stability for devices with different HTL configurations measured under 405 nm continuous-wave laser excitation at 28 W cm^{-2}. (A), NiO$_x$/PVK/PVP and (B), PVK/PVP HTL configurations, respectively.

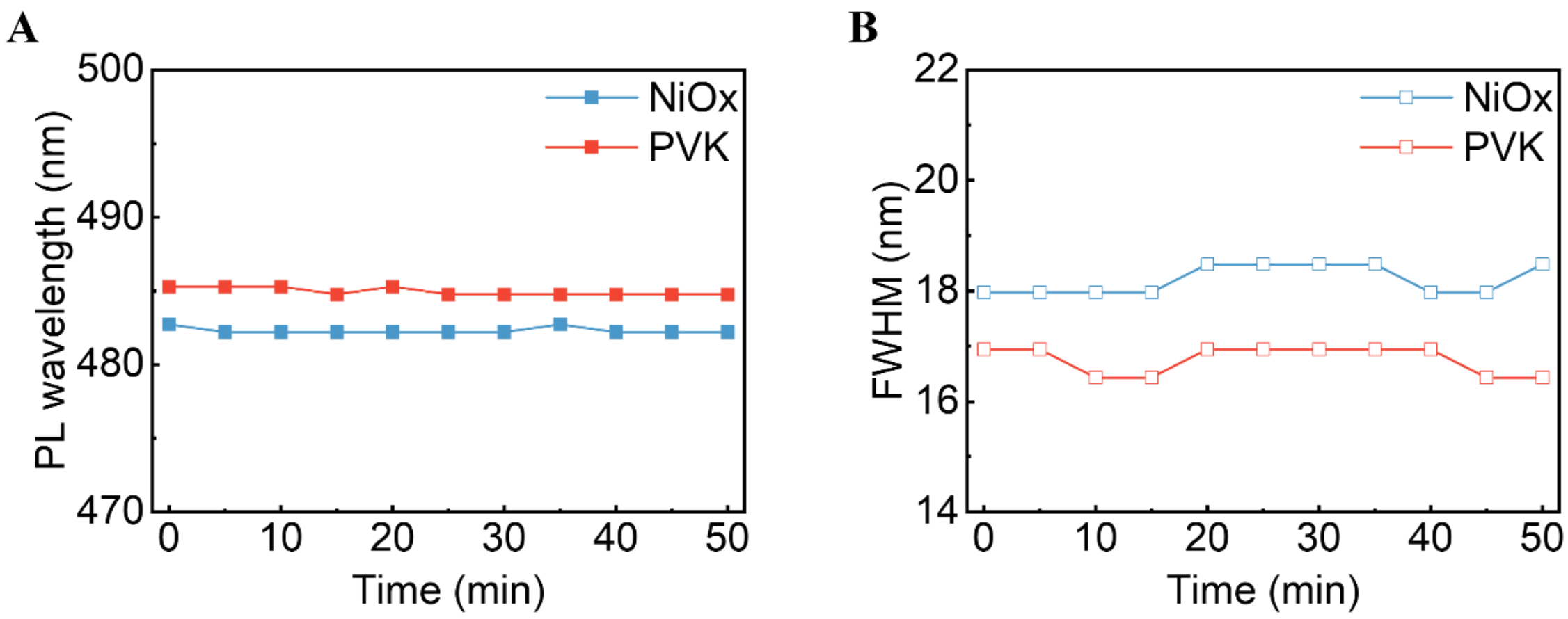

Fig. S11: PL spectral evolution of perovskite during continuous laser illumination. (A), PL peak wavelength evolution and (B), FWHM evolution for perovskites prepared on NiO$_x$/PVK/PVP (NiO$_x$-based) and PVK/PVP (PVK-based) HTL configurations under 405 nm laser excitation. For NiO$_x$-based device, it was excited at a power of 16 W cm^{-2}, while PVK-based device was excited at a power of 2.9 W cm^{-2}. These laser powers were selected to generate photogenerated current densities similar to those in EL stability measurements for different HTL configurations.

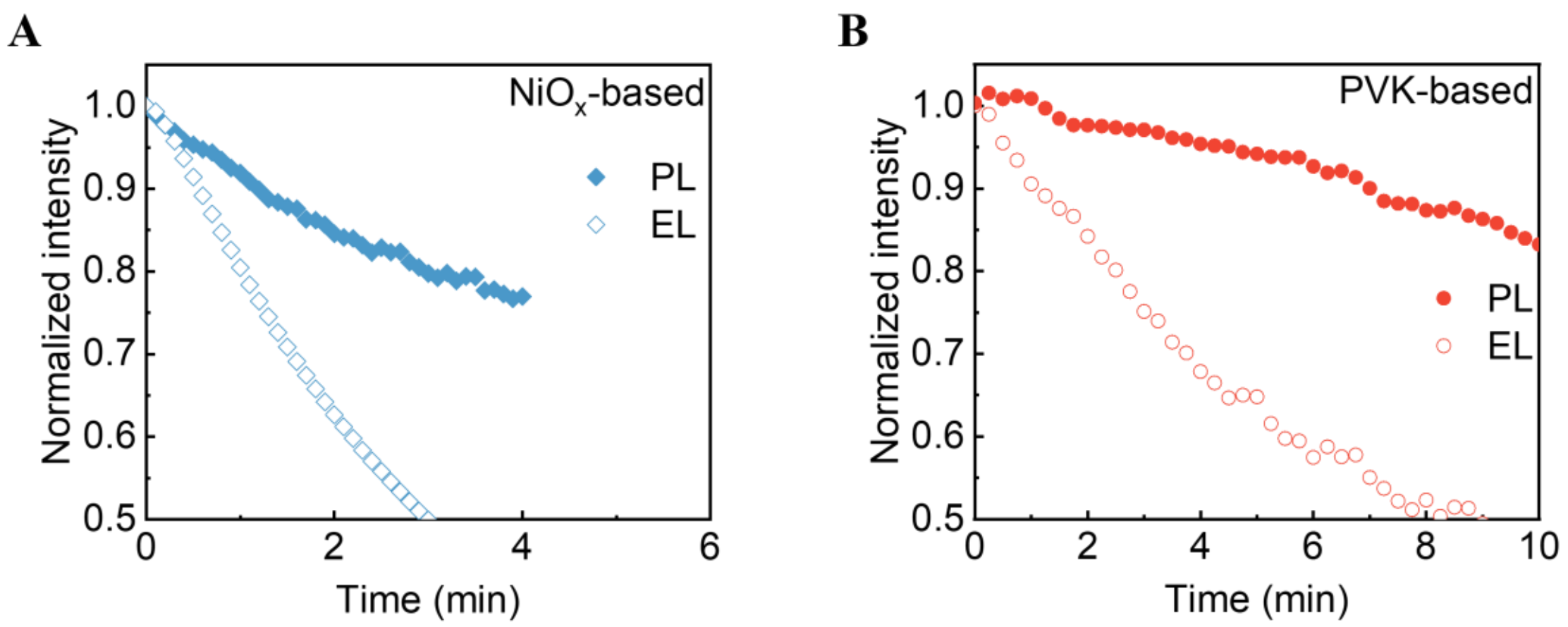

Fig. S12: Operando PL-EL tracking for devices fabricated on different HTL configurations. (A), NiO_x/PVK/PVP (operated at a current density of 4.4 mA cm^{-2}) and (B), PVK/PVP (operated at a current density of 0.8 mA cm^{-2}). The devices were operated under electrical bias to capture EL while using a 405 nm laser to excite the PL signal.

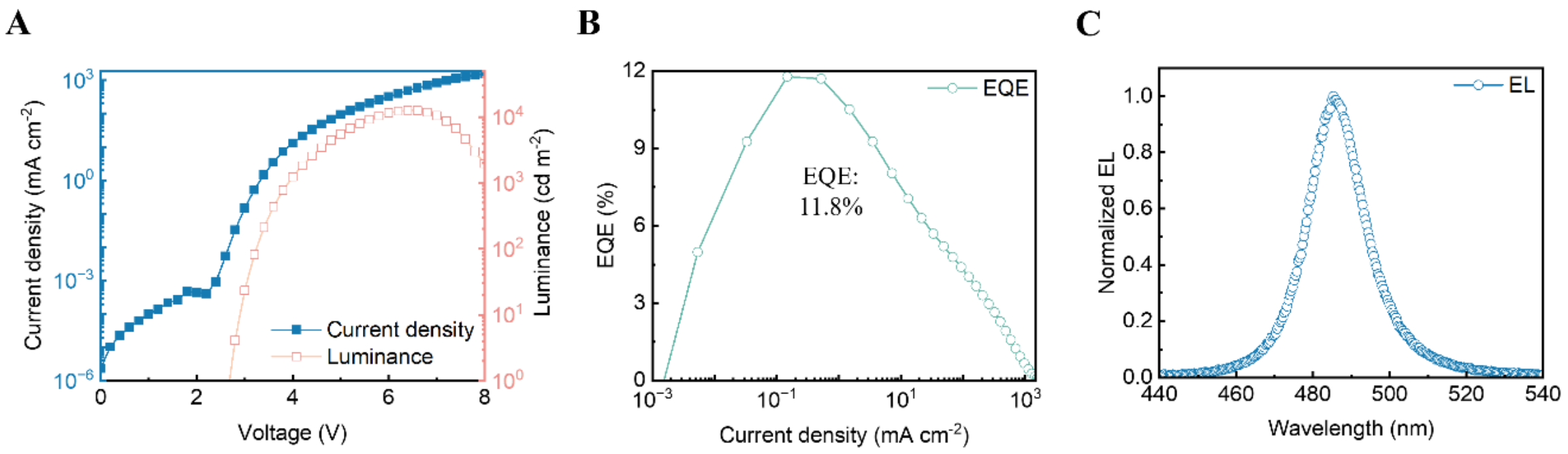

Fig. S13: Device performance for perovskites prepared on PTAA/PVK/PVP (PTAA-based) HTL configuration. (A), Current density-voltage-luminance characteristics. (B), EQE-current density characteristics, and (C), EL spectrum.

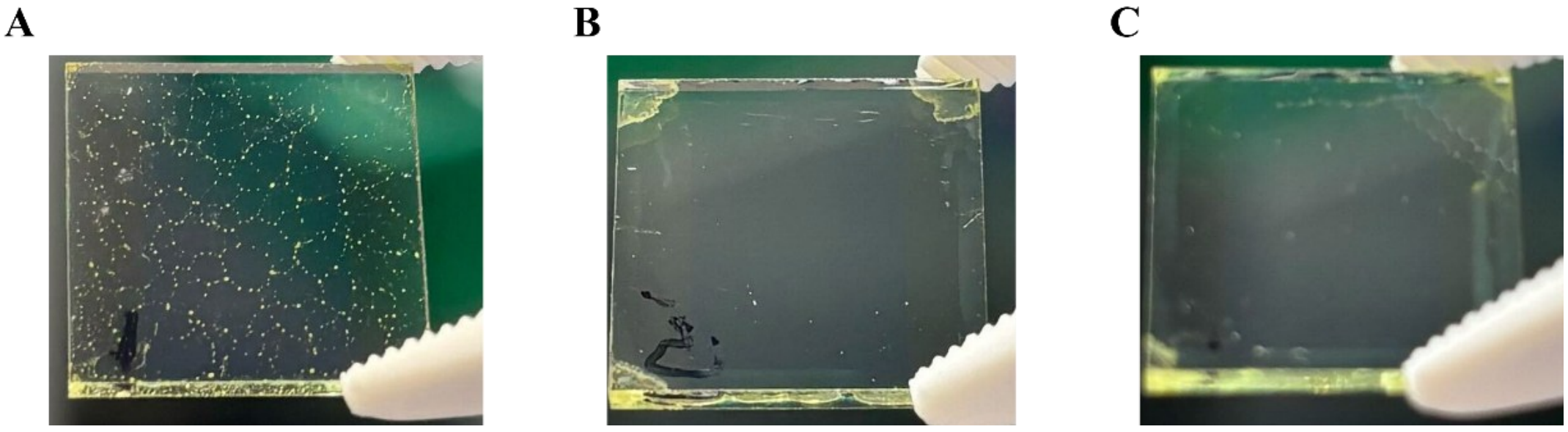

Fig. S14: Images of perovskites directly spin-coated on PVK:PTAA blend HTLs. (A), PTAA, (B), PVK:PTAA polymer blend (50% PTAA: 50% PVK in weight ratio), and (C), PVK.

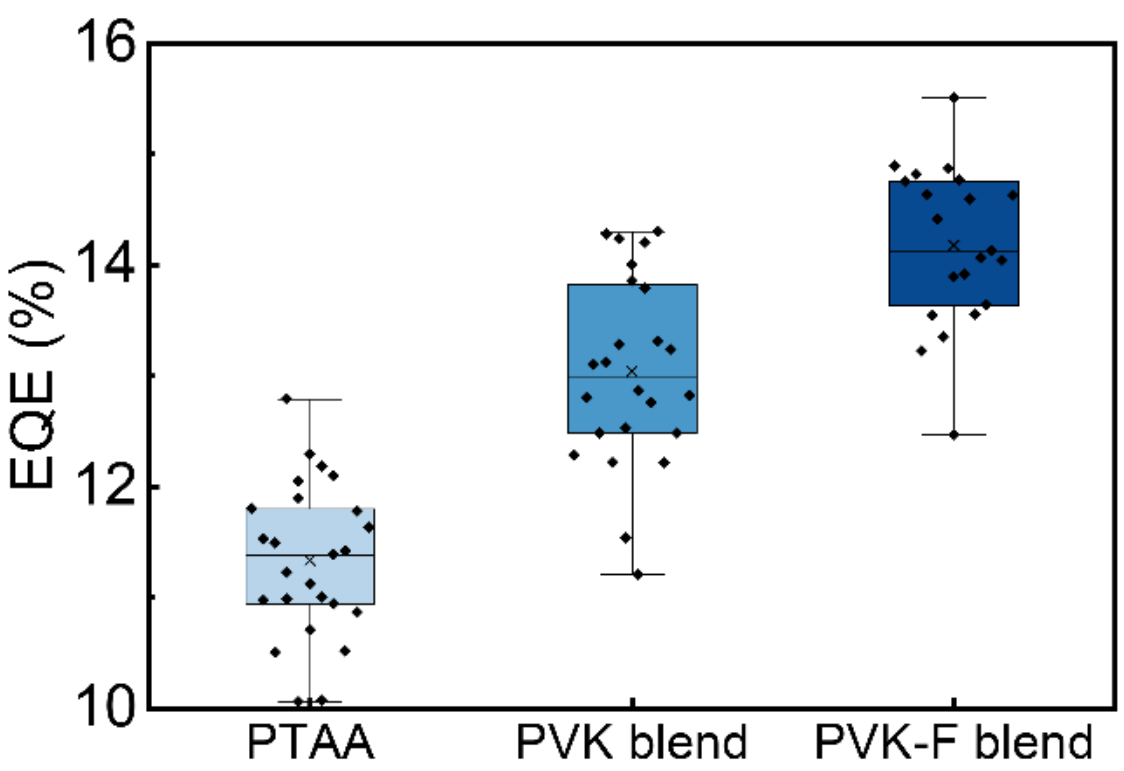

Fig. S15: Peak-EQE distributions for devices fabricated in the same batch using three HTL configurations: PTAA/PVK/PVP (PTAA-based), PVK:PTAA blend/PVK/PVP (PVK blend-based), and PVK-F:PTAA blend/PVK/PVP (PVK-F blend-based).

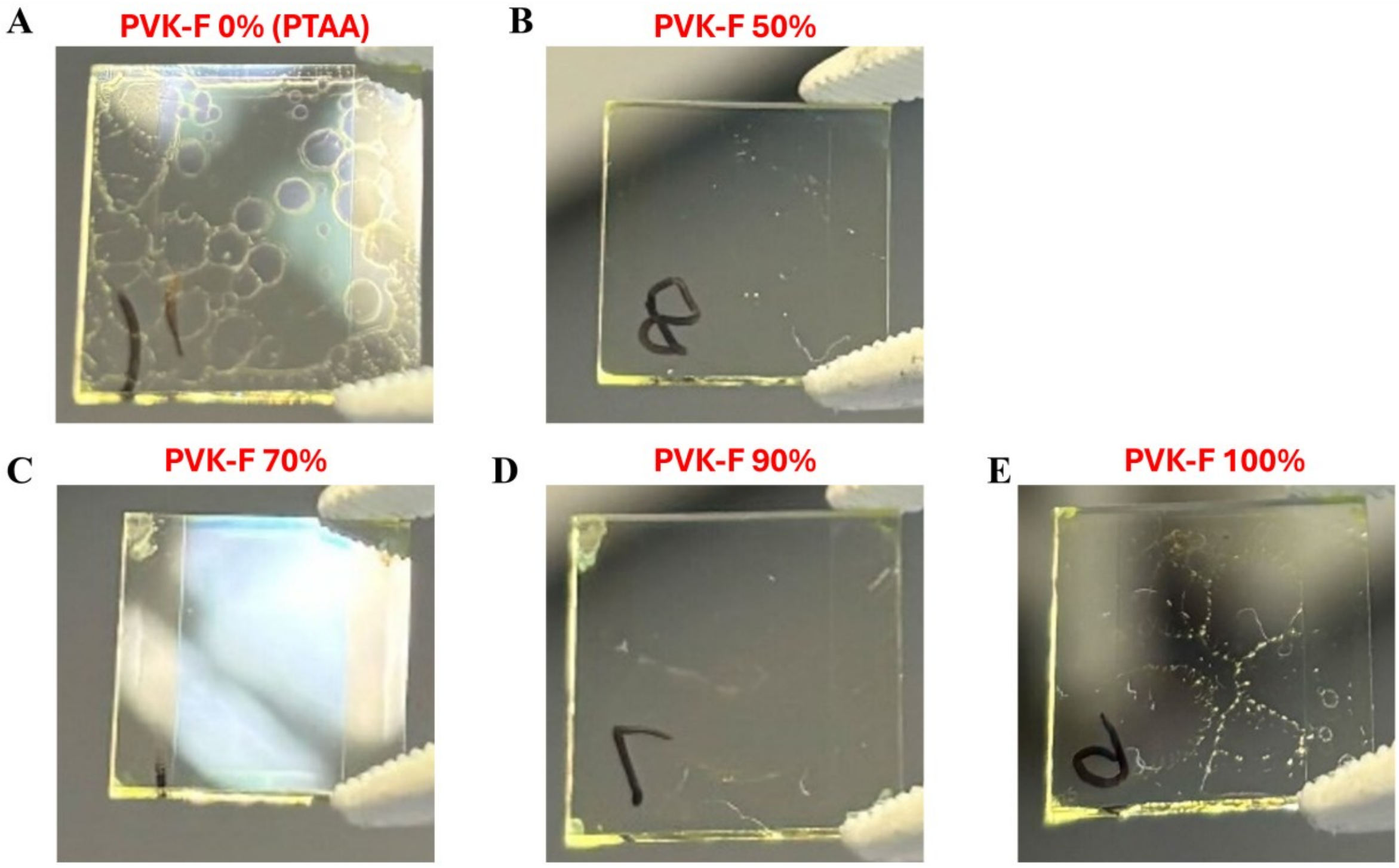

Fig. S16: Images of perovskites spin-coated on PVK-F:PTAA polymer blends with different weight ratios of PVK-F. (A), 0%, (B), 50%, (C), 70%, (D), 90%, and (E), 100%.

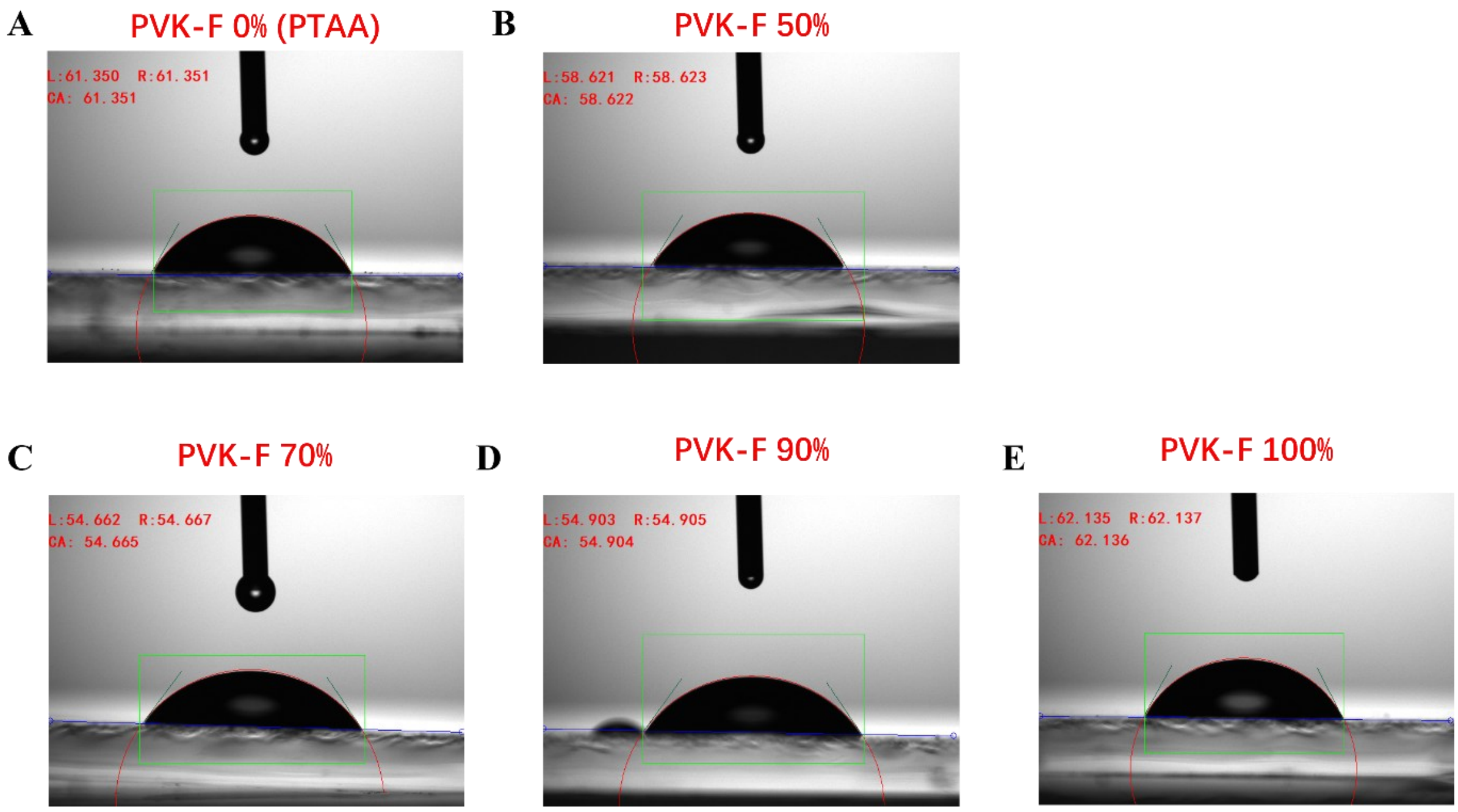

Fig. S17: Water contact angle measurement for PVK-F:PTAA polymer blends with different weight ratios of PVK-F. (A), 0%, (B), 50%, (C), 70%, (D), 90%, and (E), 100%.

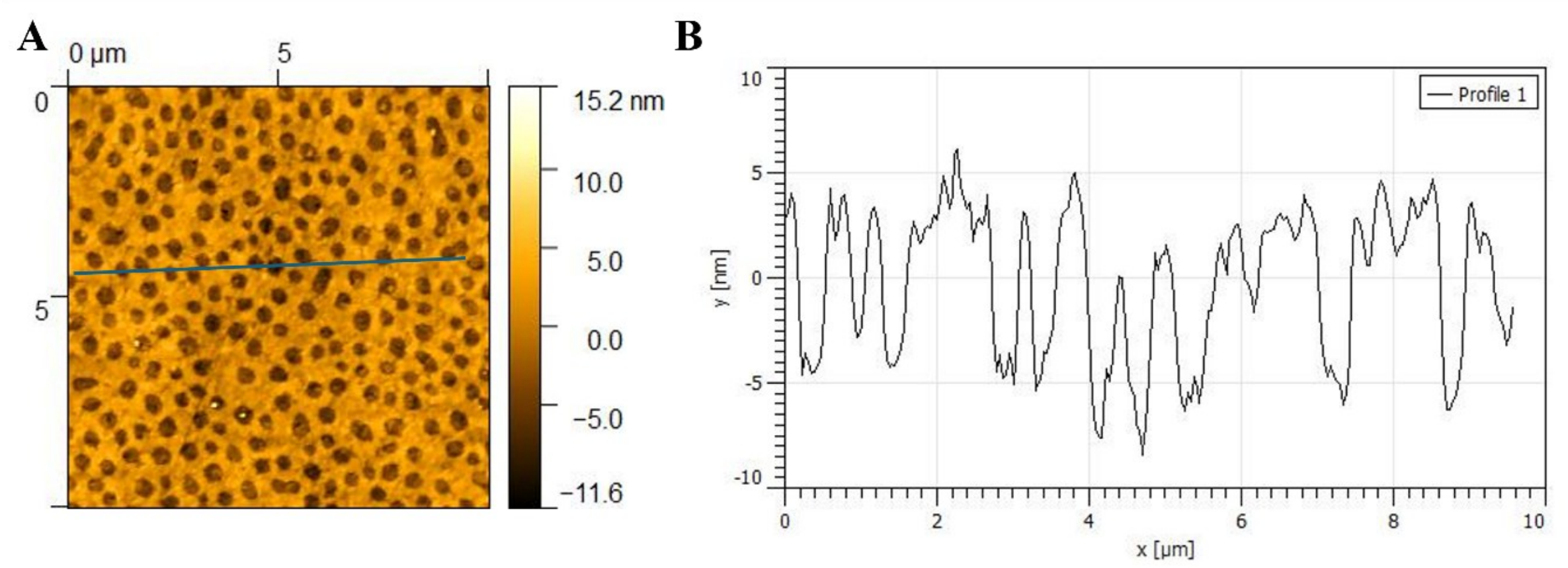

Fig. S18: AFM image for the blending polymer. (A), 30% of PTAA and 70% of PVK-F (9mg ml^{-1}), (B), The height profile extracted from the blue line in (A), showing pores approximately 8 nm deep and 400 nm wide.

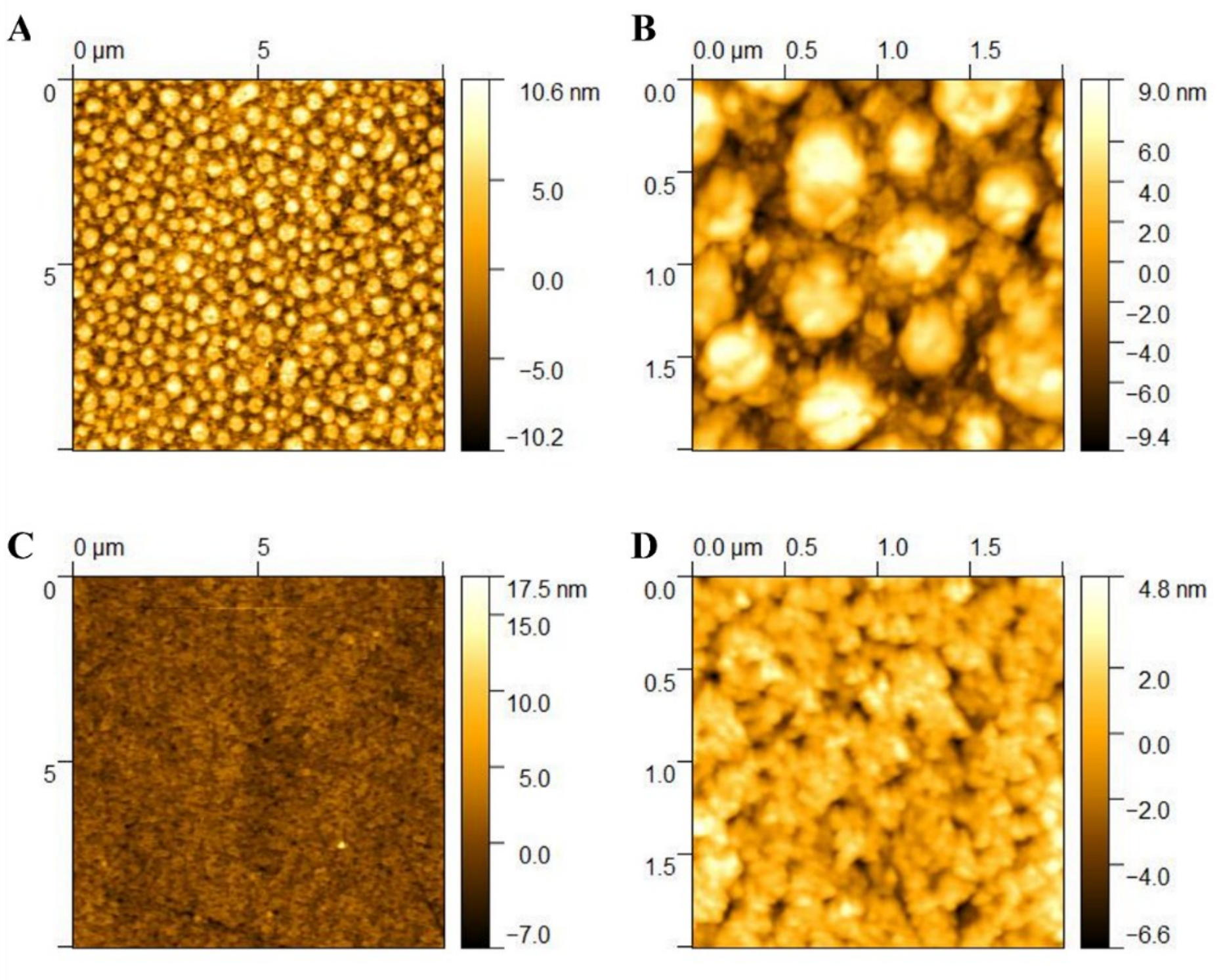

Fig. S19: AFM images for blending polymers with different PVK-F and PTAA ratios. (A) and (B), 50% of PTAA and 50% of PVK-F (9 mg ml^{-1}), (C) and (D), 10% of PTAA and 90% of PVK-F (9 mg ml^{-1}).

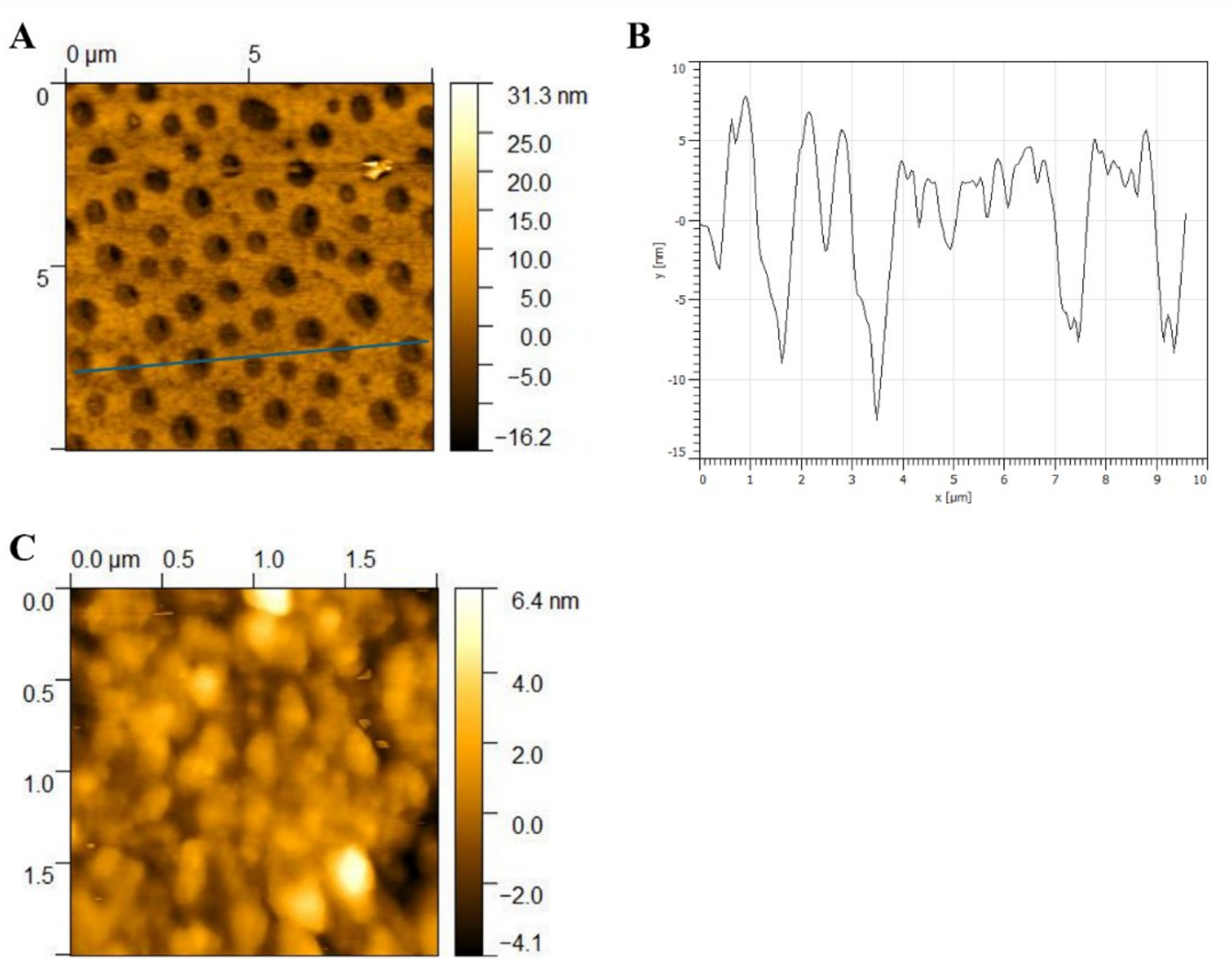

Fig. S20: AFM characterization of blends containing 70 wt% PVK-F and 30 wt% PTAA at different concentrations. (A) AFM image at 20 mg ml^{-1}. (B) Height profile along the line in (A), showing pores approximately 15 nm deep and 1000 nm wide. (C) AFM image at 3 mg ml^{-1}.

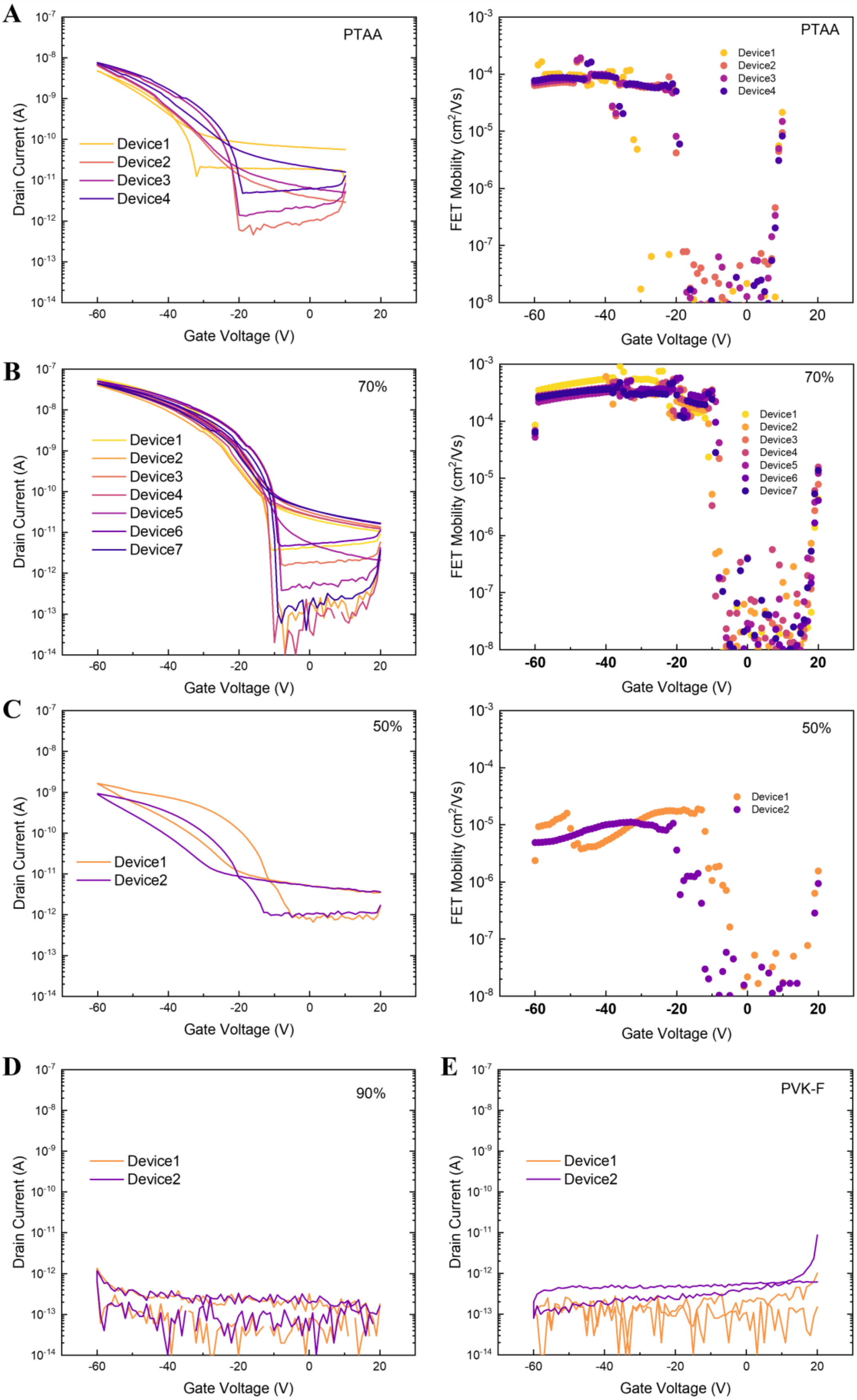
A
PTAA
Device1
Device2
Device3
Device4
Drain Current (A)
Gate Voltage (V)
FET Mobility (cm²/Vs)
B
70%
Device5
Device6
Device7
C
50%
D
90%
E
PVK-F

Fig. S21: Transfer characteristics and gate voltage dependent mobility for PVK-F:PTAA blend films. (A), PTAA, (B), PVK-F 70%, (C), PVK-F 50%, (D), PVK-F 90% and (E), PVK-F 100%.

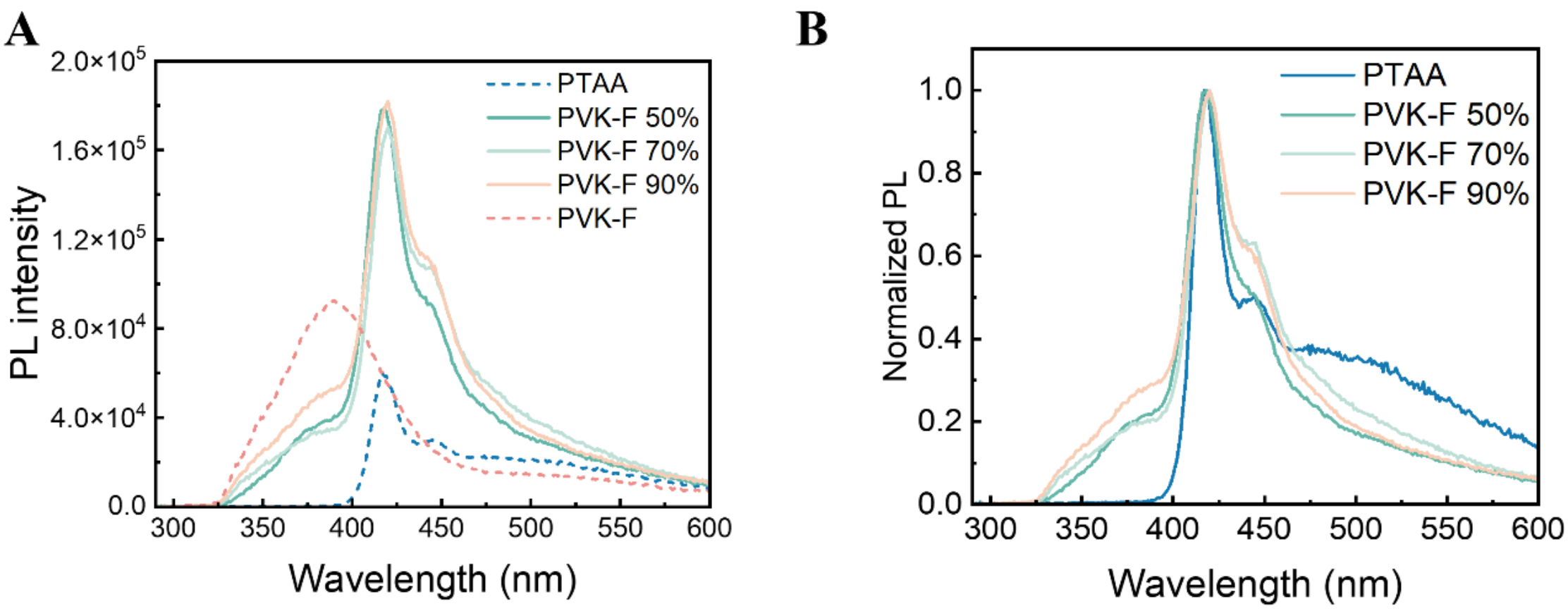

Fig. S22: PL spectra of PVK-F:PTAA blend films. (A), PL spectra excited at 310 nm for PVK-F:PTAA blend polymers with different weight ratios of PVK-F and (B), the corresponding normalized PL spectra.

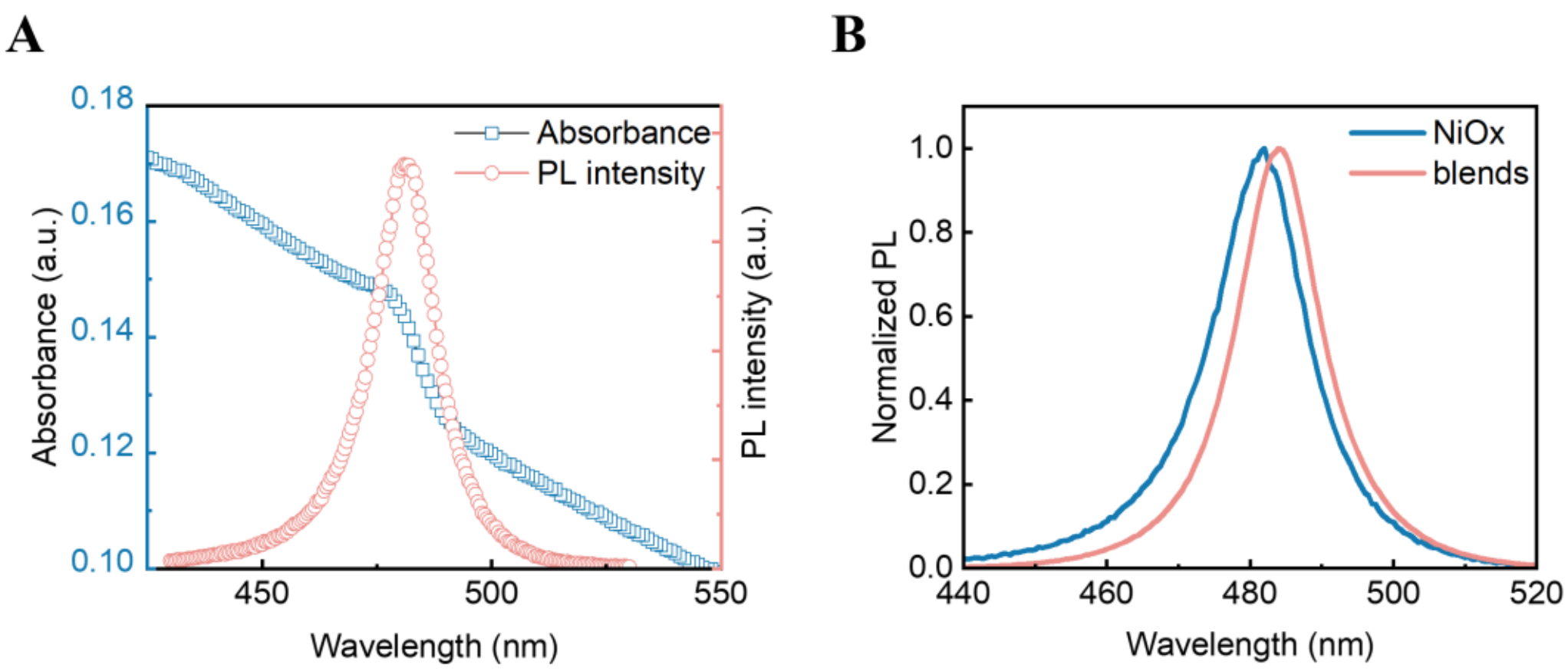

Fig. S23: Optical properties of perovskite films on NiO_x-based and blend-based HTLs. (A), Absorption and PL spectra for perovskites on NiO_x/PVK/PVP configuration. a.u., arbitrary units. (B), Comparison of PL spectra for perovskites on NiO_x/PVK/PVP and PVK-F 70% blend/PVK/PVP HTL configurations.

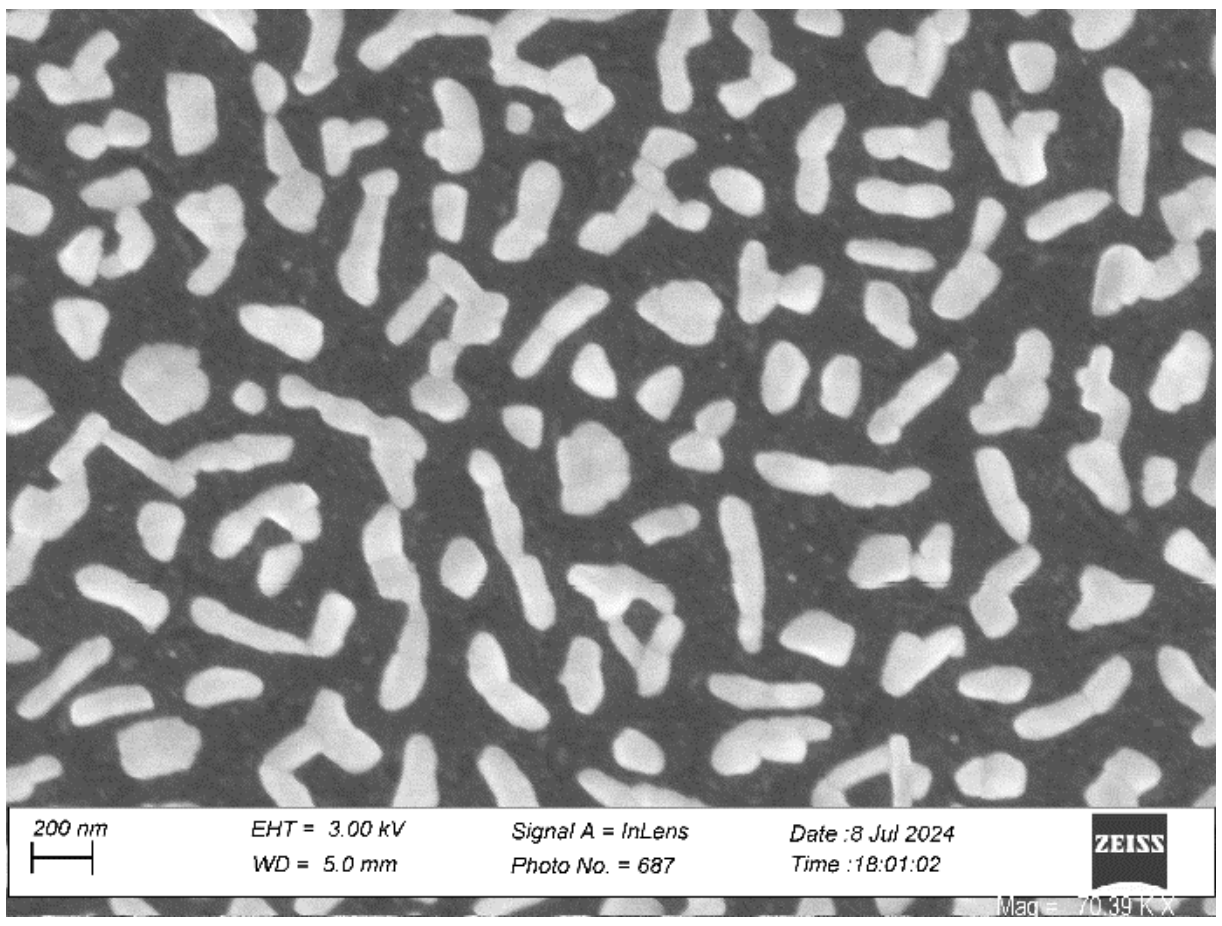

Fig. S24: SEM image for perovskites spin-coated on PVK-F 70% blend/PVK/PVP HTL configuration.

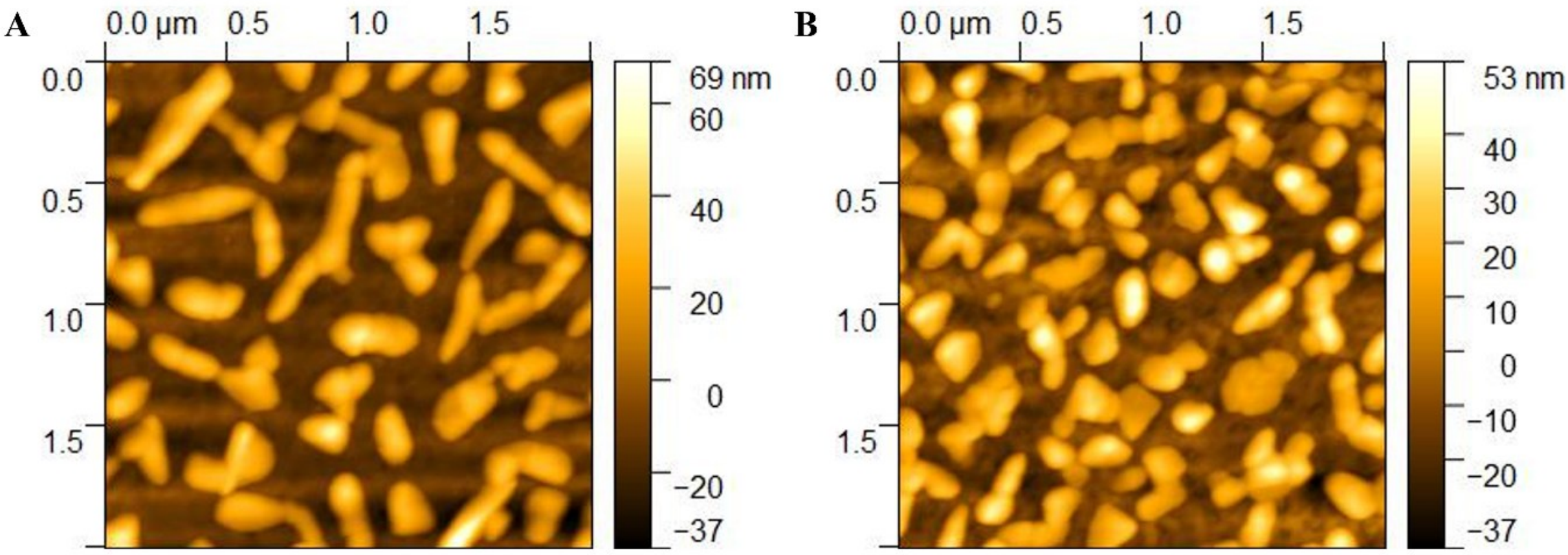

Fig. S25: AFM images of perovskites spin-coated on different HTL configurations. (A), PVK-F 70% blend/PVK/PVP, and (B), NiO_x/PVK/PVP.

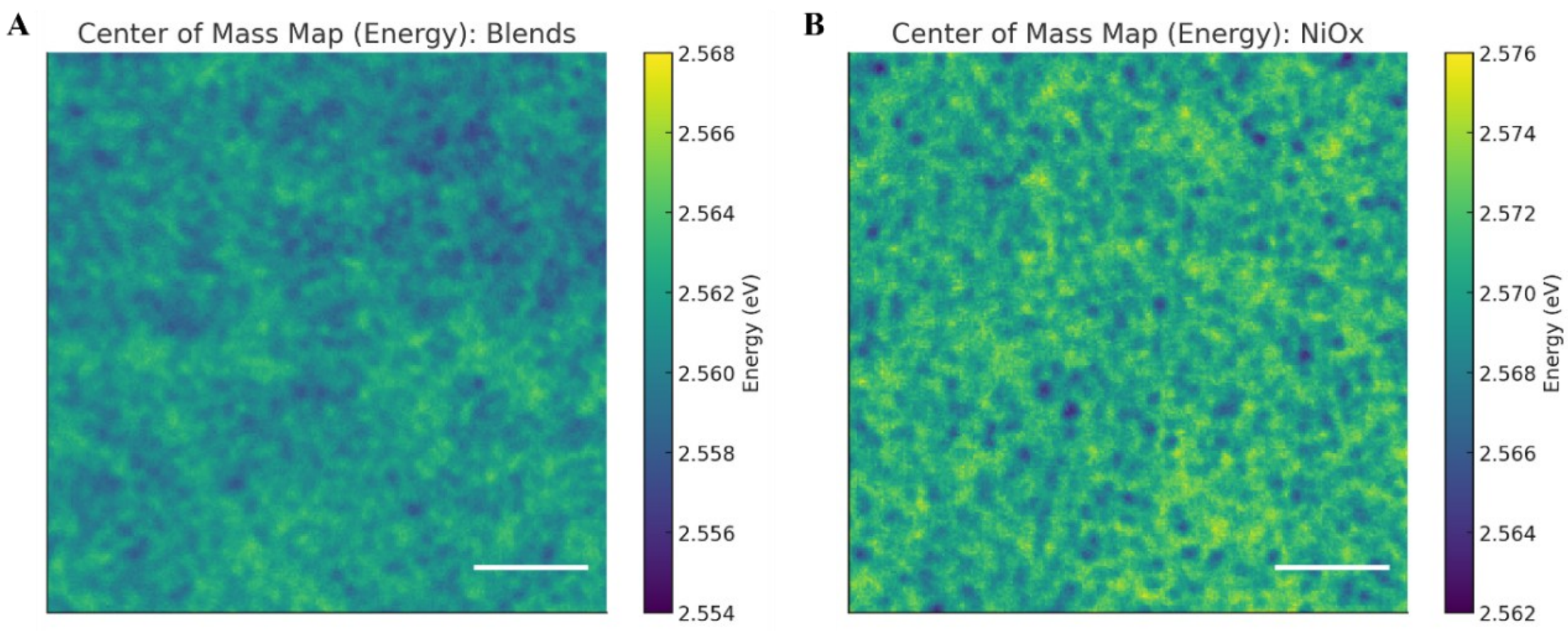

Fig. S26: Center of mass (COM) energy maps for perovskites prepared on different HTL configurations. (A), PVK-F 70% blend/PVK/PVP and (B), NiO_x/PVK/PVP HTL configurations. The scale bars are 5 µm.

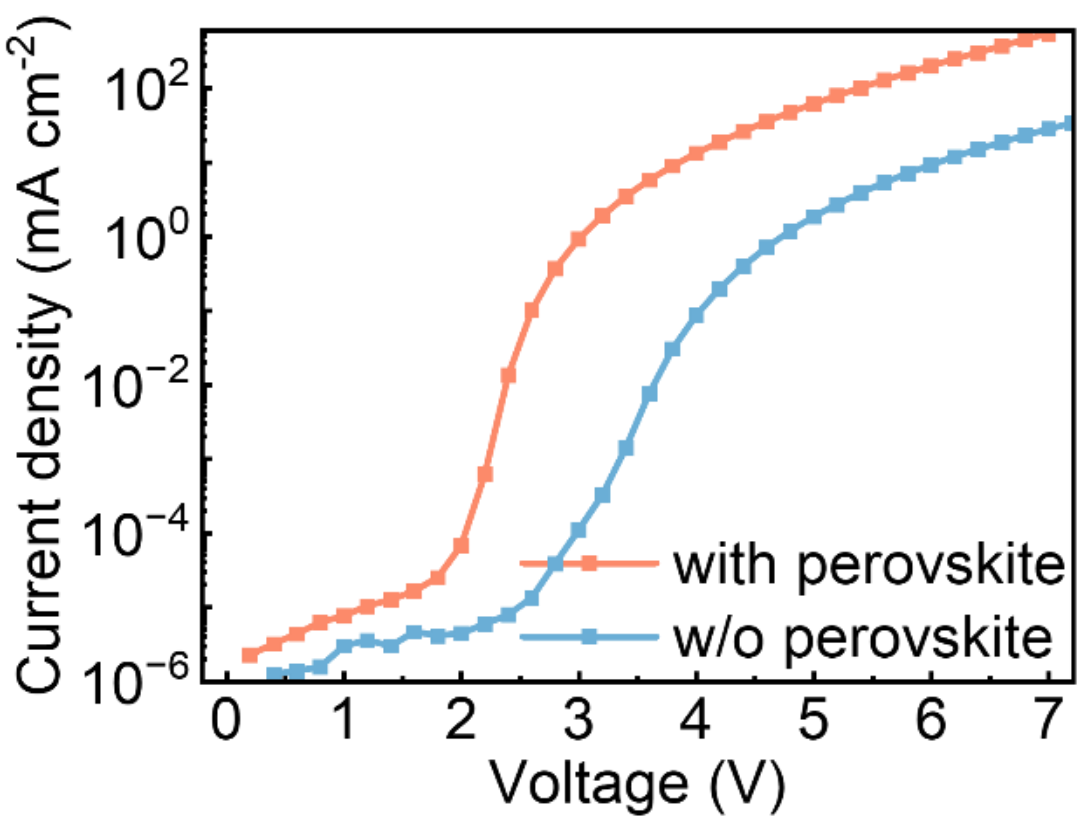

Fig. S27: Current density-voltage characteristics for device structure of ITO/ PVK-F 70% blend/ PVK/PVP/perovskite (with or without)/TPBi/LiF/Al to assess current leakage from porous blend morphology.

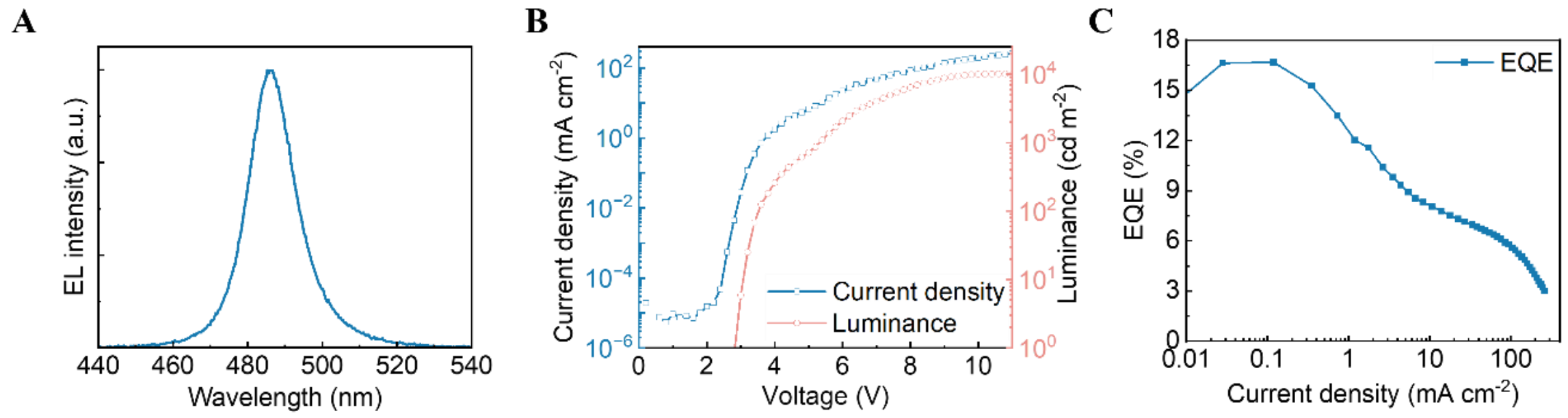

Fig. S28: 3D blue PeLEDs containing the control perovskite as the emitting layer and fabricated using PVK-F 70% blend/PVK/PVP HTL configuration. (A), EL spectrum, (B), Current density-voltage-luminance characteristics for devices and (C), EQE-current density characteristics of PeLEDs.

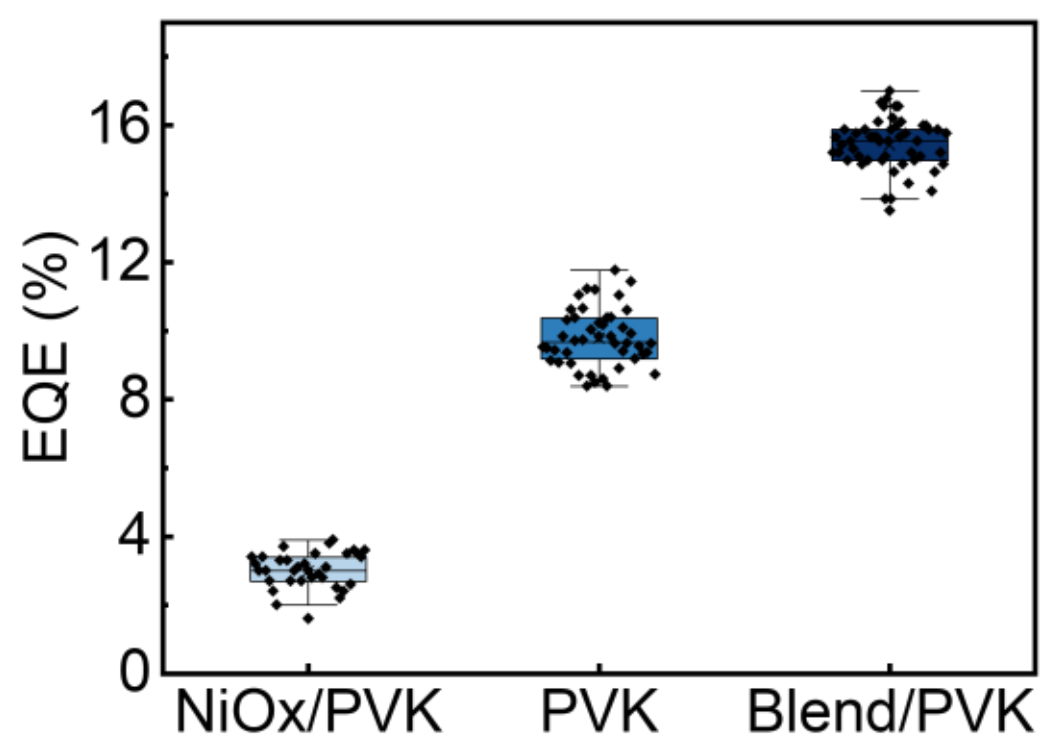

Fig. S29: Peak-EQE distributions for control perovskite deposited onto different HTL configurations. The average peak EQEs show 2.9%, 9.8%, and 15.4% for perovskite deposited on NiO_x/PVK/PVP, PVK/PVP, and PVK-F 70% blend /PVK/PVP HTL configuration, respectively.

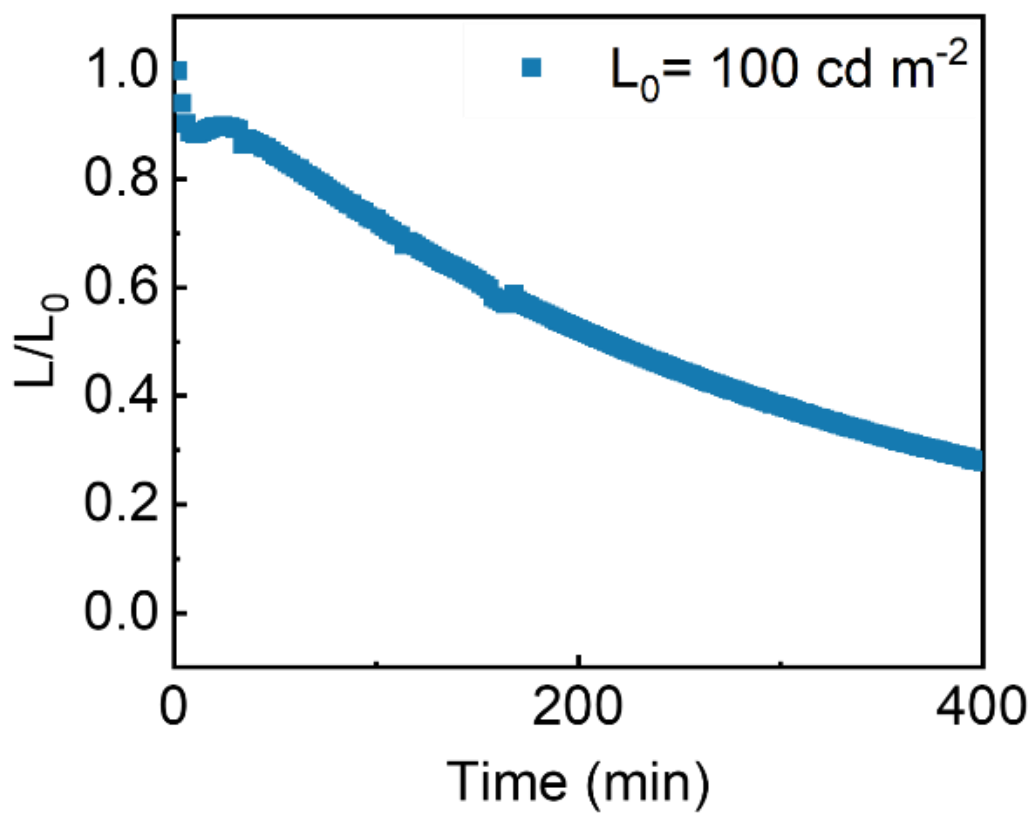

Fig. S30: Operational lifetime of a device fabricated using the PVK-F 70% blend/PVK/PVP HTL configuration at an initial luminance of 100 cd m^{-2}.

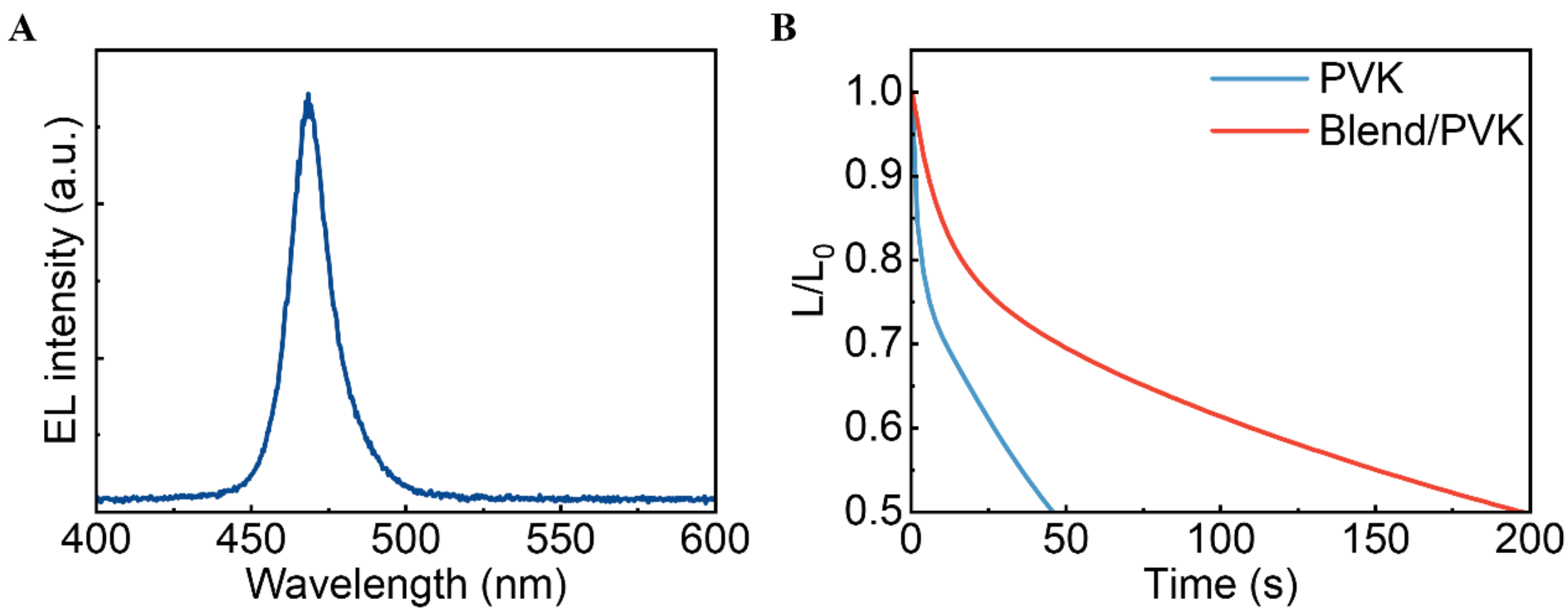

Fig. S31: Deep-blue device stability enabled by the PVK-F 70% blend-based HTL. (A), EL spectrum for the 3D deep-blue emission perovskite composition deposited on PVK-F 70% blend/ PVK/PVP (blend-based) HTL configuration. (B), Operational lifetimes of devices with and without the PVK-F 70% blend in HTL configuration biased at constant current density to get an initial luminance of 100 cd m^{-2}.

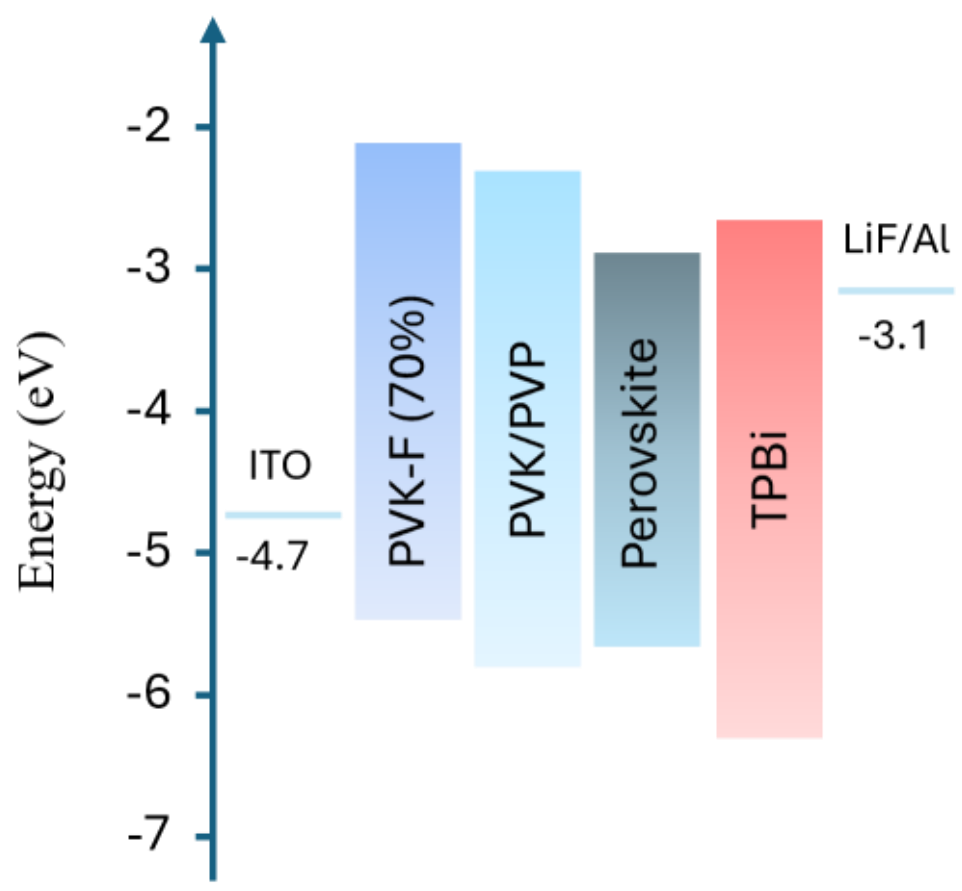

Fig. S32: Energy level diagram for the devices.

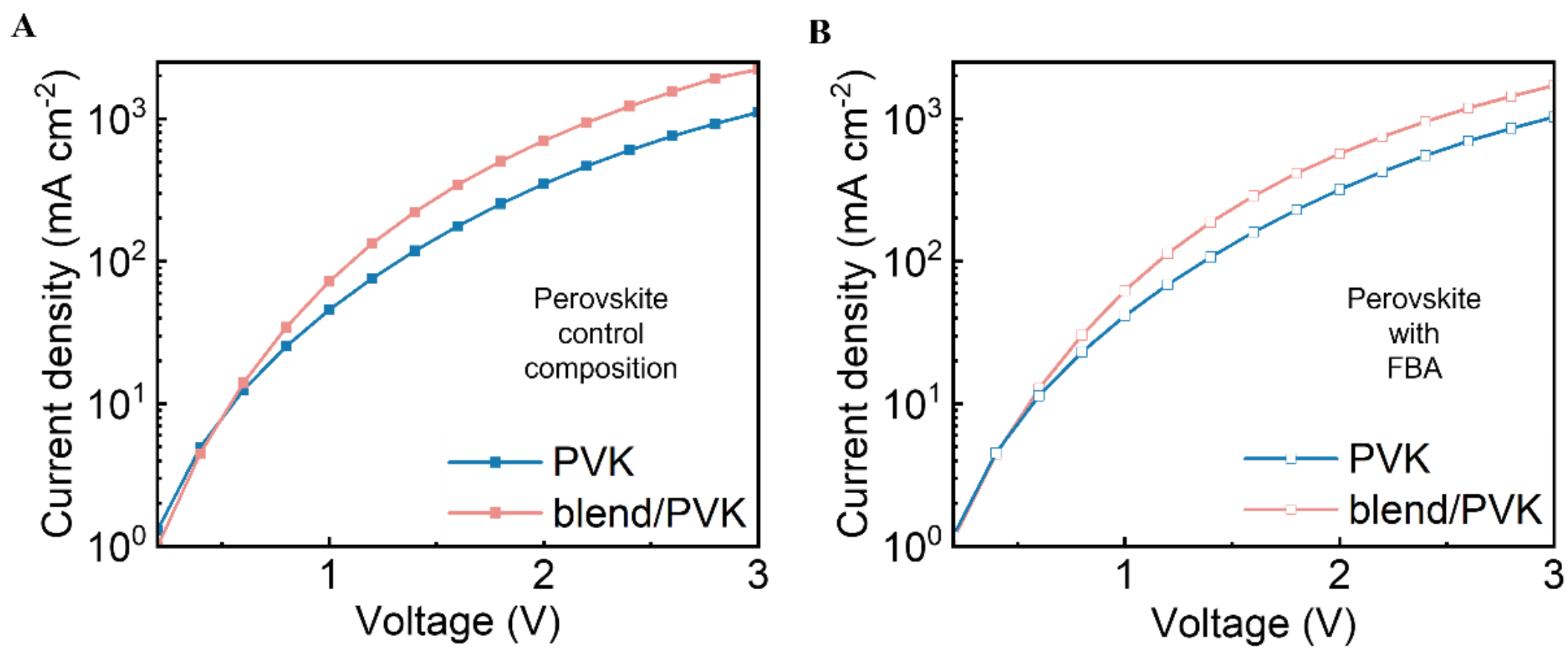

Fig. S33: Current density-voltage characteristics for hole-only devices with a structure of ITO/PVK-F 70% blend (with or without)/PVK/PVP/perovskite/TFB/MoO_x/Au. (A), Control perovskite composition, (B), Perovskite modified with FBA.

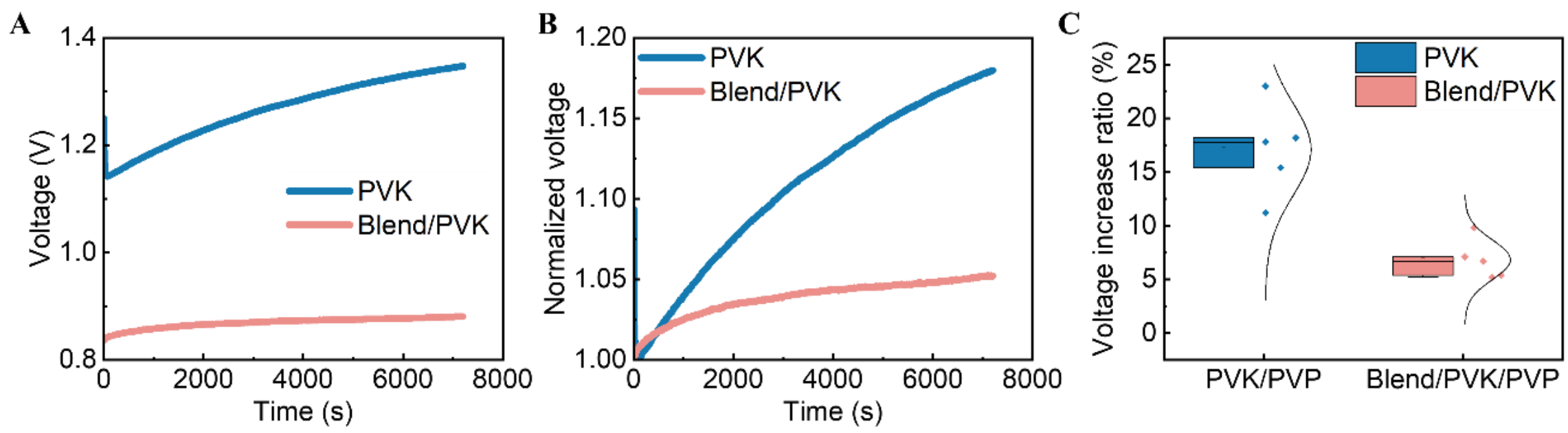

Fig. S34: Hole-only device stability with a structure of ITO/PVK-F 70% blend (with or without) /PVK/PVP/perovskite/TFB/MoO_x/Au operated at a constant current density of 50 mA cm^{-2}. (A), Voltage evaluation over time, (B), Normalized voltage evaluation over time, (C), Statistical distribution of voltage increase ratio operated with multiple hole-only devices.

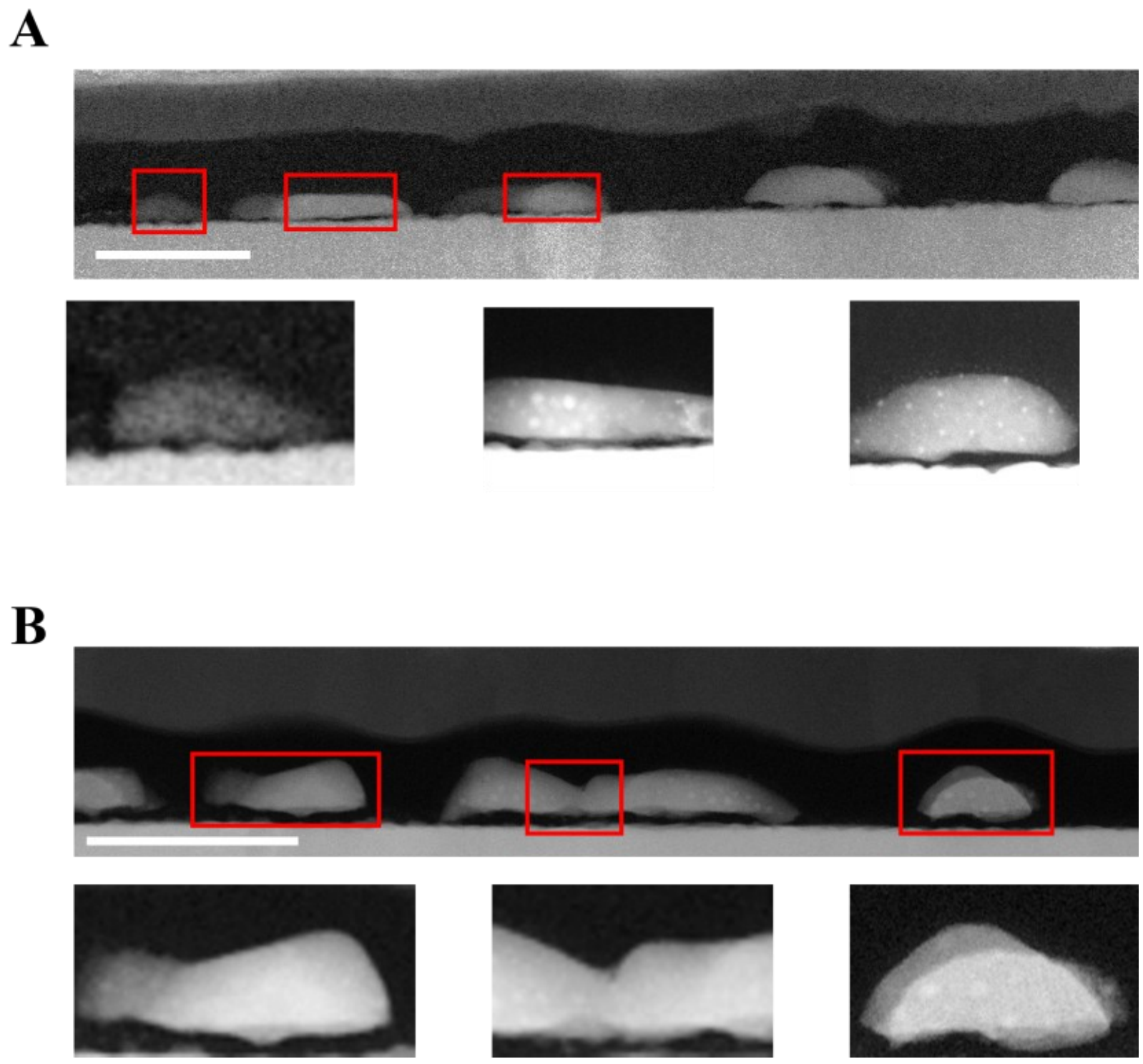

Fig. S35: HAADF-STEM images for PeLEDs with different HTL configurations after 35 minutes of operation at a constant current density of 1 mA cm^{-2}. (A) PVK/PVP and (B) PVK-F 70% blend/PVK/PVP HTL configuration. Both devices show dimmer emission than the fresh devices but retain homogeneous emission. The red rectangles highlight the zoomed-in area. Scale bars are 200 nm.

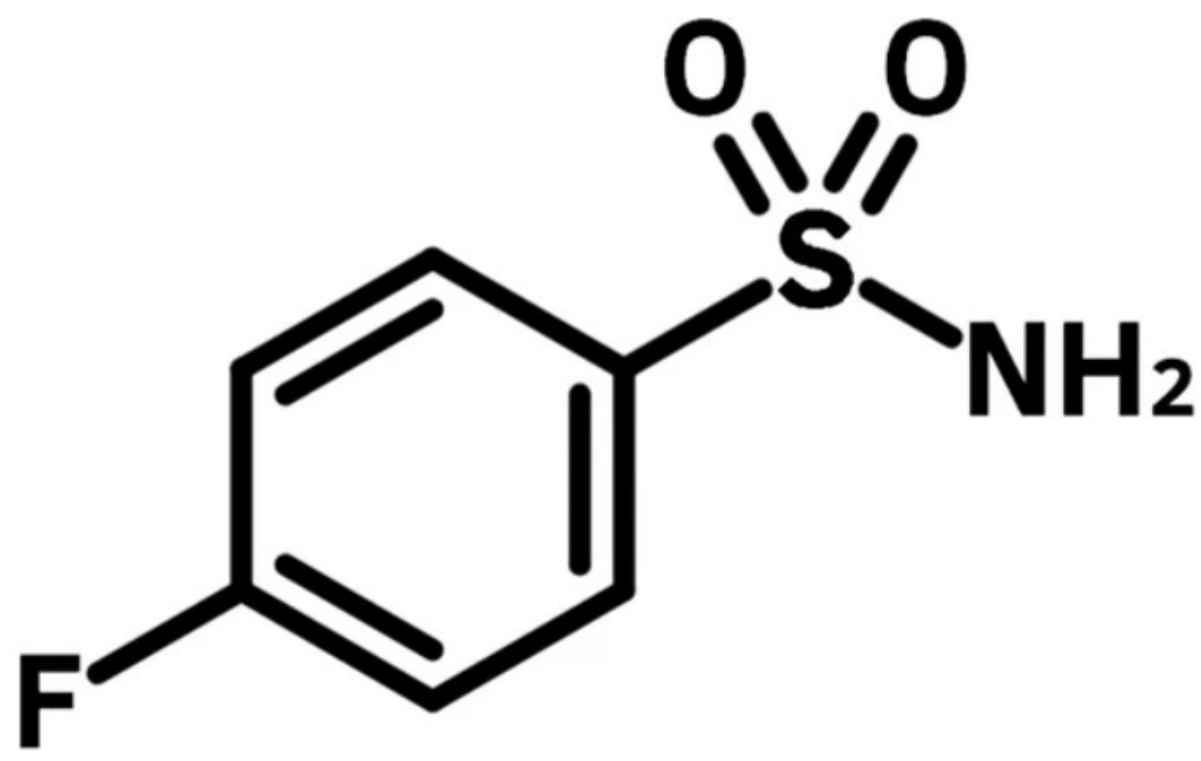

Fig. S36: Molecular structure of 4-fluorobenzenesulfonamide (FBA).

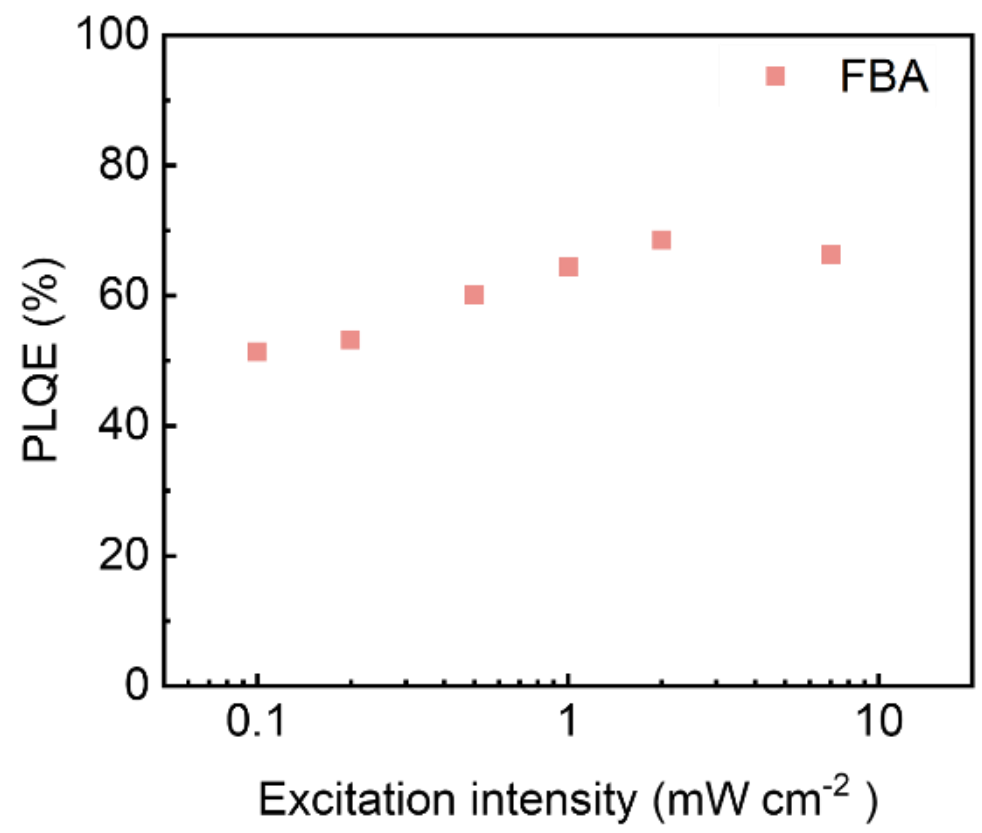

Fig. S37: PLQE for FBA-modified perovskites deposited on PVK-F 70% blend/PVK/PVP HTL configuration.

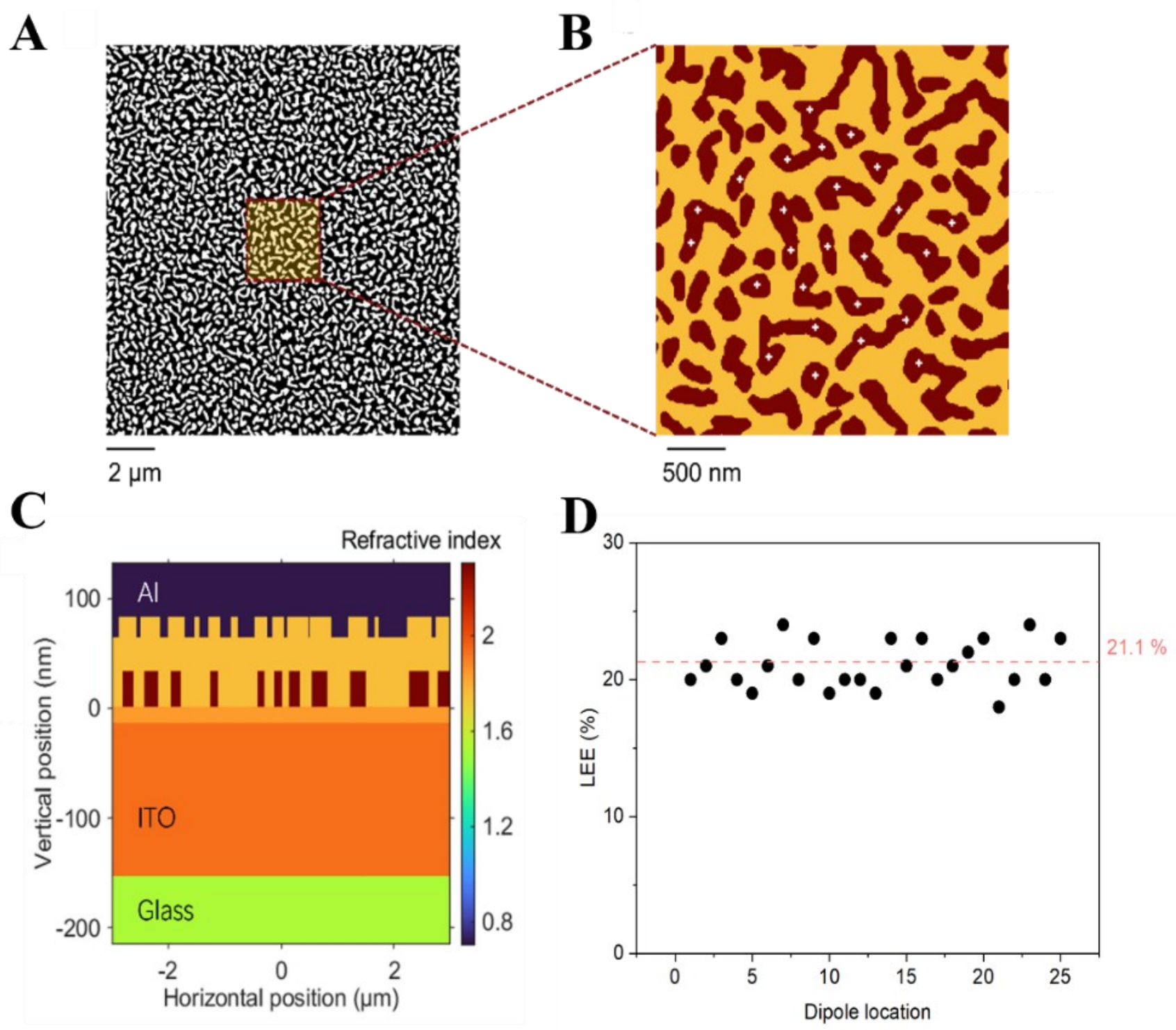

Fig. S38: Simulation model for light-outcoupling analysis. (A), SEM for FBA-modified perovskites prepared on PVK-F 70% blend/PVK/PVP HTL configuration. (B), Zoomed-in region for simulation. (C), Thickness for different layers for simulation. (D), Simulated light-extraction efficiencies for perovskite prepared on NiO_x/PVK/PVP as a function of grain length or period.

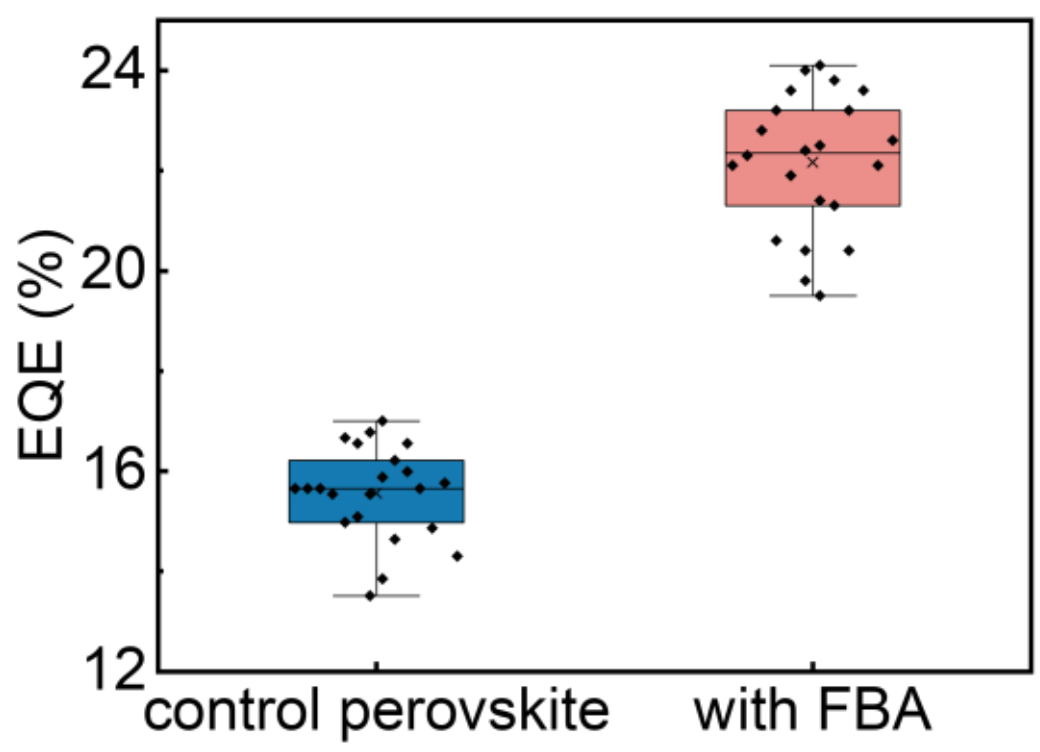

Fig. S39: Peak-EQE distributions for PeLEDs containing control perovskite or FBA-modified perovskite as the emissive layer deposited on PVK-F 70% blend/PVK/PVP HTL configuration. The average peak EQEs are 15.6% and 22.1% for control perovskite and FBA-modified perovskite, respectively.

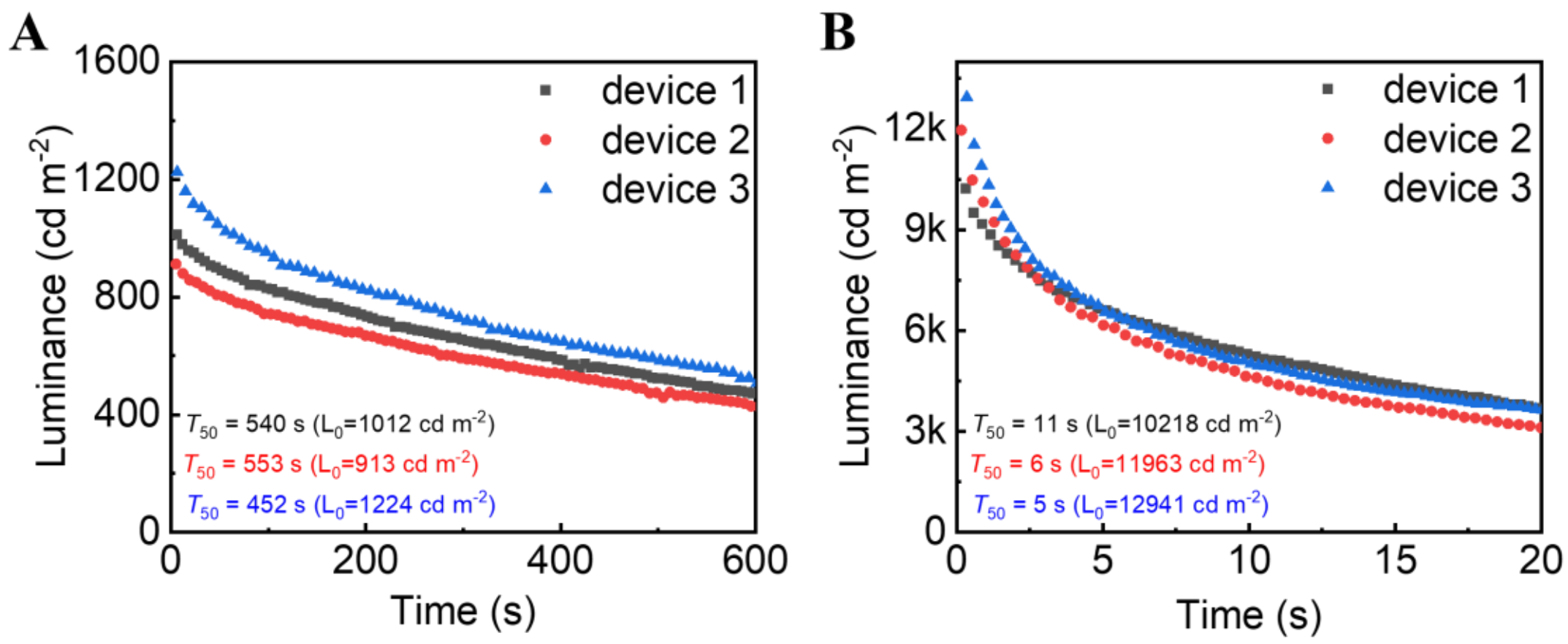

Fig. S40: Operational stability of PeLED with FBA-modified perovskite as emitting layer and fabricated on PVK-F 70% blend/PVK/PVP HTL configuration at different initial luminances. (A), 1000 cd m^{-2} and (B), 10,000 cd m^{-2}.

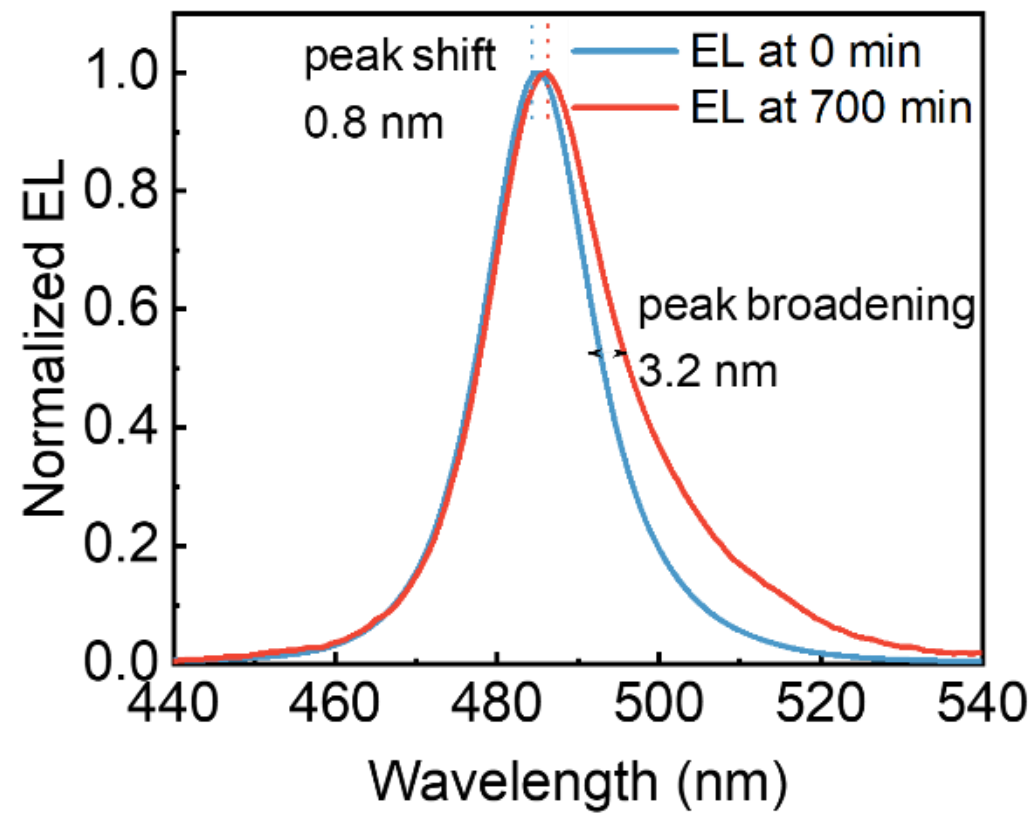

Fig. S41: EL spectral evolution for PeLEDs with FBA-modified perovskite as emitting layer and prepared on PVK-F 70% blend/PVK/PVP HTL configuration before and after biasing at an initial luminance of 100 cd m^{-2}.

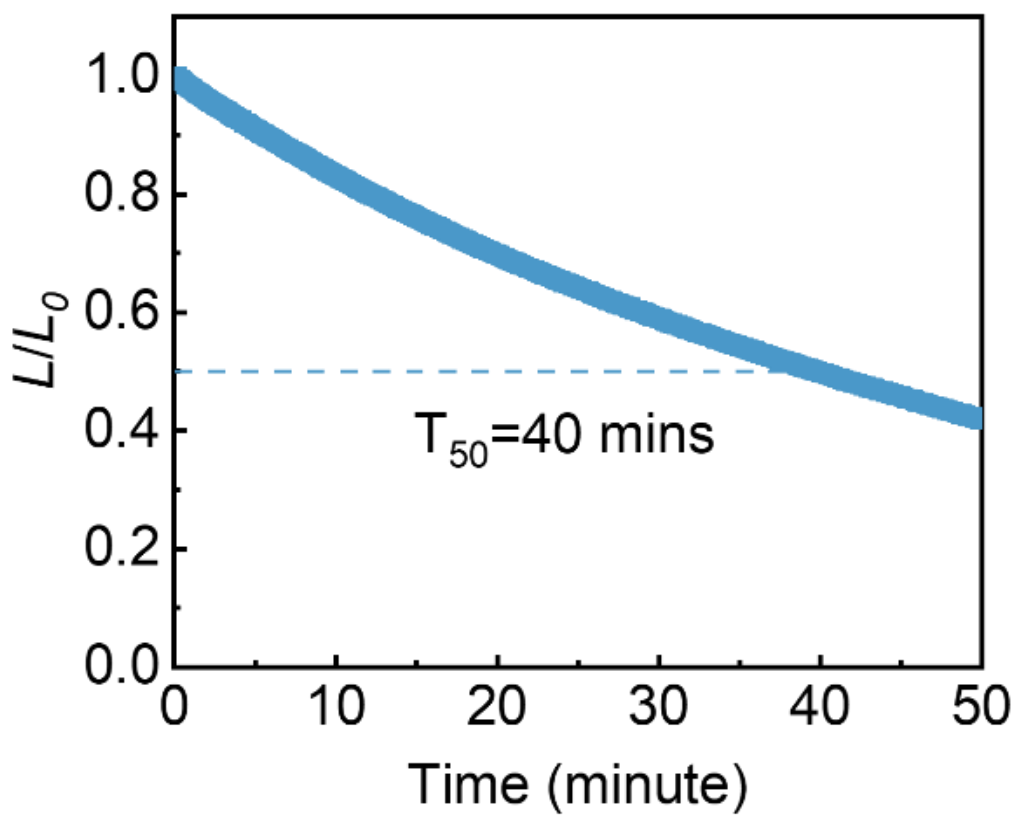

Fig. S42: Operational stability of PeLED with FBA-modified perovskite as emitting layer and fabricated on PVK/PVP HTL configuration at an initial luminance of 100 cd m^{-2}.

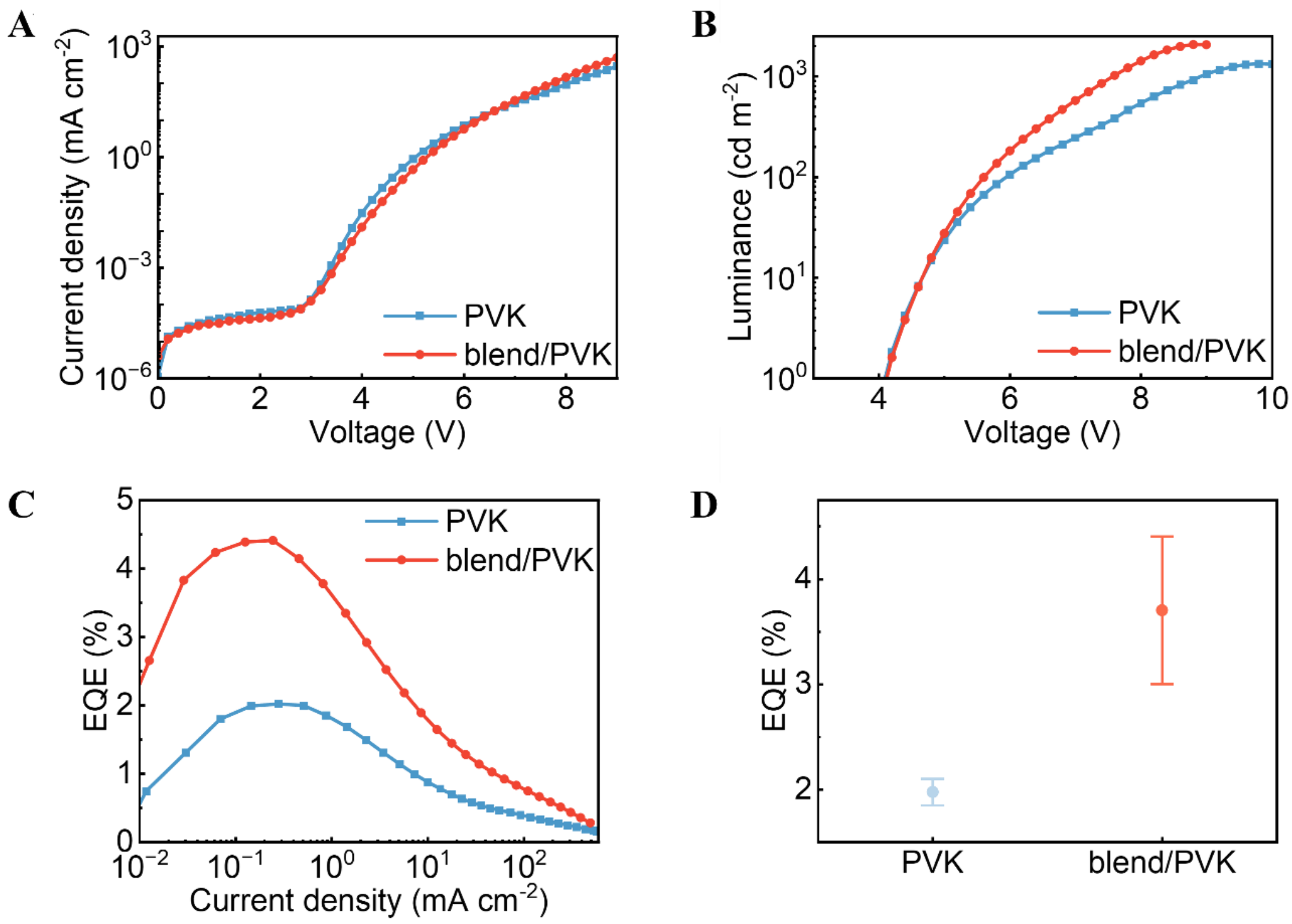

Fig. S43: Device performance comparison for quasi-2D perovskite composition deposited on either PVK/PVP or PVK-F 70% blend/PVK/PVP HTL configurations. (A), Current density versus voltage characteristics. (B), Luminance versus voltage characteristics. (C), The champion device EQE, and (D), Statistical comparison of device EQE based on different HTL configurations among individual 9 devices.

Table S1: Summary of performance and their device structure for blue PeLEDs emitting between 478 nm and 494 nm.

Perovskite	EL peak(nm)	EQE (%)	EQE at 100 cd m^{-2}	Luminance (cd m^{-2})	T_{50} (min)	Pixel area (mm^2)	FWHM (nm)	HTL	ETL	Ref
NC	480	26.4	~17	1601	1.5	/	17	PEDOT:PSS:PFI/PF8Cz	TPBi/PO-T2T/LiF/Al	(*11*)
QD	490	23.5	/	2843	43.4	/	16	PEDOT:PSS/TFB+TCTa	TPBi/PO-T2T/LiF/Al	(*10*)
QD	478	18.0	~17	3000	126	/	21	PEDOT:PSS or NiOx/PVK	TPBi /LiF/Al	(*18*)
QD	490	14.3	/	406	6	/	19	PEDOT:PSS/Poly-TPD	TPBi /LiF/Al	(*54*)
Quasi-2D	483	21.4	~18	1980	139	10	24	PVK/PVP	TPBi /Liq/Al	(*13*)
Quasi-2D	487	14.8	~12	3131	48	10	/	PEDOT:PSS	TPBi /LiF/Al	(*55*)
Quasi-2D	486	16.1	~13	2531	12.5	10	23	PEDOT:PSS	TPBi /LiF/Al	(*56*)
Quasi-2D	488	12.2	~7	8600	160	3	/	PEDOT:PSS	TPBi /LiF/Al	(*57*)
Quasi-2D	483	18.7	~9	1147	4	10	25	PEDOT:PSS/PVK	CN-T2T/LiF/Al	(*58*)
Quasi-2D	490	18.8	~15	2046	10	10	20	PEDOT:PSS	TPBi /LiF/Al	(*59*)
Quasi-2D	486	16.2	~12	1060	10	/	/	NiOx/PVK	TPBi /LiF/Al	(*31*)
Quasi-2D	494	21.2	~16	1500	27.8	9	24	PEDOT:PSS	TPBi /LiF/Al	(*60*)
Quasi-2D	488	21.6	~20	10030	1	4	23	PTAA/PVK	PO-T2T /B_3PYMPM/LiF/Al	(*61*)
Quasi-2D	488	17.5	/	3521	/	10	/	PVK	TPBi /Liq/Al	(*62*)
Quasi-2D	488	17.3	~15	2708	5	10	22	PEDOT:PSS/PVK	TPBi /LiF/Al	(*63*)
Quas	486	15.	~13	7000	515	/	21	PEDOT:PSS	TPBi	(*64*)

i-2D		6							/LiF/Al	
Quasi-2D	493	17	~14	2540	12	10	24	PEDOT:PSS	TPBi /LiF/Al	(*65*)
Quasi-2D	478	17.3	~10	547	5	10	22	PEDOT:PSS	TPBi /LiF/Al	(*66*)
Quasi-2D	488	16.4	~14	8290	16	/	/	PEDOT:PSS/ PMMA/GASCN	TPBi /Liq/Al	(*67*)
Quasi-2D	488	23.2	~20	1189	33.2	/	25	PVK/2PACz	TPBi /LiF/Al	(*68)*
Quasi-2D	489	22.5	~20	~1500	150	/	20.8	PEDOT:PSS	CN-T2T/LiF/Al	(*69*)
Quasi-2D	490	22.9	~17	~2000	6.5	/	22	PEDOT:PSS	PO-T2T/TPBi /LiF/Al	(*70*)
Quasi-2D	492	20.8	~18	14696	38.7	/	/	PEDOT:PSS/ PVK/MPC	TPBi /LiF/Al	(*71*)
Quasi-2D	485	24.0	~24	4384	13	10	23	PEDOT:PSS	3TPYMB /LiF/Al	(*19*)
3D	487	23.3	~20	5700	42	3	/	NiOx/MeO-2PACz/TFB/ PVK	TPBi /LiF/Al	(*12*)
3D	485	24.1	23.8	26000	735	4.5	14	Polymer blend/PVK/PVP	TPBi /LiF/Al	this work

Other Supplementary Materials for this manuscript include the following:
Movies S1 to S4

Movie S1: Side view of dipole propagation in planar emissive layer devices by FDTD simulation.

Movie S2: Top view of dipole propagation in planar emissive layer devices by FDTD simulation.

Movie S3: Side view of dipole propagation in isolated island-shaped emissive layer devices by FDTD simulation.

Movie S4: Top view of dipole propagation in isolated island-shaped emissive layer devices by FDTD simulation.

REFERENCES

1. S. D. Stranks, H. J. Snaith, Metal-halide perovskites for photovoltaic and light-emitting devices. *Nat. Nanotechnol.* **10**, 391–402 (2015).

2. X. K. Liu, W. Xu, S. Bai, Y. Jin, J. Wang, R. H. Friend, F. Gao, Metal halide perovskites for light-emitting diodes. *Nat. Mater.* **20**, 10–21 (2021).

3. J. Zhang, B. Cai, X. Zhou, F. Yuan, C. Yin, H. Wang, H. Chen, X. Ji, X. Liang, C. Shen, Y. Wang, Z. Ma, J. Qing, Z. Shi, Z. Hu, L. Hou, H. Zeng, S. Bai, F. Gao, Ligand-induced cation–π interactions enable high-efficiency, bright, and spectrally stable Rec. 2020 pure-red perovskite light-emitting diodes. *Adv. Mater.* **35**, 2303938 (2023).

4. M. Li, Y. Yang, Z. Kuang, C. Hao, S. Wang, F. Lu, Z. Liu, J. Liu, L. Zeng, Y. Cai, Y. Mao, J. Guo, H. Tian, G. Xing, Y. Cao, C. Ma, N. Wang, Q. Peng, L. Zhu, W. Huang, J. Wang, Acceleration of radiative recombination for efficient perovskite LEDs. *Nature* **630**, 631–635 (2024).

5. J. S. Kim, J. M. Heo, G. S. Park, S. J. Woo, C. Cho, H. J. Yun, D. H. Kim, J. Park, S. C. Lee, S. H. Park, E. Yoon, N. C. Greenham, T. W. Lee, Ultra-bright, efficient and stable perovskite light-emitting diodes. *Nature* **611**, 688–694 (2022).

6. Y. Deng, F. Peng, Y. Lu, X. Zhu, W. Jin, J. Qiu, J. Dong, Y. Hao, D. Di, Y. Gao, T. Sun, M. Zhang, F. Liu, L. Wang, L. Ying, F. Huang, Y. Jin, Solution-processed green and blue quantum-dot light-emitting diodes with eliminated charge leakage. *Nat. Photonics* **16**, 505–511 (2022).

7. X. C. Fan, K. Wang, Y. Z. Shi, Y. C. Cheng, Y. T. Lee, J. Yu, X. K. Chen, C. Adachi, X. H. Zhang, Ultrapure green organic light-emitting diodes based on highly distorted fused π-conjugated molecular design. *Nat. Photonics* **17**, 280–285 (2023).

8. S. O. Jeon, K. H. Lee, J. S. Kim, S. G. Ihn, Y. S. Chung, J. W. Kim, H. Lee, S. Kim, H. Choi, J. Y. Lee, High-efficiency, long-lifetime deep-blue organic light-emitting diodes. *Nat. Photonics* **15**, 208–215 (2021).

9. Z. Yu, Y. Deng, J. Ye, L. van Turnhout, T. Liu, A. Tew, R. Arul, S. Dowland, Y. Sun, X. Li, L. Dai, Y. Lu, C. Ducati, J. J. Baumberg, R. H. Friend, R. L. Z. Hoye, A. Rao, Triplets electrically turn on insulating lanthanide-doped nanoparticles. *Nature* **647**, 625–631 (2025).

10. Y. Nong, J. Yao, J. Li, L. Xu, Z. Yang, C. Li, J. Song, Boosting external quantum efficiency of blue perovskite QLEDs exceeding 23% by trifluoroacetate passivation and mixed hole transportation design. *Adv. Mater.* **36**, e2402325 (2024).

11. Y. Gao, Q. Cai, Y. He, D. Zhang, Q. Cao, M. Zhu, Z. Ma, B. Zhao, H. He, D. Di, Z. Ye, X. Dai, Highly efficient blue light-emitting diodes based on mixed-halide perovskites with reduced chlorine defects. *Sci. Adv.* **10**, 5645 (2024).

12. Y. Chen, R. Wang, G. Kusch, B. Xu, C. Hao, C. Xue, L. Cheng, L. Zhu, J. Wang, H. Li, R. A. Oliver, N. Wang, W. Huang, J. Wang, All-site alloyed perovskite for efficient and bright blue light-emitting diodes. *Nat. Commun.* **16**, 3254 (2025).

13. S. Yuan, L. Dai, Y. Sun, F. Auras, Y. H. Zhou, R. Z. An, Y. Liu, C. Ding, C. Cassidy, X. Tang, S. C. Dong, H. B. Kang, K. Chen, X. Liu, Z. F. Ye, Y. Zhao, C. Adachi, L. S. Liao, N. C. Greenham, Y. Qi, S. D. Stranks, L. S. Cui, R. H. Friend, Efficient blue electroluminescence from reduced-dimensional perovskites. *Nat. Photonics* **18**, 425–431 (2024).

14. K. Ji, W. Lin, Y. Sun, L. S. Cui, J. Shamsi, Y. H. Chiang, J. Chen, E. M. Tennyson, L. Dai, Q. Li, K. Frohna, M. Anaya, N. C. Greenham, S. D. Stranks, Self-supervised deep learning for tracking degradation of perovskite light-emitting diodes with multispectral imaging. *Nat. Mach. Intell.* **5**, 1225–1235 (2023).

15. M. Karlsson, Z. Yi, S. Reichert, X. Luo, W. Lin, Z. Zhang, C. Bao, R. Zhang, S. Bai, G. Zheng, P. Teng, L. Duan, Y. Lu, K. Zheng, T. Pullerits, C. Deibel, W. Xu, R. Friend, F. Gao, Mixed halide perovskites for spectrally stable and high-efficiency blue light-emitting diodes. *Nat. Commun.* **12**, 361 (2021).

16. A. J. Mirabelli, B. Kammlander, Y. Lu, R. M. Varma, Q. Gu, K. Radetzky, T. A. Selby, T. Liu, S. Riva, Z. Wei, T. L. Lee, J. Rawle, H. Rensmo, M. Anaya, U. B. Cappel, S. D. Stranks,

Interfacial chemistry limits the stability of deep blue perovskite LEDs revealed by operando characterization. *ACS Energy Lett.* **10**, 3533–3543 (2025).

17. X. Li, Q. Gu, W. Huang, S. M. Fairclough, R. H. Friend, S. D. Stranks, T. Liu, C. Ducati, Multimodal electron microscopy of halide perovskite interfacial dynamics. *Nature* **651**, 614–620 (2026).

18. Y. Jiang, C. Sun, J. Xu, S. Li, M. Cui, X. Fu, Y. Liu, Y. Liu, H. Wan, K. Wei, T. Zhou, W. Zhang, Y. Yang, J. Yang, C. Qin, S. Gao, J. Pan, Y. Liu, S. Hoogland, E. H. Sargent, J. Chen, M. Yuan, Synthesis-on-substrate of quantum dot solids. *Nature* **612**, 679–684 (2022).

19. Z. Chu, W. Zhang, J. Jiang, Z. Xia, M. Shi, B. Li, L. Dai, Y. Shen, Y. Li, N. C. Greenham, R. H. Friend, X. Zhang, J. You, Blue perovskite light-emitting diodes using multifunctional small molecule dopants. *Adv. Mater.* **37**, e2409718 (2025).

20. H. Tsai, S. Shrestha, R. A. Vilá, W. Huang, C. Liu, C. H. Hou, H. H. Huang, X. Wen, M. Li, G. Wiederrecht, Y. Cui, M. Cotlet, X. Zhang, X. Ma, W. Nie, Bright and stable light-emitting diodes made with perovskite nanocrystals stabilized in metal–organic frameworks. *Nat. Photonics* **15**, 843–849 (2021).

21. P. Gao, Z. Chen, S. Chen, Electron-induced degradation in blue quantum-dot light-emitting diodes. *Adv. Mater.* **36**, e2309123 (2024).

22. X. Luo, W. Xu, G. Zheng, S. Tammireddy, Q. Wei, M. Karlsson, Z. Zhang, K. Ji, S. Kahmann, C. Yin, Y. Zou, Z. Zhang, H. Chen, L. A. B. Marçal, H. Zhao, D. Ma, D. Zhang, Y. Lu, M. Li, C. Deibel, S. D. Stranks, L. Duan, J. Wallentin, W. Huang, F. Gao, Effects of local compositional heterogeneity in mixed halide perovskites on blue electroluminescence. *Matter* **7**, 1054–1070 (2024).

23. M. Pylnev, A. M. Barbisan, T. C. Wei, Effect of wettability of substrate on metal halide perovskite growth. *Appl. Surf. Sci.* **541**, 148559 (2021).

24. Y. Seop Shin, Y. Jin Yoon, S. Hyeon Kweon, S. Hak Oh, C. Beom Park, D. Yuk, T. Song, J. Geon Son, J. Seo, W. Lee, S. Kyu Kwak, G. H. Kim, J. Young Kim, Controlling the

formation energy of perovskite phase via surface polarity of substrate for efficient pure-blue light-emitting diodes. *Chem. Eng. J.* **464**, 142707 (2023).

25. F. Zhang, Y. Gao, P. Lu, Y. Zhong, Y. Liu, X. Bao, Z. Xu, M. Lu, Y. Wu, P. Chen, J. Hu, Y. Zhang, Z. Wu, H. Song, X. Bai, Engineering of hole transporting interface by incorporating the atomic-precision Ag6 nanoclusters for high-efficiency blue perovskite light-emitting diodes. *Nano Lett.* **23**, 1582–1590 (2023).

26. D. Yang, B. Zhao, T. Yang, R. Lai, D. Lan, R. H. Friend, D. Di, Toward stable and efficient perovskite light-emitting diodes. *Adv. Funct. Mater.* **32**, 2109495 (2022).

27. X. Yan, B. Wu, C. Chen, F. Sun, H. Bao, S. Chang, H. Zhong, S. Jin, W. Tian, Elucidating the impact of electron accumulation in quantum-dot light-emitting diodes. *Nano Lett.* **24**, 13374–13380 (2024).

28. R. Chen, H. Chen, Y. Yu, C. Luo, C. Xu, X. Zhou, Z. Ren, Y. Chen, Sufficient hole injection for high-performance blue perovskite light-emitting diodes. *ACS Photonics* **11**, 5380–5387 (2024).

29. S. Lee, D. Bin Kim, I. Hamilton, M. Daboczi, Y. S. Nam, B. R. Lee, B. Zhao, C. H. Jang, R. H. Friend, J. S. Kim, M. H. Song, Control of interface defects for efficient and stable quasi-2D perovskite light-emitting diodes using nickel oxide hole injection layer. *Adv. Sci.* **5**, 1801350 (2018).

30. H. Zhang, C. Zhao, J. Yao, W. C. H. Choy, Dopant-free NiO_x nanocrystals: A low-cost and stable hole transport material for commercializing perovskite optoelectronics. *Angew. Chem. Int. Ed.* **62**, e202219307 (2023).

31. P. Shen, S. Ding, Z. Tang, L. Qian, T. Zhang, P. Xiao, T. Chen, H. Chen, X. Zhang, Y. Ren, D. Zhao, C. Xiang, High-n phase suppression for efficient and stable blue perovskite light-emitting diodes. *Adv. Sci.* **11**, e2306167 (2024).

32. A. Liu, Z. Zhang, J. Li, H. Yu, N. Wang, J. Wang, N. Zhao, Optimizing perovskite surfaces to enhance post-treatment for efficient blue mixed-halide perovskite light-emitting diodes. *Adv. Mater.* **37**, 2414788 (2024).

33. S. Wang, Z. Yu, J. Qin, G. Chen, Y. Liu, S. Fan, C. Ma, F. Yao, H. Cui, S. Zhou, K. Dong, Q. Lin, C. Tao, F. Gao, W. Ke, G. Fang, Buried interface modification and light outcoupling strategy for efficient blue perovskite light-emitting diodes. *Sci. Bull.* **69**, 2231–2240 (2024).

34. T.-H. Do, A. Loganathan, Y.-H. Wu, Y.-S. Fu, T.-F. Guo, Unanticipated interfacial redox reaction in the NiO_x transport layer of perovskite light-emitting diodes. *Adv. Sci.* **13**, e10824 (2025).

35. Y. Cheng, M. Li, X. Liu, S. H. Cheung, H. T. Chandran, H. W. Li, X. Xu, Y. M. Xie, S. K. So, H. L. Yip, S. W. Tsang, Impact of surface dipole in NiO_x on the crystallization and photovoltaic performance of organometal halide perovskite solar cells. *Nano Energy* **61**, 496–504 (2019).

36. Z. Li, Z. Chen, Z. Shi, G. Zou, L. Chu, X. K. Chen, C. Zhang, S. K. So, H. L. Yip, Charge injection engineering at organic/inorganic heterointerfaces for high-efficiency and fast-response perovskite light-emitting diodes. *Nat. Commun.* **14**, 6441 (2023).

37. H. Choe, D. Jeon, S. J. Lee, J. Cho, Mixed or segregated: Toward efficient and stable mixed halide perovskite-based devices. *ACS Omega* **6**, 24304–24315 (2021).

38. M. Petrović, T. Maksudov, A. Panagiotopoulos, E. Serpetzoglou, I. Konidakis, M. M. Stylianakis, E. Stratakis, E. Kymakis, Limitations of a polymer-based hole transporting layer for application in planar inverted perovskite solar cells. *Nanoscale Adv.* **1**, 3107–3118 (2019).

39. B. Kang, M. Jang, Y. Chung, H. Kim, S. K. Kwak, J. H. Oh, K. Cho, Enhancing 2D growth of organic semiconductor thin films with macroporous structures via a small-molecule heterointerface. *Nat. Commun.* **5**, 4752 (2014).

40. Z. Yu, W. H. Jeong, K. Kang, H. Song, X. Shen, H. Ahn, S. W. Lee, X. Fan, J. W. Jang, S. R. Ha, J. W. Min, J. H. Park, J. Han, E. D. Jung, M. H. Song, D. W. Chang, W. Bin Im, S. H.

Park, H. Choi, B. R. Lee, A polymer/small-molecule binary-blend hole transport layer for enhancing charge balance in blue perovskite light emitting diodes. *J. Mater. Chem. A Mater.* **10**, 13928–13935 (2022).

41. N. Leclerc, P. Chávez, O. A. Ibraikulov, T. Heiser, P. Lévêque, Impact of backbone fluorination on π-conjugated polymers in organic photovoltaic devices: A review. *Polymers* **8**, 11 (2016).

42. Y. Mizuno, I. Takasu, S. Uchikoga, S. Enomoto, T. Sawabe, A. Amano, A. Wada, T. Sugizaki, J. Yoshida, T. Ono, C. Adachi, Fluorinated carbazole derivatives as wide-energy-gap host material for blue phosphorescent organic light-emitting diodes. *J. Phys. Chem. C* **116**, 20681–20687 (2012).

43. S. Suárez-Suárez, G. A. Carriedo, A. Presa Soto, Porous films by the self-assembly of inorganic rod-b-coil block copolymers: Mechanistic insights into the vesicle-to-pore morphological evolution. *Soft Matter* **12**, 3084–3092 (2016).

44. Y. Su, X. Wang, S. Zhou, H. Sun, B. Wang, D. Liu, G. Zhu, Porous rigid-flexible polymer membrane interface towards high-rate and stable zinc-ion battery. *J. Power Sources* **560**, 232685 (2023).

45. E. Bormashenko, Progress in understanding wetting transitions on rough surfaces. *Adv. Colloid Interface Sci.* **222**, 92–103 (2015).

46. V. H. Dalvi, P. J. Rossky, Molecular origins of fluorocarbon hydrophobicity. *Proc. Natl. Acad. Sci. U.S.A.* **107**, 13603–13607 (2010).

47. H. Kim, J. Cho, Y. Park, T. Y. Seong, Leakage current origins and passivation effect of GaN-based light emitting diodes fabricated with Ag p -contacts. *Appl. Phys. Lett.* **92**, 92115 (2008).

48. Y. Ling, Y. Tian, X. Wang, J. C. Wang, J. M. Knox, F. Perez-Orive, Y. Du, L. Tan, K. Hanson, B. Ma, H. Gao, Enhanced optical and electrical properties of polymer-assisted all-inorganic perovskites for light-emitting diodes. *Adv. Mater.* **28**, 8983–8989 (2016).

49. V. V. Sharma, Y. S. Shin, J. Y. Kim, D. S. Kim, G. H. Kim, Hole transport layer engineering in high performance quasi-2D perovskite blue light emitting diodes. *Nanoscale* **16**, 11368–11383 (2024).

50. Y. Feng, M. Zhu, Q. Cao, H. Li, X. Zhu, X. Lyu, X. Dai, H. He, Z. Ye, p-π Conjugated L-Type ligand for pure-red perovskite light-emitting diodes. *ACS Energy Lett.* **9**, 1125–1132 (2024).

51. J. C. De Mello, H. F. Wittmann, R. H. Friend, An improved experimental determination of external photoluminescence quantum efficiency. *Adv. Mater.* **9**, 230–232 (1997).

52. T. Lu, Q. Chen, van der Waals potential: An important complement to molecular electrostatic potential in studying intermolecular interactions. *J. Mol. Model.* **26**, 315 (2020).

53. S. Grimme, S. Ehrlich, L. Goerigk, Effect of the damping function in dispersion corrected density functional theory. *J. Comput. Chem.* **32**, 1456–1465 (2011).

54. H. Zhu, G. Tong, J. Li, E. Xu, X. Tao, Y. Sheng, J. Tang, Y. Jiang, Enriched-bromine surface state for stable sky-blue spectrum perovskite QLEDs With an EQE of 14.6%. *Adv. Mater.* **34**, e2205092 (2022).

55. Y. Shen, Y. Q. Li, K. Zhang, L. J. Zhang, F. M. Xie, L. Chen, X. Y. Cai, Y. Lu, H. Ren, X. Gao, H. Xie, H. Mao, S. Kera, J. X. Tang, Multifunctional crystal regulation enables efficient and stable sky-blue perovskite light-emitting diodes. *Adv. Funct. Mater.* **32**, 2206574 (2022).

56. Z. Chu, W. Zhang, J. Jiang, Z. Qu, F. Ma, Y. Zhao, X. Chu, Y. Shen, Y. Li, Z. Yin, X. Zhang, J. You, Blue light-emitting diodes based on quasi-two-dimensional perovskite with efficient charge injection and optimized phase distribution via an alkali metal salt. *Nat. Electron.* **6**, 360–369 (2023).

57. M. Alahbakhshi, A. Mishra, G. Verkhogliadov, E. E. Turner, R. Haroldson, A. C. Adams, Q. Gu, J. J. Rack, J. D. Slinker, A. A. Zakhidov, Highly efficient quasi 2D blue perovskite electroluminescence leveraging a dual ligand composition. *Adv. Funct. Mater.* **33**, 2214315 (2023).

58. W. Zhou, Y. Shen, L. X. Cao, Y. Lu, Y. Y. Tang, K. Zhang, H. Ren, F. M. Xie, Y. Q. Li, J. X. Tang, Manipulating ionic behavior with bifunctional additives for efficient sky-blue perovskite light-emitting diodes. *Adv. Funct. Mater.* **33**, 2301425 (2023).

59. X. M. Hu, Y. Shen, S. C. Feng, Z. H. Su, K. Zhang, L. X. Cao, B. F. Wang, X. Gao, J. X. Tang, Y. Q. Li, Unveiling the role of additive molecular characteristics in regulating chlorine loss and phase distribution for blue perovskite light-emitting diodes. *Adv. Funct. Mater.* **34**, 2410143 (2024).

60. H. Wang, B. Zhang, B. Wang, S. Bai, L. Cheng, Y. Hu, S. Lu, Efficient quasi-2D perovskite based blue light-emitting diodes with carbon dots modified hole transport layer. *Nano Lett.* **24**, 8702–8708 (2024).

61. M. Jiang, X. Zhang, F. Wang, Efficient perovskite nanograin light-emitting diodes in green-to-blue gamut with Co-additive engineering. *Adv. Mater.* **36**, e2400565 (2024).

62. Y. Xia, B. Song, Z. Zhang, K. L. Wang, Y. H. Li, N. Li, C. H. Chen, J. Chen, G. Xing, Z. K. Wang, Vertically concentrated quantum wells enabling highly efficient deep-blue perovskite light-emitting diodes. *Angew. Chem. Int. Ed. Engl.* **63**, e202403739 (2024).

63. L. Wang, Z. H. Su, Y. Shen, S. C. Feng, F. M. Xie, K. Zhang, K. F. Meng, X. Gao, J. X. Tang, Y. Q. Li, Regulated perovskite crystallization for efficient blue light-emitting diodes via interfacial molecular network. *Adv. Funct. Mater.* **34**, 2401297 (2024).

64. F. Lu, J. Dong, J. Zhou, N. Wang, Enhancing energy transfer via inhibition defect density of coupled quasi-2D perovskite layers for efficient sky-blue light-emitting diodes. *Chem. Eng. J.* **485**, 149887 (2024).

65. Z. Bao, S. Yu, X. Guo, Y. Wang, Y. Lv, D. Zou, L. Song, X. Liu, Regulating the continuous and concentrated distribution of Quasi-2D perovskite phases to achieve Sky-Blue Light-Emitting diodes with efficiency approaching 17%. *Chem. Eng. J.* **482**, 148875 (2024).

66. Y. Yu, B. F. Wang, Y. Shen, Z. H. Su, K. Zhang, H. Ren, Y. F. Zhang, X. Gao, J. X. Tang, Y. Q. Li, Regulating perovskite crystallization through interfacial engineering using a

zwitterionic additive potassium sulfamate for efficient pure-blue light-emitting diodes. *Angew. Chem. Int. Ed. Engl.* **63**, e202319730 (2024).

67. F. Zhang, Y. Gao, D. Wang, P. Lu, X. Wang, M. Lu, Y. Wu, P. Chen, J. Hu, X. Bai, Z. Wu, D. Zhou, D. Liu, L. Xu, B. Dong, H. Song, Y. Zhang, Phase distribution management for high-efficiency and bright blue perovskite light-emitting diodes. *Nano Energy* **120**, 109144 (2024).

68. S. Q. Sun, Y. Xu, Q. Sun, T. Bian, W. Z. Liu, B. C. Liu, M. Zhu, J. J. Bao, J. Yin, L. S. Liao, Y. M. Xie, S. T. Lee, M. K. Fung, Phase-pure quasi-two-dimensional layered perovskites enable efficient blue light-emitting diodes. *Nano Lett.* **25**, 16287–16295 (2025).

69. J. S. Kim, H. D. Lee, J. H. Jung, T. Kim, S. J. Woo, H. J. Yoon, J. Moon, C. M. Kang, S. E. Chang, D. H. Kim, S. Kim, H. Yang, D. Kim, T. W. Lee, Kinetically-controlled intermediate-direct-pinning for homogeneous energy landscapes in quasi-two-dimensional perovskites for efficient and narrow blue emission. *Nat. Commun.* **16**, 9590 (2025).

70. B. F. Wang, Y. Yu, Y. Shen, Y. H. Wu, Z. H. Su, Y. T. Wang, Y. H. Zhang, L. X. Cao, X. Y. Gao, J. X. Tang, Y. Q. Li, Enhanced electroluminescence and stability of sky-blue perovskite light-emitting diodes. *Angew. Chem. Int. Ed. Engl.* **64**, e202419746 (2025).

71. Y. Liu, H. Wang, F. Zhang, X. Chai, K. Liu, S. Zhang, M. Kong, H. Liu, X. Wang, M. Lu, Y. Yang, Z. Wu, Y. Zhang, C. Fan, X. Bai, High-efficiency and stable pure bromine blue light-emitting diodes enabled by hybrid quasi-2D perovskite. *Adv. Funct. Mater.* **36**, e15841 (2025).
